# A systematic review exploring the evidence reported to underpin exercise dose in clinical trials of rheumatoid arthritis

**DOI:** 10.1093/rheumatology/keaa150

**Published:** 2020-08-11

**Authors:** Graham Boniface, Varsha Gandhi, Meriel Norris, Esther Williamson, Shona Kirtley, Neil E O’Connell

**Affiliations:** k1 Nuffield Department of Orthopaedics Rheumatology and Musculoskeletal Sciences (NDORMS), Centre for Rehabilitation Research in Oxford (RRIO), University of Oxford, Oxford; k2 Department of Clinical Sciences, Brunel University London, Uxbridge, UK

**Keywords:** rheumatoid arthritis, exercise, dose response, RCT, systematic review, intervention

## Abstract

We aimed to evaluate the evidence reported to underpin exercise dose in randomised controlled trials (RCTs) using strengthening exercise in RA. We searched six different databases between 1 January 2000 and 3 April 2019. We included RCTs, where a main component of the intervention and/or control used strengthening exercise. Evidence sources cited to underpin dose were judged for their quality, consistency and applicability. Thirty-two RCTs were reviewed. Four (12.5%) piloted the intervention without using dose-escalation designs to determine optimal dose-response. Twenty (62.5%) reported no evidence underpinning dose. Where reported, quality, consistency and applicability of the underpinning evidence was a cause for methodological concern. The majority of RCTs did not report the evidence underpinning dose. When reported, the evidence was often not applicable to the clinical population. Frequently, the dose used differed to the dose reported/recommended by the underpinning evidence. Our findings illustrate exercise dose may not be optimised for use with clinical populations prior to evaluation by RCT.

**Rheumatology key messages**

- The majority of exercise trials in RA don’t report evidence underpinning dose of strengthening exercise.
- Exercise trials in RA seldom pilot their interventions to determine dose-response.
- Evidence used by exercise trials in RA to underpin dose is often low in quality.

## Introduction

In the United Kingdom (UK), exercise is recommended by clinical guidelines alongside pharmaceutical interventions for the management of RA [1]. One type of exercise that has grown in popularity, with more clinical trials evaluating its safety and effectiveness being published, is strengthening exercise [2]. Once considered to be detrimental for people living with RA because it was thought to cause damage to the joints [3], strengthening exercise is commonly used to counter the cachectic effects (muscle wasting) of the disease [4]. However, uncertainty exists regarding what exercise dose is most effective for improving the RA symptoms, function and other patient-centred outcomes [5]. Dose refers to the amount of treatment prescribed [6], and exercise dose is made up of the following parameters: exercise type, sets, repetitions, load and/or intensity, recovery time/method of progression, session duration, frequency of exercise sessions and duration of the evaluated programme [7, 8]. A recent research priority setting partnership identified that establishing the most effective dose of treatments such as exercise is a key research priority [9]. Exercise dose is a critical methodological consideration when designing a clinical trial as it may be a key driver for producing positive outcomes [10–14].

The randomised controlled trial (RCT) is a robust approach for evaluating the effectiveness of clinical interventions [15]. A critical stage when developing an intervention commonly centres on establishing a safe dose and schedule of administration [16]. The approaches used to achieve this may differ depending on the type of intervention being tested. Researchers evaluating new drugs commonly use early phase clinical trials (e.g. phase-I/II) employing different dose-escalation designs [17] as an essential step for safeguarding participants and optimising potential for efficacy [18]. Conversely, those evaluating exercise-based interventions seldom use this approach and may choose to follow the Medical Research Council (MRC) framework for developing and evaluating RCTs for complex interventions used to improve health [19, 20]. This framework recommends researchers should consider using early phase clinical trials (e.g. pilot or feasibility) to answer key uncertainties and improve the probability of conducting a high-quality RCT [21].

In the absence of early phase clinical trials, researchers may rely on expert opinion, consensus or draw from the available evidence base (e.g. systematic reviews, RCTs, cohort studies, guidelines etc.) to determine what dose to prescribe. Examples may include the 2018 EULAR recommendations for physical activity in people with inflammatory arthritis and osteoarthritis or the ACSM’s exercise management for persons with chronic diseases [22, 23]. However, relying on these approaches may have methodological limitations. Expert opinion is rated as one of the lowest levels of evidence [24] and guidelines may lack the necessary detail about what dose to prescribe for a specific clinical population [14]. The potential impact of not formally testing dose-response and relying solely on the available evidence base is the exercise dose may not be optimised for the clinical population of interest. Consequently, evaluating the evidence researchers use to underpin dose may identify areas for methodological improvement. Therefore, we set out to investigate this further.

## Objectives of the review

- Determine what proportion of published RCTs evaluating strength-based exercise interventions in RA report using phase-I/II trials for setting dose parameters.
- Determine what type and level of evidence is used to underpin dose parameters.
- Explore the quality, consistency and applicability of the evidence used to underpin dose parameters.
- Narratively explore if a relationship exists between risk of bias for RCTs evaluating strength-based interventions in RA and the level of evidence for underpinning prescription parameters.

## Methods

The review is reported in accordance with the Preferred Reporting Items for Systematic Reviews and Meta-Analyses (PRISMA) statement. We developed the protocol and pre-registered it with the International Prospective Register of Systematic Reviews (PROSPERO): PROSPERO 2018 CRD42018090963. The full protocol has been published [25].

### Search design

We systematically searched the following databases: (1) Allied and Complimentary Medicine Database (AMED) via OVID; (2) Cochrane Central Register of Controlled Trials (CENTRAL); (3) Cumulative Index to Nursing and Allied Health Literature (CINAHL) via EBSCOhost; (4) Excerpta Medica Database (EMBASE) via OVID; (5) MEDLINE via OVID; and (6) Physiotherapy Evidence Database (PEDro). Search strategies for each of the databases were developed iteratively and included relevant controlled vocabulary terms (e.g. MeSH and EMTREE headings) and free-text terms searched in the title, abstract or keyword fields for variants of ‘rheumatoid arthritis’, ‘exercise’ and ‘stength’ or ‘resistence training’. Where available, validated RCT search filters were used including the Cochrane Highly Sensitive Search Strategy for identifying randomized trials in MEDLINE (sensitivity-maximizing version (2008 revision) Ovid format), the McMaster EMBASE RCT search filter (Best balance of sensitivity and specificity) and the SIGN Search Filter for identifying randomized trials in CINAHL (for EBSCO). We aimed to examine the state of contemporary practice in prescribing the dose of strengthening exercise in clinical trials. Therefore, we limited our search to identify RCTs published after 1 January 2000 to coincide with the year the MRC published their original framework for developing and evaluating randomized controlled trials for complex interventions used to improve health [19]. The initial search was run on 18 May 2018. We ran an update search on 3 April 2019 to identify randomized controlled trials that had been published since the initial search. We applied no language restrictions to our searches. All database search strategies used are available (Supplementary Material S1, available at *Rheumatology* online).

### Eligibility

#### Types of studies

We included all RCTs that evaluated exercise interventions where a main component (i.e. key feature) of the intervention and/or control included land-based strengthening exercise.

#### Types of participants

We included published RCTs involving adults (males and females ≥18 years old) with a diagnosis of RA. We purposefully chose not to limit the eligibility criteria for diagnosis using one of the common classification criterias (e.g. ARA 1987 revised criteria for the classification of RA or 2010 ACR-EULAR Classification Criteria for RA) in order to include as many RCTs as possible [26, 27]. We excluded trials that included participants with conditions other than RA (e.g. osteoarthritis).

#### Types of interventions

Strengthening exercise could involve the participant using equipment (e.g. free weights/machines), or their own bodyweight to provide resistance against gravity (e.g. sit-to-stand exercise). The intervention could be unsupervised (e.g. home-based), supervised (e.g. by a therapist) or both and carried out individually or in a group. The strength-based intervention could be multifactorial (e.g. used in conjunction with cointerventions like education), or multicomponent (e.g. used with other forms of exercise like aerobic or flexibility exercise).

### Data collection and analysis

#### Selection of RCTs

Two authors (G.B., V.G.) independently screened the titles and abstracts of records obtained through our database search. The title and abstract were examined and those meeting the above eligibility criteria were retrieved for further evaluation. Where ambiguity existed, resolution was achieved through consensus. If this was not possible, resolution was achieved using a third reviewer (N.E.O.).

#### Data extraction and management

Two authors (G.B., V.G.) extracted data from every included RCT. To do this, we developed and piloted an Excel data extraction form using two RCTs investigating knee osteoarthritis interventions [28, 29]. The final form collected general information about the RCT (e.g. country, clinical setting, aims/objectives etc.) and participant (e.g. age, gender, ethnicity etc.). We collected information specific to the intervention and control information using the Template for Intervention Description and Replication (TIDieR) checklist and guide [30]. We adapted item 8 (When and how much) of TIDieR, taking direction from the Consensus on Exercise Reporting Template (CERT) to extract key information about the dose of the strengthening exercise used [8]. We did this by adding exercise type, equipment used, sets, repetitions, load, intensity, method of recovery, method of progression, frequency of exercise sessions and programme duration. These dose parameters were chosen because they are important for prescribing exercise interventions in both clinical research and practice [7, 8, 31, 32]. Underpinning evidence reported to underpin the dose parameters above was identified so that its quality, consistency and applicability to the exercise dose used in the RCT could be evaluated. We did this by first looking in the section describing the intervention. If we couldn’t identify any underpinning evidence, we then proceeded to check the rest of the manuscript. Underpinning evidence was only identified for retrieval if the authors explicitly stated they had been used to develop or justify the dose of strengthening exercise. If this was not clear, resolution was achieved through consensus or recourse to a third reviewer (N.E.O.). When appropriate, we used intervention and/or protocol publications linked with the RCT to assist with extracting information about the intervention and to identify underpinning evidence sources. We then appraised the quality, consistency and applicability of the underpinning evidence.

### Process for evaluating the underpinning evidence

#### Assessment of quality

For every underpinning evidence source identified, we used the Oxford Centre for Evidence Based Medicine (OCEBM) – levels of evidence to grade its quality, using the framework’s question ‘Does this treatment help?’ [33–35]. We chose this tool because it offered a simple and standardised approach to grading evidence, something we felt could be easily understood by busy clinicians. The levels of evidence range from 1 to 5, where (1) = Systematic review of RCTs or *n*-of-1 trials, (2) = Randomized trial or observational study with dramatic effect, (3) = Non-randomized controlled cohort/follow-up study, (4) = Case-series, case-control studies, or historically controlled studies and (5) = Mechanism-based reasoning.

Grading using the above framework was relatively straightforward. However, when the evidence source cited to underpin dose was a pilot study, literature review, clinical guideline or book, to be more accurate with our grading, we had to explore the evidence used by these specific sources. Pilot studies act as the pre-cursor for the RCT, therefore to grade quality, we identified the evidence (if any) reported by the pilot study to underpin dose. For literature reviews, clinical guidelines and books, we identified (where possible) the references used by these evidence sources that were most relevant to the dose parameters reported in the RCT. These types of evidence source commonly draw on large bodies of published information to make recommendations. An example of this type of evidence source used in the exercise are the American College of Sports Medicine (ACSM) position stands [36]. Therefore, when the RCT reported using a specific part of the source to support dose, we located that part to identify the references to help with grading quality. If the RCT did not report using a specific part of the cited evidence source, we adopted a pragmatic approach, and used the dose parameters reported in the RCT to help focus our search for references. In cases where we could not grade the level of evidence, for example, the RCT reported insufficient information about the dose parameter, or the underpinning evidence source failed to reference its text clearly, we graded the quality of the underpinning evidence to be ‘unclear’. In cases where we judged the underpinning evidence not to support the dose parameter (e.g. not relevant to strengthening exercise or the reported evidence was cited incorrectly), we graded the level of evidence to be a ‘incorrect citation’.

#### Assessment of consistency

We judged consistency by comparing the dose parameters (e.g. type of exercise used, number of sets etc.) reported by the RCT to the dose parameters reported in underpinning evidence. When the dose was identical, or kept within the range reported by the underpinning evidence, we judged the RCT had been ‘consistent’ in using the same dose. When the RCT used a different dose to that reported, we judged the RCT had been ‘inconsistent’. Where the RCT and/or underpinning evidence insufficiently described the dose used, we could not make a comparison and judged it to be ‘unclear’. We approached pilot studies differently, assuming the characteristics of dose would be broadly similar. Therefore, any differences in dose were judged to be ‘inconsistent’.

#### Assessment of applicability

We judged applicability by looking for areas of homogeneity/heterogeneity across three areas: (1) Whether the underpinning evidence source was applicable to RA clinical population; (2) Whether a similar gender mix was used; and (3) Whether the mean age was similar (±10 years). We judged these areas as ‘applicable’ or ‘not applicable’. In cases where the underpinning evidence source was not a clinical trial (e.g. literature review, clinical guideline or book) and there was no definitive population to assess, we judged gender and age to be ‘not applicable’.

#### Assessment of risk of bias (RoB)

Two reviewers (G.B., V.G.) assessed risk of bias for each of the RCT sources using the six key domains of the Cochrane risk of bias tool [37]: (1) Adequate sequence generation; (2) Allocation concealment; (3) Blinding of participants and personnel; (4) Blinding of outcome assessors; (5) Incomplete outcome data; (6) Selective reporting; and (7) Other risk of bias. Due to the nature of the intervention, all included RCTs were at high risk of bias for lack of blinding of participants and personnel. However, because this bias is largely unavoidable, for the purposes of contrasting studies on their overall risk of bias, we excluded this criterion. We characterized studies to be at low RoB (all categories assessed as low RoB), unclear RoB (any category rated as unclear RoB) or high RoB (one or more categories assessed as high RoB). For secondary/tertiary evidence sources, we used the same approach where the record was a RCT. If the trial was a non-randomized controlled cohort design, we originally stipulated we would use Cochrane’s Risk Of Bias In Non-randomized Studies - of Interventions (ROBINS-I) tool [38]. However, we found that the Cochrane risk of bias tool was better suited to this task owing to the fact that the secondary/tertiary evidence sources frequently used experimental designs. We resolved disagreement through discussion, using a third reviewer (N.E.O.) when required.

## Results

**Fig. 1:**
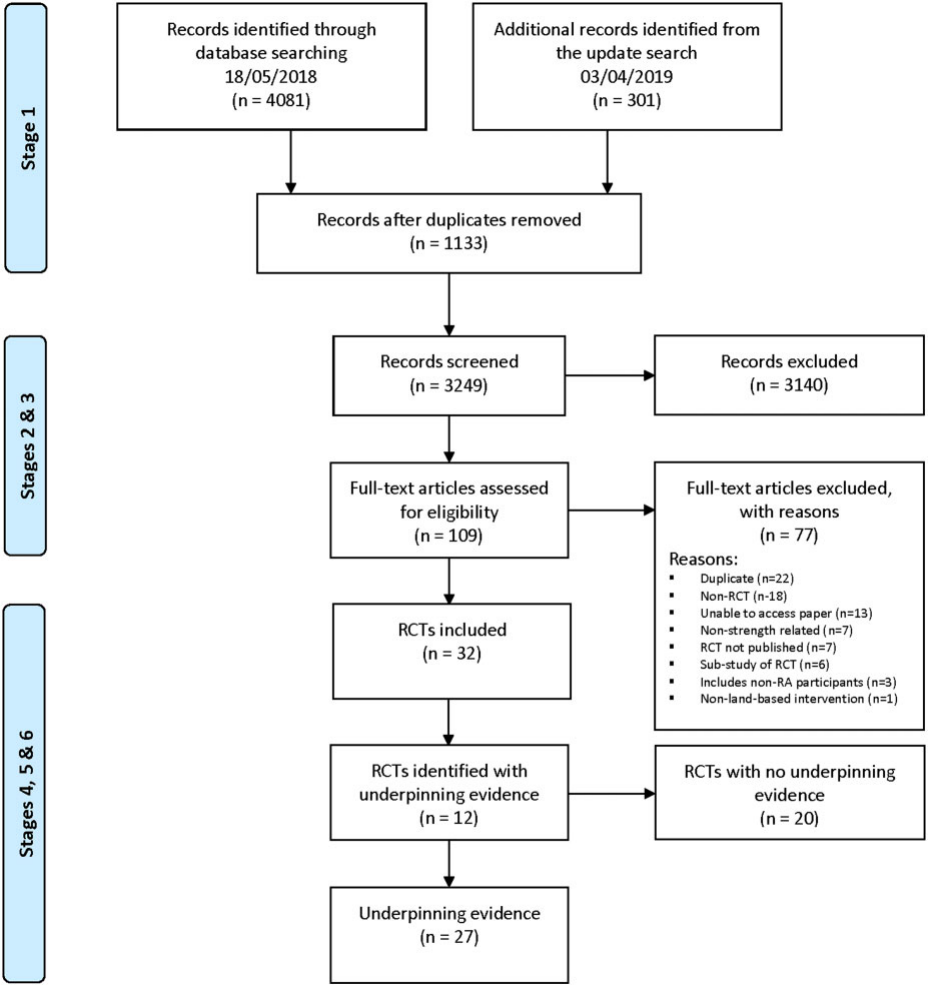
PRISMA study flow diagram

We summarize the search and screening process in the PRISMA study flow diagram (Fig. 1). A total of 4382 records were identified. Thirty-two RCTs were included [39–70]. Almeida & Piva, 2011 [71] was published as a conference abstract; however, after contacting the authors, we were provided with the full-text, which was published as Piva *et al.* 2018 [61]. We included this RCT for full-text extraction. We have reported the characteristics of the included RCTs in Supplementary Table S1.

**Table 1:** Completeness of strength exercise dose descriptions

Dose parameter	% completion
Exercise type	43%
Strength equipment used	54%
Sets	46%
Repetitions	66%
Load (e.g. kg/lb)	6%
Intensity (e.g. %1Repetition Maximum)	50%
Recovery	37%
Method of progression	63%
Frequency of sessions	97%
Programme duration	97%

### Risk of bias assessment

We assessed eight RCTs (25%) to be at overall low RoB [39, 46, 49, 53, 54, 58, 61, 68], eleven RCTs (34%) to be at unclear RoB [40, 42, 48, 50–52, 56, 57, 59, 64, 70] and thirteen (41%) RCTs to be at high RoB [41, 43–45, 47, 55, 60, 62, 63, 65–67, 69] (Supplementary Table S2, availale at *Rheumatology* online).

### RCTs reporting underpinning evidence

Twenty RCTs (62.5%) did not cite evidence to underpin prescribed dose of strengthening exercise [39–47, 49, 51, 57, 58, 62–64, 66–70]. The remaining twelve RCTs (37.5%) [48, 50, 52–56, 59–61, 65, 68] cited in total, twenty-seven evidence sources to underpin the prescribed dose of strengthening exercise (Supplementary Material S2, available at *Rheumatology* online). These included clinical trials, literature reviews, clinical guidelines, clinical opinion, books and a mobile phone application.

### Completeness of intervention descriptions

Thirty-one RCTs (97%) provided incomplete descriptions of their interventions (Supplementary Table S3, available at *Rheumatology* online). Key dose parameters were also incomplete (Table 1).

### RCTs using pilot studies

Four RCTs (12.5%) [48, 53, 54, 59] reported using a pilot study (Supplementary Table S4). We investigated whether the RCT used the same dose as its pilot study by comparing the individual dose parameters. Lamb *et al.* (2015) reported using the same dose in both the pilot and main trial. We noted inconsistencies in the dose used for Neuberger *et al.* (2007) and Lemmey *et al.* (2009). Neuberger and its pilot study reported insufficient information to compare the majority of dose parameters. We were only able to judge three parameters (method of progression, frequency of sessions and programme duration). The method used for progression was inconsistent and we were unable to locate justification for it being modified. Lemmey reported progressing dose more gradually to reduce muscle soreness when compared with its pilot study. However, we found no evidence (e.g. adverse events) that participants experienced problems with muscle soreness during the pilot study. Therefore, we are unclear why dose was modified if no problems were experienced. We could not compare dose in the fourth RCT (Manning *et al.* 2014) owing to the pilot study being unpublished.

We also explored what evidence the pilot studies cited to underpin dose. Neuberger *et al.* (1997) reported their intervention was developed by two physical therapists, an aerobics instructor hired to teach the class and the principal investigator, but did not cite any evidence to underpin dose. Marcora *et al.* (2005) cited the 2002 ACSM position stand: progression models in resistance training for healthy adults [36] to underpin dose sufficient for achieving optimal stimulation of muscle hypertrophy. Manning *et al.* (2015) cited Hurley *et al.* (2007) [72], an RCT-targeting chronic knee pain (ESCAPE programme) for development of their upper-limb intervention (EXTRA programme). The pilot study for Lamb *et al.* (2015) was only described briefly [73, 74] and its dose was underpinned by a variety of evidence sources. Lamb indicated the initial design of the intervention was based on an RCT conducted by O’Brien *et al.* (2006) which also involved the rheumatoid hand and was therefore relevant. The content of the exercise programme used by O’Brien was defined by expert opinion (an unpublished survey of 60 senior hand therapists), one of the lowest levels of evidence [24].

### RCTs using dose escalation methodology

Across the thirty-two trials (Supplementary Table S1) we found no evidence of dose-escalation methodology reported. We also found no evidence of the methodology being used in the four pilot studies identified above (Supplementary Table S4).

### Quality, consistency and applicability of the underpinning evidence

#### Quality

We rated the quality of the twenty-seven underpinning evidence sources using the OCEBM level of evidence framework (Supplementary Table S5). We judged eight (29.6%) to be level 2 evidence [39, 40, 42, 43, 72, 77–79], three (11.1%) to be level 3 evidence [75, 76, 80] and one (3.7%) to be level 5 evidence [81]. Several of the underpinning evidence sources were guidelines, literature reviews or books. Exploring the references these sources used, we assigned a level of evidence rating that best described their quality. We rated five (18.5%) to range between 2–5 [36, 76, 82, 83]. ACSM (2002) [36] was cited twice. The pilot study [76] of Lemmey *et al.* (2009) cited the ACSM position stand [36] to underpin dose. In this instance, we explored the evidence used by the position stand to assign the pilot study a level of evidence rating of 2–5. The remaining two (7.4%), we assigned a rating of 2–4 [84] and 3–5 [85]. Sometimes, assigning a level of evidence wasn’t straightforward. We rated six (22.2%) as ‘unclear’ [86–91]. Two (7.4%) evidence sources we assigned an ‘incorrect’ rating. One source was not relevant to volitional strengthening exercise and focused on electrostimulation [92]. On the second occasion, the source appeared to be incorrectly referenced by the RCT [65] and we were unable to retrieve it for investigation

#### Consistency

We explored whether the RCT used the same dose as described/recommended by the underpinning evidence (Supplementary Table S5). We found twenty-two examples across nine RCTs [48, 50, 52, 54–56, 59, 61, 68] where the dose used was the same as the dose used/recommended by the underpinning evidence source. There were forty-nine examples across eight RCTs where the dose used was different [48, 53, 54, 56, 59, 61, 65, 68]. We found forty-six examples across nine RCTs [48, 52–54, 59–61, 65, 68] where we were unable to compare dose used due to insufficient detail and across six RCTs [48, 50, 55, 56, 60, 65], we found thirty-seven examples where individual dose parameters were unsupported with evidence.

#### Applicability

The applicability of the twenty-seven underpinning evidence sources in relation to RA, gender and age varied (Supplementary Table S5). Fourteen (51.8%) were judged not applicable to RA [36, 72, 78, 81, 82, 84–88, 90, 91], + (Incorrect citiation: ACSM, 2006) seventeen (62.9%) to gender [36, 43, 81–92] + (Incorrect citation: ACSM, 2006) and eighteen (66.6%) to age [36, 42, 78–92]. + (Incorrect citation: ACSM,2006).

#### Relationship between RoB and underpinning evidence

We explored whether we could identify if a relationship existed between the RoB for the twelve RCTs and the judged quality (OCEBM level of evidence) of the underpinning evidence (Supplementary Table S6). Relating to RoB, four (33.3%) were assessed to be at low RoB [53, 54, 61, 68], five (41.6%) were assessed to be at unclear RoB [48, 50, 52, 56, 59] and three (25%) were assessed to be at high RoB [55, 60, 65]. While there were too few studies for reliable statistical evaluation, of the eight RCTs assessed as unclear, or at high risk of bias, seven (87.5%) had evidence rated as incorrect or unclear [50, 52, 55, 56, 59, 60, 65]. Of the four assessed at low RoB, none of the underpinning evidence sources were rated as incorrect or unclear.

## Discussion

This is the first systematic review that we are aware of to explore in detail the underpinning evidence used by healthcare researchers to justify the prescribed dose of strengthening exercise used in clinical trials of RA. We identified that the majority of clinical trials involving exercise in RA do not report the use of evidence to underpin exercise dose. Only four trials formally piloted the intervention and its dose prior to evaluation. None of the pilot studies used dose-escalation designs to optimise dose-response, something which is more commonly seen in the evaluation of new drugs. In the absence of formal piloting, the underpinning evidence cited to justify dose parameters (when used) varied in quality and applicability and sometimes did not support the reported dose parameters.

The lack of formal piloting highlighted by this review suggests that current practice in the field of RCTs using exercise-based interventions in RA does not align with the MRC framework for the development and evaluation of complex interventions. Potential reasons for not piloting may include lack of time, research culture, funding, conflicting priorities, policy focus etc, though in some cases may simply reflect a lack of reporting [94]. For the minority of RCTs who piloted their interventions, dose-escalation methods were not used. In the absence of such methods, we explored how the dose of strengthening exercise was developed. The underpinning evidence sources used by the pilot studies (where published) led us to conclude that development was often based on expert opinion [54, 75] and/or evidence that was not applicable to the clinical population [48, 53, 54]. In the absence of robust empirical data for dose, such approaches to development may be a reasonable attempt in deciding what dose is best to prescribe. However, when researchers have used similar methods to those seen in drugs trials, they discovered discrepancies between the dose that patients could tolerate compared with the dose recommended in the literature. Whilst the number of early phase trials using these methods in exercise is low, these findings suggest that relying on expert opinion and consensus alone may be inaccurate and illustrates the potential value of pilot studies using dose escalation methodology for tailoring dose [95, 96].

In the absence of piloting, the judicious use of evidence to underpin all aspects of dose development should be expected [97], yet only a small proportion of RCTs reported using the evidence. This finding is consistent with insufficient reporting of physiotherapy interventions [98] and complex interventions seen more broadly [99], and is a cause for methodological concern. In many cases, dose parameters were insufficiently described. Only four of the twelve RCTs that reported underpinning evidence had a low overall RoB rating. When exploring if a relationship existed between RoB and the level of underpinning evidence used, we found seven of the eight RCTs with an overall RoB rating of unclear/high also had underpinning evidence that was rated incorrect or unclear. Whilst not enough trials for statistical evaluation, these trials appeared to be using underpinning evidence that was less robust in terms of quality. Overall, our findings indicate the development and testing of exercise dose in clinical trials is an area that should be improved.

### Strengths and limitations of this review

This review offers new insight into clinical trials using exercise interventions. Our comprehensive search strategy without language limitations and methodological approach allowed us to explore in detail the evidence used to underpin dose of strengthening exercise. We acknowledge our review does have some limitations. Firstly, without a complete published description of the intervention, incomplete reporting by both RCTs and the underpinning evidence limited the amount of information available to conduct the review, though this in itself highlights a challenge for this field. Secondly, owing to the number of clinical trials involving strengthening exercise in musculoskeletal conditions, we chose only to include people living with RA. It is possible that the underpinning evidence for exercise dose in other conditions may be more robust. However, given the broad issues identified in the current review, we suggest this is unlikely and may not be limited to strengthening exercise in RA [14]. Thirdly, the novel and exploratory nature of this review meant we could not anticipate all of the challenges for grading the quality of the underpinning evidence. Judging the quality was not always easy in practice. The grading process was more complicated for pilot studies, literature reviews, clinical guidelines and books as it necessitated going back a further generation of evidence. Often with literature reviews, guidelines and books, it was not always clear where in the text support existed. Whilst these types of evidence source may be useful for assimilating large bodies of evidence on a particular topic, using these to underpin dose has potential drawbacks for assessing quality, consistency and applicability. Some RCTs did not stipulate what part of the underpinning evidence they used. This meant that in order to grade quality, sometimes a pragmatic approach was needed by the research team to be able to reach a consensus. Similarly, we judged applicability of the underpinning evidence by looking for areas of homogeneity/heterogeneity across RA, gender and age. These factors were chosen intuitively as we felt busy researchers and clinicians could easily interpret how applicable the underpinning evidence was to clinical population in the RCT. However, we acknowledge that other factors could also be used to assess applicability. Should researchers from other areas wish to use our approach, we hope our transparency in our methods only serves to improve future attempts to understand what evidence is used to underpin such an important part of any intervention.

### Implications

In the context of patient-centred care, the prescription of an effective exercise intervention should be tailored to meet the needs of the individual [11]; for strengthening exercise this should be done within a broader framework that is underpinned by evidence. Our results indicate researchers need to improve not only the standard of reporting related to their interventions, but also the evidence they use to justify their decisions about what dose to prescribe. Reporting guidelines like TIDieR and CERT [8, 30] should be used to raise standards going forward and as these evolve [14], could recommend researchers be explicit with type, quality, consistency and applicability of evidence they have used to support each dose parameter. Funders and peer reviewers should take a careful and critical approach when considering how exercise dose has been formulated. Those interventions that fail to offer evidence supporting dose, or use evidence of low quality and applicability, may not in the future be funded or published. The absence of clear robust evidence supporting dose identified by this review indicates pilot testing using dose escalation methodology may help answer uncertainties about what dose works best [9]. The implications of this would necessitate funders considering more funding and time to support researchers in generating the preliminary data before conducting a definitive RCT.

## Conclusion

Our systematic review identified that the majority of included RCTs did not report pilot studies or evidence to underpin exercise dose. When evidence is cited, the different types used vary in quality, consistency and applicability. Our findings question whether dose is optimised for use with the clinical populations, which is a cause for methodological concern. There are clear scientific imperatives to improve practice in this area of clinical research, including to maximise the potential for exercise interventions to deliver benefit. Addressing these weaknesses may contribute to better quality research being conducted and reducing research waste in exercise interventions.

## Supplementary Material

keaa150_supplementary_data
