# Supplementary material for: A systematic review exploring the evidence reported to underpin exercise dose in clinical trials of rheumatoid arthritis
Source: Rheumatology (Oxford). 2020 Aug 11;59(11):3147–57. doi: 10.1093/rheumatology/keaa150 (PMC7590408; doi:10.1093/rheumatology/keaa150)
Supplement: keaa150_supplementary_data [file keaa150_supplementary_data.zip › Supplementary material S1_GB05082020.docx]

**A systematic review exploring the evidence reported to underpin exercise dose in clinical trials of rheumatoid arthritis**

**Literature search strategies**

MEDLINE Search Strategy

Database and platform: MEDLINE(R) Epub Ahead of Print, In-Process & Other Non-Indexed Citations, Ovid MEDLINE(R) Daily and Ovid MEDLINE(R) 1946 to Present (via OVID)

Search date: 18 May 2018 with update search (covering May 2018 to April 2019) conducted on 3 April 2019.

Search filter: Cochrane Highly Sensitive Search Strategy for identifying randomized trials in MEDLINE: sensitivity-maximizing version (2008 revision); Ovid format.

1. Arthritis, Rheumatoid/

2. (RA or rheumatoid).ti,ab.

3. (rheumatoid adj1 arthritis).ti,ab,kw.

4. or/1-3

5. Exercise/

6. Exercise Therapy/

7. Plyometric Exercise/

8. Exercise Movement Techniques/

9. Physical Therapy Modalities/

10. Physical Fitness/

11. Physical Endurance/

12. (exercis$ adj3 (home or programme$ or program$ or therap$ or technique$ or train$ or treatment$ or intervention$ or supervised)).ti,ab,kw.

13. ((therapeutic or land or intensi$ or dynamic or isometric or isotonic or isokinetic) adj3 (exercis$ or train$)).ti,ab,kw.

14. (physical adj1 (activ$ or education$ or fitness or train$ or therap$ or treatment$ or intervention$)).ti,ab,kw.

15. (physio or physiotherap$).ti,ab,kw.

16. (cycle or cycling or bicycle or walk$).ti,ab,kw.

17. (physical adj1 condition$ adj1 (exercis$ or train$ or programme$ or program$)).ti,ab,kw.

18. ((muscle or grip$) adj2 (programme$ or program$ or therap$ or technique$ or train$ or treatment$ or intervention$ or exercis$)).ti,ab,kw.

19. ((hand$ or wrist$ or shoulder$ or knee$ or ankle$ or joint$ or elbow$ or hip or cervical or lumbar or trunk) adj1 (strength$ or exercis$ or therap$)).ti,ab,kw.

20. Resistance Training/

21. ((resistance or strength$ or weight or endurance) adj1 (programme$ or program$ or therap$ or technique$ or train$ or treatment$ or intervention$ or exercis$)).ti,ab,kw.

22. or/5-21

23. 4 and 22

24. randomized controlled trial.pt.

25. controlled clinical trial.pt.

26. randomized.ab.

27. placebo.ab.

28. drug therapy.fs.

29. randomly.ab.

30. trial.ab.

31. groups.ab.

32. or/24-31

33. 23 and 32

34. exp animals/ not humans.sh.

35. 33 not 34

36. limit 35 to yr="2000-2018"

EMBASE Search Strategy

Database and platform: Embase 1974 to 2018 Week 20 (via OVID)

Search date: 18 May 2018 with update search (covering May 2018 to April 2019) conducted on 3 April 2019.

Search filter: McMaster EMBASE RCT search filter (Best balance of sensitivity and specificity)

1. Rheumatoid Arthritis/

2. (RA or rheumatoid).ti,ab.

3. (rheumatoid adj1 arthritis).ti,ab,kw.

4. or/1-3

5. Exercise/

6. Dynamic Exercise/

7. Endurance Training/

8. Exercise Intensity/

9. Isokinetic Exercise/

10. Isometric Exercise/

11. Isotonic Exercise/

12. Muscle Exercise/

13. Kinesiotherapy/

14. Plyometrics/

15. Fitness/

16. Physical Activity/

17. Grip Strength/

18. Muscle Strength/

19. Physical Capacity/

20. Muscle Training/

21. Hand Grip/

22. Pinch Strength/

23. Hand Strength/

24. Isometrics/

25. (exercis$ adj3 (home or programme$ or program$ or therap$ or technique$ or train$ or treatment$ or intervention$ or supervised)).ti,ab,kw.

26. ((therapeutic or land or intensi$ or dynamic or isometric or isotonic or isokinetic) adj3 (exercis$ or train$)).ti,ab,kw.

27. (physical adj1 (activ$ or education$ or fitness or train$ or therap$ or treatment$ or intervention$)).ti,ab,kw.

28. (physio or physiotherap$).ti,ab,kw.

29. (cycle or cycling or bicycle or walk$).ti,ab,kw.

30. (physical adj1 condition$ adj1 (exercis$ or train$ or programme$ or program$)).ti,ab,kw.

31. ((muscle or grip$) adj2 (programme$ or program$ or therap$ or technique$ or train$ or treatment$ or intervention$ or exercis$)).ti,ab,kw.

32. ((hand$ or wrist$ or shoulder$ or knee$ or ankle$ or joint$ or elbow$ or hip or cervical or lumbar or trunk) adj1 (strength$ or exercis$ or therap$)).ti,ab,kw.

33. Resistance Training/

34. ((resistance or strength$ or weight or endurance) adj1 (programme$ or program$ or therap$ or technique$ or train$ or treatment$ or intervention$ or exercis$)).ti,ab,kw.

35. or/5-34

36. 4 and 35

37. random:.tw.

38. placebo:.mp.

39. double-blind:.tw.

40. or/37-39

41. 36 and 40

42. limit 41 to yr="2000-2018"

CINAHL Search Strategy

Database and platform: CINAHL (via EbscoHost)

Search date: 18 May 2018 with update search (covering May 2018 to April 2019) conducted on 3 April 2019.

Search filter used: SIGN Search Filter for identifying randomised trials in CINAHL for EBSCO (created by Mark Clowes).

1. (MH "Arthritis, Rheumatoid")

2. (TI "RA" or "rheumatoid") OR (AB "RA" or "rheumatoid")

3. (TI (rheumatoid N1 arthritis)) OR (AB (rheumatoid N1 arthritis))

4. S1 OR S2 OR S3

5. (MH "Exercise")

6. (MH "Therapeutic Exercise")

7. (MH "Plyometrics")

8. (MH "Physical Therapy")

9. (MH "Physical Fitness")

10. (MH "Physical Endurance")

11. (TI (exercis* N3 (home or programme* or program* or therap* or technique* or train* or treatment* or intervention* or supervised)) OR (AB (exercis* N3 (home or programme* or program* or therap* or technique* or train* or treatment* or intervention* or supervised))

12. (TI (therapeutic or land or intensi* or dynamic or isometric or isotonic or isokinetic) N3 (exercis* or train*)) OR (AB (therapeutic or land or intensi* or dynamic or isometric or isotonic or isokinetic) N3 (exercis* or train*))

13. (TI (physical N1 (activ* or education* or fitness or train* or therap* or treatment* or intervention*)) OR (AB (physical N1 (activ* or education* or fitness or train* or therap* or treatment* or intervention*))

14. (TI "physio" or "physiotherap*") OR (AB "physio" or "physiotherap*")

15. (TI "cycle" or "cycling" or "bicycle" or "walk*") OR (AB "cycle" or "cycling" or "bicycle" or "walk*")

16. (TI (physical N1 condition* N1 (exercis* or train* or programme* or program*)) OR (AB (physical N1 condition* N1 (exercis* or train* or programme* or program*))

17. (TI (muscle or grip*) N2 (programme* or program* or therap* or technique* or train* or treatment* or intervention* or exercis*)) OR (AB (muscle or grip*) N2 (programme* or program* or therap* or technique* or train* or treatment* or intervention* or exercis*))

18. (TI (hand* or wrist* or shoulder* or knee* or ankle* or joint* or elbow* or hip or cervical or lumbar or trunk) N1 (strength* or exercis* or therap*)) OR (AB (hand* or wrist* or shoulder* or knee* or ankle* or joint* or elbow* or hip or cervical or lumbar or trunk) N1 (strength* or exercis* or therap*))

19. (MH "Resistance Training")

20. (MH "Muscle Strengthening")

21. (MH "Grip Strength")

22. (MH "Muscle Strength")

23. (MH "Athletic Training Programs")

24. (TI (resistance or strength* or weight or endurance) N1 (programme* or program* or therap* or technique* or train* or treatment* or intervention* or exercis*)) OR (AB (resistance or strength* or weight or endurance) N1 (programme* or program* or therap* or technique* or train* or treatment* or intervention* or exercis*))

25. S5 OR S6 OR S7 OR S8 OR S9 OR S10 OR S11 OR S12 OR S13 OR S14 OR S15 OR S16 OR S17 OR S18 OR S19 OR S20 OR S21 OR S22 OR S23 OR S24

26. S4 AND S25

27. (MH "Clinical Trials+")

28. PT Clinical trial

29. TX clinic* n1 trial*

30. TX ( (singl* n1 blind*) or (singl* n1 mask*) ) or TX ( (doubl* n1 blind*) or (doubl* n1 mask*) ) or TX ( (tripl* n1 blind*) or (tripl* n1 mask*) ) or TX ( (trebl* n1 blind*) or (trebl* n1 mask*) )

31. TX randomi* control* trial*

32. (MH "Random Assignment")

33. TX random* allocat*

34. TX placebo*

35. (MH "Placebos")

36. (MH "Quantitative Studies")

37. TX allocat* random*

38. S27 or S28 or S29 or S30 or S31 or S32 or S33 OR S34 OR S35 OR S36 OR S37

39. S26 and S38

40. PY 2000-2018

41. S39 and S40

AMED Search Strategy

Database and platform: AMED (Allied and Complementary Medicine) 1985 to May 2018 (via OVID)

Search date: 18 May 2018 with update search (covering 2018 to 2019) conducted on 3 April 2019.

1. Arthritis Rheumatoid/

2. (RA or rheumatoid).ti,ab.

3. (rheumatoid adj1 arthritis).ti,ab.

4. 1 or 2 or 3

5. Exercise/

6. Exercise Therapy/

7. Exercise Movement Techniques/

8. Physical Therapy Modalities/

9. Physical Fitness/

10. Physical Endurance/

11. Exercise Tolerance/

12. Rehabilitation/

13. (exercis$ adj3 (home or programme$ or program$ or therap$ or technique$ or train$ or treatment$ or intervention$ or supervised)).ti,ab.

14. ((therapeutic or land or intensi$ or dynamic or isometric or isotonic or isokinetic) adj3 (exercis$ or train$)).ti,ab.

15. (physical adj1 (activ$ or education$ or fitness or train$ or therap$ or treatment$ or intervention$)).ti,ab.

16. (physio or physiotherap$).ti,ab.

17. (cycle or cycling or bicycle or walk$).ti,ab.

18. (physical adj1 condition$ adj1 (exercis$ or train$ or programme$ or program$)).ti,ab.

19. ((muscle or grip$) adj2 (programme$ or program$ or therap$ or technique$ or train$ or treatment$ or intervention$ or exercis$)).ti,ab.

20. ((hand$ or wrist$ or shoulder$ or knee$ or ankle$ or joint$ or elbow$ or hip or cervical or lumbar or trunk) adj1 (strength$ or exercis$ or therap$)).ti,ab.

21. Hand Strength/

22. Resistance Training/

23. ((resistance or strength$ or weight or endurance) adj1 (programme$ or program$ or therap$ or technique$ or train$ or treatment$ or intervention$ or exercis$)).ti,ab.

24. 5 or 6 or 7 or 8 or 9 or 10 or 11 or 12 or 13 or 14 or 15 or 16 or 17 or 18 or 19 or 20 or 21 or 22 or 23

25. 4 and 24

26. limit 25 to yr="2000-2018"

CENTRAL Search Strategy

Database and platform: CENTRAL (via http://cochranelibrary-wiley.com/cochranelibrary/)

Search date: 18 May 2018 with update search (covering 2018 to 2019) conducted on 3 April 2019.

1. [mh "Arthritis, Rheumatoid"] in Trials

2. (RA or rheumatoid):ti,ab,kw in Trials

3. (rheumatoid next/1 arthritis):ti,ab,kw in Trials

4. #1 or #2 or #3

5. [mh "Exercise"] in Trials

6. [mh "Exercise Therapy"] in Trials

7. [mh "Plyometric Exercise"] in Trials

8. [mh "Exercise Movement Techniques"] in Trials

9. [mh "Physical Therapy Modalities"] in Trials

10. [mh "Physical Fitness"] in Trials

11. [mh "Physical Endurance"] in Trials

12. (exercis* next/3 (home or programme* or program* or therap* or technique* or train* or treatment* or intervention* or supervised)):ti,ab,kw in Trials

13. ((therapeutic or land or intensi* or dynamic or isometric or isotonic or isokinetic) next/3 (exercis* or train*)):ti,ab,kw in Trials

14. (physical next/1 (activ* or education* or fitness or train* or therap* or treatment* or intervention*)):ti,ab,kw in Trials

15. (physio or physiotherap*):ti,ab,kw in Trials

16. (cycle or cycling or bicycle or walk*):ti,ab,kw in Trials

17. (physical next/1 condition* next/1 (exercis* or train* or programme* or program*)):ti,ab,kw in Trials

18. ((muscle or grip*) next/2 (programme* or program* or therap* or technique* or train* or treatment* or intervention* or exercis*)):ti,ab,kw in Trials

19. [mh "Hand Strength"] in Trials

20. ((hand* or wrist* or shoulder* or knee* or ankle* or joint* or elbow* or hip or cervical or lumbar or trunk) next/1 (strength* or exercis* or therap*)):ti,ab,kw in Trials

21. [mh "Resistance Training"] in Trials

22. ((resistance or strength* or weight or endurance) next/1 (programme* or program* or therap* or technique* or train* or treatment* or intervention* or exercis*)):ti,ab,kw in Trials

23. #5 or #6 or #7 or #8 or #9 or #10 or #11 or #12 or #13 or #14 or #15 or #16 or #17 or #18 or #19 or #20 or #21 or #22

24. #4 and #23

25. #24 Publication Year from 2000 to 2018

PEDro Search Strategy

Database and platform: PEDro (http://search.pedro.org.au/advanced-search)

Search date: 18 May 2018 with update search (covering 2018 to 2019) conducted on 3 April 2019.

SEARCH STRATEGY 1

1. Abstract & Title: Rheumatoid Arthritis

AND

2. Therapy: fitness training

AND

3. Method: clinical trial

AND

4. Published Since: 2000

OR

SEARCH STRATEGY 2

1. Abstract & Title: Rheumatoid Arthritis

AND

2. Therapy: strength training

AND

3. Method: clinical trial

AND

4. Published Since: 2000

OR

SEARCH STRATEGY 3

1. Abstract & Title: Rheumatoid Arthritis Exercis*

AND

2. Method: Clinical trial

AND

3. Published since: 2000
