# Supplementary material for: A systematic review exploring the evidence reported to underpin exercise dose in clinical trials of rheumatoid arthritis
Source: Rheumatology (Oxford). 2020 Aug 11;59(11):3147–57. doi: 10.1093/rheumatology/keaa150 (PMC7590408; doi:10.1093/rheumatology/keaa150)
Supplement: keaa150_supplementary_data [file keaa150_supplementary_data.zip › Supplementary material S2_GB05082020.pdf]

Neuberger et al (2007)

Slides 1-5

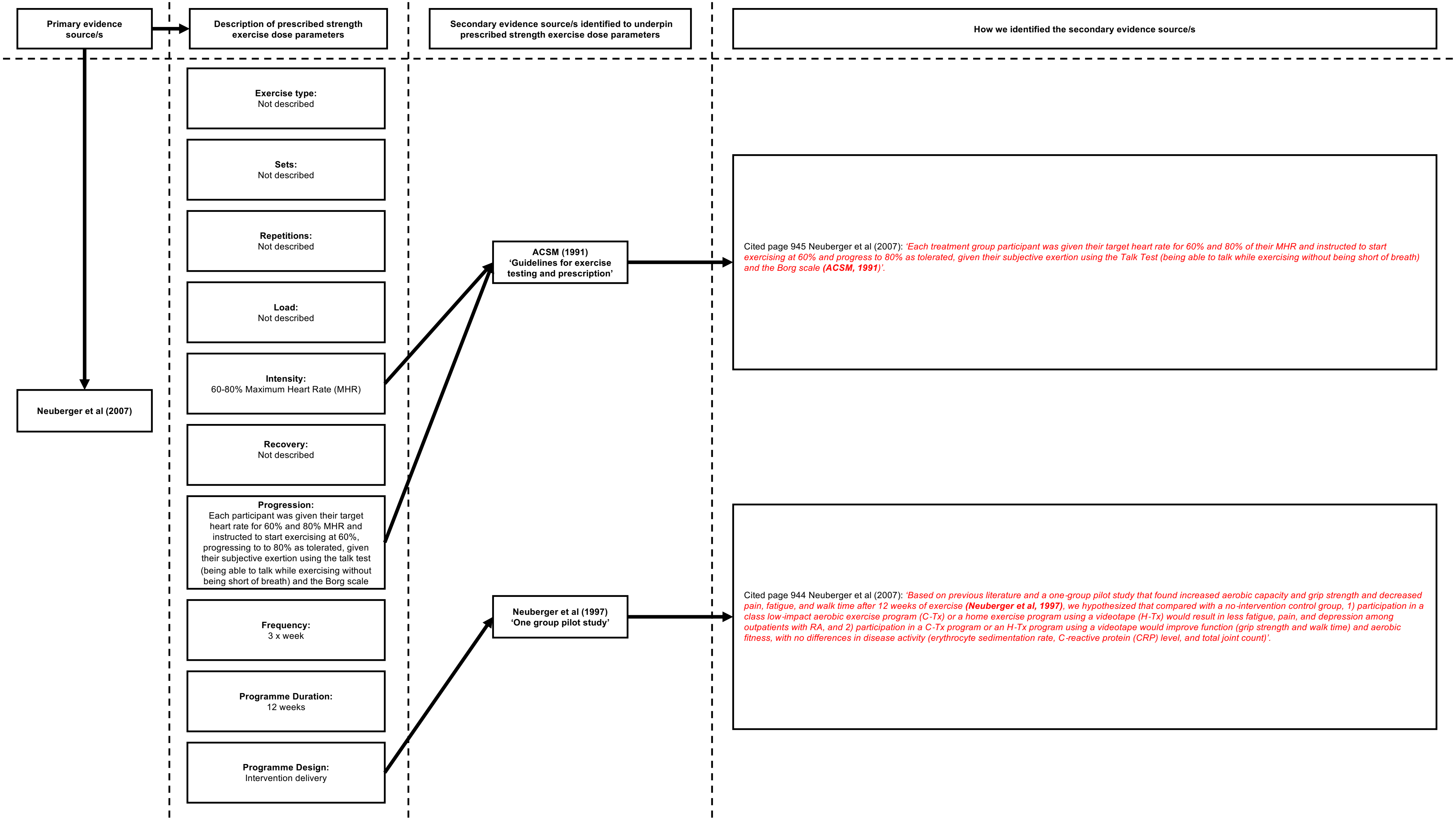
Primary evidence source/s
Description of prescribed strength exercise dose parameters
Secondary evidence source/s identified to underpin prescribed strength exercise dose parameters
How we identified the secondary evidence source/s
Neuberger et al (2007)
Exercise type: Not described
Sets: Not described
Repetitions: Not described
Load: Not described
Intensity: 60-80% Maximum Heart Rate (MHR)
Recovery: Not described
Progression: Each participant was given their target heart rate for 60% and 80% MHR and instructed to start exercising at 60%, progressing to to 80% as tolerated, given their subjective exertion using the talk test (being able to talk while exercising without being short of breath) and the Borg scale
Frequency: 3 x week
Programme Duration: 12 weeks
Programme Design: Intervention delivery
ACSM (1991) 'Guidelines for exercise testing and prescription'
Neuberger et al (1997) 'One group pilot study'
Cited page 945 Neuberger et al (2007): 'Each treatment group participant was given their target heart rate for 60% and 80% of their MHR and instructed to start exercising at 60% and progress to 80% as tolerated, given their subjective exertion using the Talk Test (being able to talk while exercising without being short of breath) and the Borg scale (ACSM, 1991)'.
Cited page 944 Neuberger et al (2007): 'Based on previous literature and a one-group pilot study that found increased aerobic capacity and grip strength and decreased pain, fatigue, and walk time after 12 weeks of exercise (Neuberger et al, 1997), we hypothesized that compared with a no-intervention control group, 1) participation in a class low-impact aerobic exercise program (C-Tx) or a home exercise program using a videotape (H-Tx) would result in less fatigue, pain, and depression among outpatients with RA, and 2) participation in a C-Tx program or an H-Tx program using a videotape would improve function (grip strength and walk time) and aerobic fitness, with no differences in disease activity (erythrocyte sedimentation rate, C-reactive protein (CRP) level, and total joint count)'.

Secondary evidence source/s identified to underpin prescribed strength exercise dose parameters

Oxford centre for evidence based medicine 2011 levels of evidence (OCEBM)

How we rated OCEBM level of evidence for the secondary evidence source/s

ACSM (1991)
'Guidelines for exercise testing and prescription

→ **OCEBM level**
'Unclear'

→ **Key information:**

- ACSM (1991) was identified as a guideline. Therefore, we attempted to identify tertiary evidence source/s most appropriate to underpinning the identified dose parameter/s it was used to support. In view that Neuberger et al (2007) used ACSM (1991) to underpin both intensity and method of progression, we searched ACSM (1991) for tertiary evidence supporting these parameters.

Parameter: Intensity

- Pages 99-100 from chapter 5 (ACSM, 1991) describe exercise prescription by heart rate in the same range (60-80%MHR) as prescribed by the intervention from the primary evidence source. Chapter 5 was underpinned by 17 citations made up of 11 books, 2 ACSM position stands, 2 cohort studies, 1 RCT and 1 non-systematic review.

Parameter: Progression

- Page 182 from chapter 7 (ACSM, 1991) describes the 'talk-test', a method for preventing over-exertion. The text discussing this method was located under a chapter sub-heading 'Special conditions within the normal population – pregnancy'. Chapter 7 was underpinned by 29 citations made up of 9 non-systematic reviews, 6 books, 3 book chapters, 3 guidelines, 2 non-RCTs, 2 cohort studies, 1 RCT, 1 matched case-control study, 1 symposium, 1 opinion statement.
- Pages 101-102 from chapter 5 (ACSM, 1991) describe exercise prescription by rating of perceived exertion (RPE). We were unable to cross-reference this with the primary evidence source because the intervention provided no description about the the Borg scale was used.

We rated OCEBM level of evidence for this source as 'unclear' because:

- The secondary evidence did not directly link its text to the tertiary evidence sources contained in the chapter references.

Neuberger et al (1997)
'One group pilot study'

→ **OCEBM Level**
'3'

→ **Key information:**

- Owing to Neuberger et al (1997) being identified as a pilot study, we attempted to identify tertiary evidence source/s underpinning the dose parameter/s it was used to support. In view that Neuberger et al (2007) used Neuberger et al (1997) to underpin programme design, we searched Neuberger et al (1997) for tertiary evidence supporting this parameter.

Parameter: Programme design

- Page 199 (Neuberger et al, 1997) describes the *'The exercise intervention consisted of participation in a low-impact aerobic exercise class for 1 hr 3 times weekly for 12 weeks. The exercise regimen was designed by two physical therapists, an aerobic instructor hired to teach the class, and the principal investigator. The class consisted of four phases: warm-up exercises, strengthening exercises, low-impact aerobic exercises, and cool-down exercises. Initially, warm-up and strengthening phases were longer in order to build muscle strength. As aerobic minutes were increased, minutes of warmup and cool-down were reduced and documentation of this progression of minutes was recorded. The exercise class was held in a room in the exercise facility on the medical center campus. Subjects came to either an early or a late afternoon class'.*
- We were unable to identify tertiary evidence underpinning the strength exercise prescription parameters used in the pilot study.

We rated this secondary evidence source level 3 (Non-Randomised controlled cohort/follow-up study) because:

- Neuberger et al (1997) is a one group pilot study was a non-randomised controlled cohort study.

Dose parameter<br>Underpinning evidence	Type of strength exercise	Sets	Repetitions	Load	Intensity	Recovery	Method of progression	Frequency	Programme duration	Consistency rating
**Neuberger et al (2007)**	Insufficiently described	Insufficiently described	Insufficiently described	Insufficiently described	60-80% MHR	Insufficiently described	Each participant was given their target HR for 60% & 80% MHR and instructed to start exercising at 60% and progress to 80% as tolerated given their subjective exertion using the Talk test (being able to talk while exercising without being short of breath) and the Borg scale.	3 x week	12 weeks	Exercise type:<br>n/a<br>Sets:<br>n/a<br>Repetitions:<br>n/a<br>Load:<br>n/a<br>Intensity:<br>Consistent<br>Recovery:<br>n/a<br>Progression:<br>Unclear<br>Frequency:<br>n/a<br>Duration:<br>n/a
**ACSM (1991)**	Citation not used to support parameter	Citation not used to support parameter	Citation not used to support parameter	Citation not used to support parameter	60-80% MHR	Citation not used to support parameter	60-80% MHR:<br>Unclear<br>Talk test:<br>Consistent<br>Borg scale:<br>Unclear	Citation not used to support parameter	Citation not used to support parameter	

RCT<br>Underpinning evidence	Type of strength exercise	Sets	Repetitions	Load	Intensity	Recovery	Method of progression	Frequency	Programme duration	Consistency rating
**Neuberger et al (2007)**	Insufficiently described	Insufficiently described	Insufficiently described	Insufficiently described	60-80%1RM	Insufficiently described	Each participant was given their target HR for 60% & 80% MHR and instructed to start exercising at 60% and progress to 80% as tolerated given their subjective exertion using the Talk Test (being able to talk while exercising without being short of breath) and the Borg scale.	3 x week	12 weeks	Exercise type: Unclear<br>Sets: Unclear<br>Repetitions: Unclear<br>Load: Unclear<br>Intensity: Unclear<br>Recovery: Unclear<br>Progression: Inconsistent<br>Frequency: Consistent<br>Duration: Consistent
**Neuberger et al (1997)**	Insufficiently described	Insufficiently described	Insufficiently described	Insufficiently described	Insufficiently described	Insufficiently described	Initially warm-up and strengthening phases were longer in order to build muscle strength. As aerobic minutes were increased, minutes of warm-up and cool-down were reduced.	3 x week	12 weeks	

Flint-Wagner et al (2009)

Slides 6-10

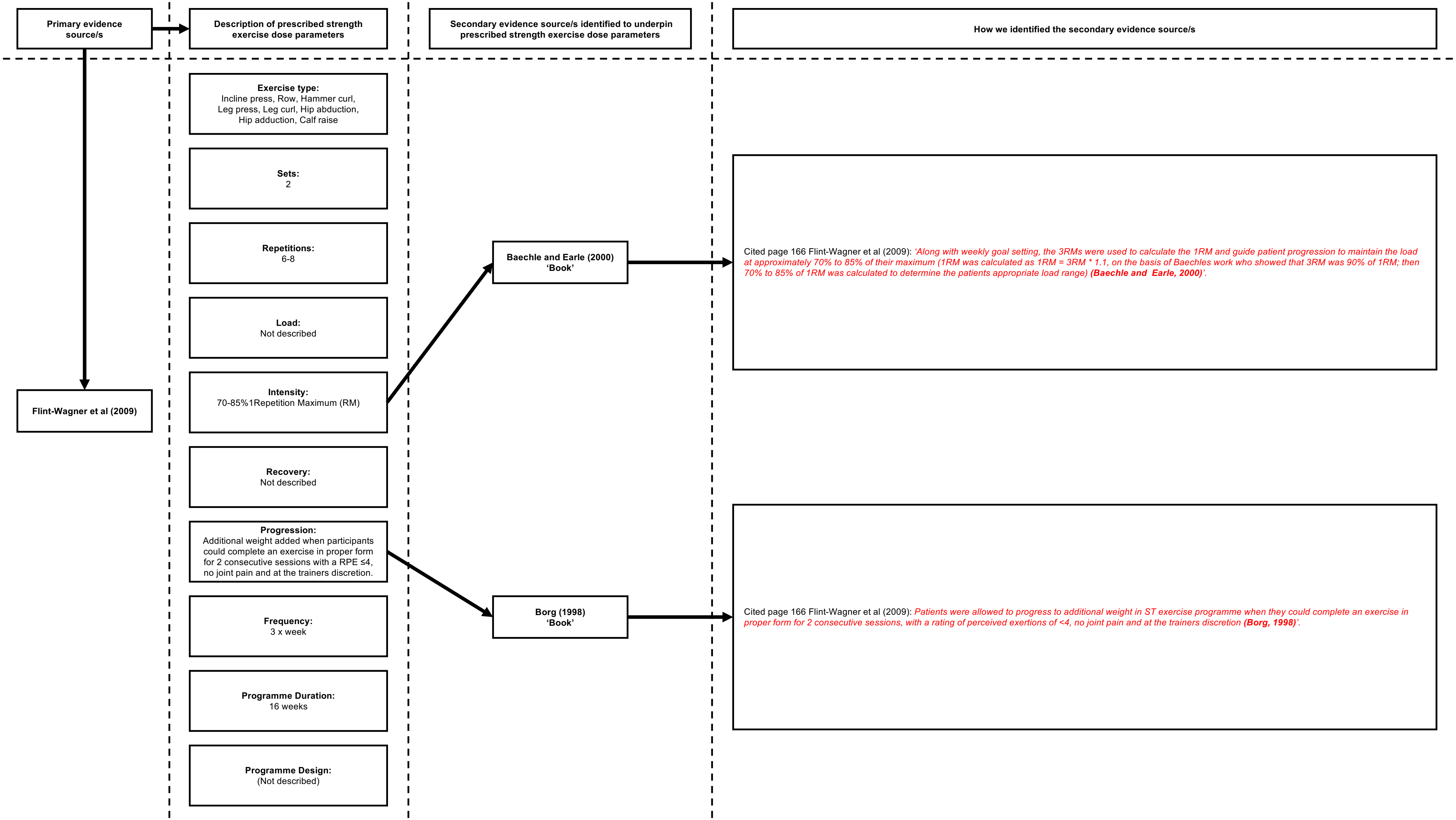
Primary evidence source/s
Description of prescribed strength exercise dose parameters
Secondary evidence source/s identified to underpin prescribed strength exercise dose parameters
How we identified the secondary evidence source/s
Flint-Wagner et al (2009)
Exercise type: Incline press, Row, Hammer curl, Leg press, Leg curl, Hip abduction, Hip adduction, Calf raise
Sets: 2
Repetitions: 6-8
Load: Not described
Intensity: 70-85%1Repetition Maximum (RM)
Recovery: Not described
Progression: Additional weight added when participants could complete an exercise in proper form for 2 consecutive sessions with a RPE ≤4, no joint pain and at the trainers discretion.
Frequency: 3 x week
Programme Duration: 16 weeks
Programme Design: (Not described)
Baechle and Earle (2000) 'Book'
Borg (1998) 'Book'
Cited page 166 Flint-Wagner et al (2009): 'Along with weekly goal setting, the 3RMs were used to calculate the 1RM and guide patient progression to maintain the load at approximately 70% to 85% of their maximum (1RM was calculated as 1RM = 3RM * 1.1, on the basis of Baechles work who showed that 3RM was 90% of 1RM; then 70% to 85% of 1RM was calculated to determine the patients appropriate load range) (Baechle and Earle, 2000)'.
Cited page 166 Flint-Wagner et al (2009): Patients were allowed to progress to additional weight in ST exercise programme when they could complete an exercise in proper form for 2 consecutive sessions, with a rating of perceived exertions of <4, no joint pain and at the trainers discretion (Borg, 1998)'.

Secondary evidence source/s identified to underpin prescribed strength exercise dose parameters

Oxford centre for evidence based medicine 2011 levels of evidence (OCEBM)

How we rated OCEBM level of evidence for the secondary evidence source/s

---

Baechle and Earle (2000) 'Book' → **OCEBM level '3-5'** →

Key information:

- Baechle and Earle (2000) was not identified as pilot study for the primary evidence source, literature review or guideline. However, owing to Baechle and Earle (2000) being a book, we attempted to identify tertiary evidence source/s most appropriate to underpinning the identified prescription parameter/s it was used to support. In view that Flint-Wagner et al (2009) used Baechle and Earle (2000) to underpin intensity, we searched Baechle and Earle (2000) for tertiary evidence supporting this parameter.

Parameter: Intensity

- Pages 395-425 from chapter 18 (Baechle and Earle, 2000) describe resistance training and pages 406-414 focus on describing the relationship between load and repetitions. We were unable to specifically locate where the secondary evidence source describes 3RM being 90% of 1RM, however, we did identify tables 18.7 and 18.8 located on pages 407 and 410-411 respectively, indicating 3 repetitions being equivalent to 93%1RM. 8 citations underpinned tables and were made up of 3 cohort studies, 2 books, 2 charts describing poundage and maximum based on reps and one clinical opinion.

We rated OCEBM level of evidence for this source as '3-5' (Non-Randomised controlled cohort/follow-up study and mechanism-based reasoning) because:

- The tertiary evidence sources identified as most likely underpinning the parameter ranged between these two levels of evidence.

Borg (1998) 'Book' → **OCEBM level 'Unclear'** →

Key information:

- Borg (1998) was not identified as pilot study for the primary evidence source, literature review or guideline. However, owing to Borg (1998) being a book, we attempted to identify tertiary evidence source/s most appropriate to underpinning the identified prescription parameter/s it was used to support. In view that Flint-Wagner et al (2009) used Borg (1998) to underpin progression, we searched Borg (1998) for tertiary evidence supporting this parameter.

Parameter: Progression

- ❑ Borg (1998) was used to underpin progression based on the participant being able to perform the exercise with 1) Proper form for 2 consecutive sessions, 2) Perform the exercise with a rating of perceived exertion (RPE) ≤4, 3) No joint pain and 4) At the trainers discretion. Owing to the secondary evidence source being a book discussing Borg's perceived exertion and pain scales, we cautiously assume the secondary evidence source was used to support progression based on a participants RPE being ≤4. Chapter 11 describes applying the scales to training and rehabilitation. Pages 78-79 from chapter 11 describe short-term exercise and muscular training. Figure 11.2 located on page 79 describes using perception for force and fatigue based on the 1st repetition and last repetition. We cautiously assume that if the participant is able to perform 2 sets of 6-8 repetitions and rate the last repetition ≤4, they could be considered for progression, if they also performed the exercise with proper form, had no joint pain and the trainer was satisfied. Figure 11.2 did not provide a direct link to a underpinning tertiary evidence source/s.

We rated OCEBM level of evidence for this source as 'unclear' because:

- The secondary evidence did not directly link its text to the tertiary evidence sources contained in the references.

Dose parameter<br>Underpinning evidence	Type of strength exercise	Sets	Repetitions	Load	Intensity	Recovery	Method of progression	Frequency	Programme duration	Consistency rating
**Flint-Wagner et al (2009)**	1. Incline press<br>2. Row<br>3. Hammer curl<br>4. Leg press<br>5. Leg curl<br>6. Hip abduction<br>7. Hip adduction<br>8. Calf raise	2 sets	6-8 repetitions	Insufficiently described	70-85% 1Repetition Maximum (RM)	Insufficiently described	Additional weight added when participants could complete an exercise in proper form for 2 consecutive sessions with a RPE ≤4, no joint pain and at the trainers discretion. Along with weekly goal setting, the 3RMs were used to calculate the 1RM and guide patient progression to maintain load at approximately 70% to 85% of their maximum (1RM was calculated as 1RM – 3RM * 1.1 on the basis of Baechle's work showed that 3RM was 90% of 1RM; then 70% to 85% of 1RM was calculated to determine the patients appropriate load range.	3 x week	16 weeks	Exercise type:<br>n/a<br><br>Sets:<br>n/a<br><br>Repetitions:<br>n/a<br><br>Load:<br>n/a<br><br>Intensity:<br>Unclear<br><br>Recovery:<br>n/a<br><br>Progression:<br>n/a<br><br>Frequency:<br>n/a<br><br>Duration:<br>n/a
**Baechle and Earle (2000)**	Citation not used to support parameter	Citation not used to support parameter	Citation not used to support parameter	Citation not used to support parameter	Unclear	Citation not used to support parameter	Citation not used to support parameter	Citation not used to support parameter	Citation not used to support parameter	

RCT<br>Underpinning evidence	Type of strength exercise	Sets	Repetitions	Load	Intensity	Recovery	Method of progression	Frequency	Programme duration	Consistency rating
**Flint-Wagner et al (2009)**	1. Incline press<br>2. Row<br>3. Hammer curl<br>4. Leg press<br>5. Leg curl<br>6. Hip abduction<br>7. Hip adduction<br>8. Calf raise	2 sets	6-8 repetitions	Insufficiently described	70-85% 1Repetition Maximum (RM)	Insufficiently described	Additional weight added when participants could complete an exercise in proper form for 2 consecutive sessions with a RPE ≤4, no joint pain and at the trainers discretion. Along with weekly goal setting, the 3RMs were used to calculate the 1RM and guide patient progression to maintain load at approximately 70% to 85% of their maximum (1RM was calculated as 1RM – 3RM * 1.1 on the basis of Baechle's work showed that 3RM was 90% of 1RM; then 70% to 85% of 1RM was calculated to determine the patients appropriate load range.	3 x week	16 weeks	Exercise type: n/a<br>Sets: n/a<br>Repetitions: n/a<br>Load: n/a<br>Intensity: Unclear<br>Recovery: n/a<br>Progression: Unclear<br>Frequency: n/a<br>Duration: n/a
**Borg (1998)**	Citation not used to support parameter	Citation not used to support parameter	Citation not used to support parameter	Citation not used to support parameter	Citation not used to support parameter	Citation not used to support parameter	Unclear	Citation not used to support parameter	Citation not used to support parameter	

Lemmey et al (2009)

Slides 11-15

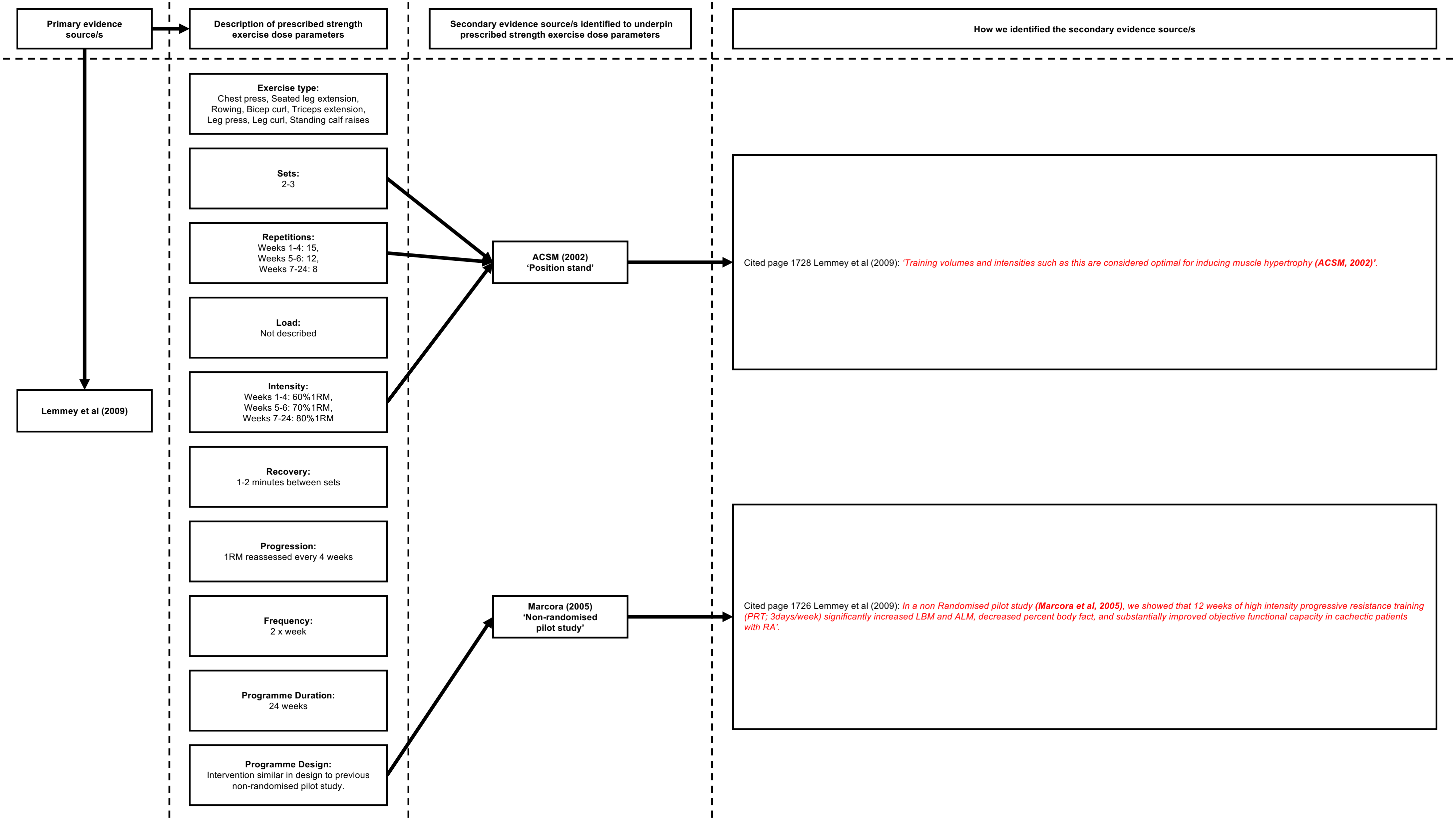
Primary evidence source/s
Description of prescribed strength exercise dose parameters
Secondary evidence source/s identified to underpin prescribed strength exercise dose parameters
How we identified the secondary evidence source/s
Lemmey et al (2009)
Exercise type: Chest press, Seated leg extension, Rowing, Bicep curl, Triceps extension, Leg press, Leg curl, Standing calf raises
Sets: 2-3
Repetitions: Weeks 1-4: 15, Weeks 5-6: 12, Weeks 7-24: 8
Load: Not described
Intensity: Weeks 1-4: 60%1RM, Weeks 5-6: 70%1RM, Weeks 7-24: 80%1RM
Recovery: 1-2 minutes between sets
Progression: 1RM reassessed every 4 weeks
Frequency: 2 x week
Programme Duration: 24 weeks
Programme Design: Intervention similar in design to previous non-randomised pilot study.
ACSM (2002) 'Position stand'
Marcora (2005) 'Non-randomised pilot study'
Cited page 1728 Lemmey et al (2009): *'Training volumes and intensities such as this are considered optimal for inducing muscle hypertrophy* ***(ACSM, 2002)'****.*
Cited page 1726 Lemmey et al (2009): *In a non Randomised pilot study* ***(Marcora et al, 2005)****, we showed that 12 weeks of high intensity progressive resistance training (PRT; 3days/week) significantly increased LBM and ALM, decreased percent body fact, and substantially improved objective functional capacity in cachectic patients with RA'.*

Secondary evidence source/s identified to underpin prescribed strength exercise dose parameters

Oxford centre for evidence based medicine 2011 levels of evidence (OCEBM)

How we rated OCEBM level of evidence for the secondary evidence source/s

---

ACSM (2002) 'Position stand → **OCEBM level '2-5'** →

Key information:

- Owing to ACSM (2002) being identified as a guideline, we attempted to identify tertiary evidence source/s most appropriate to underpinning the identified prescription parameter/s it was used to support. In view that Lemmey et al (2009) used ACSM (2002) to underpin volume and intensity optimal for muscle hypertrophy we searched ACSM (2002) for tertiary evidence supporting these parameters.

Parameter: Volume and Intensity

- We refined our search by focusing on evidence underpinning muscle hypertrophy owing to Lemmey et al (2009) describing this when citing ACSM (2002). We located a section titled 'Programme design recommendations for increasing muscle hypertrophy' and cautiously identified 19 tertiary evidence sources underpinning the approach considered optimal for inducing muscle hypertrophy. We identified 10 cohort studies, 4 non-randomised controlled trials, 3 RCTs, 1 article with describing a mix of RCTs and cohort studies and 1 book.

We rated OCEBM level of evidence for this source as '2-5' (Randomised trial or observational study with dramatic effect to mechanism-based reasoning) because:

- The tertiary evidence underpinning training volumes and intensities considered optimal for inducing muscle hypertrophy ranged between these two levels of evidence.

Marcora (2005) 'Non-randomised pilot study' → **OCEBM level '2-5'** →

Key information:

- Owing to Marcora et al (2005) being identified as the pilot study, we attempted to identify tertiary evidence source/s most appropriate to underpinning the identified prescription parameter/s it was used to support.. In view that Lemmey et al (2009) used Marcora et al (2005) to underpin the progressive resistance training (PRT) approach used, we searched Marcora et al (2005) for tertiary evidence supporting this parameter. We identified 1 tertiary evidence source/s underpinning prescription parameter/s it was used to support.

Parameter: Programme design (Progressive resistance training)

- Page 1032 Marcora et al (2005) describes: *'Therefore, we designed the present controlled study to investigate whether a PRT program prescribed for optimal stimulation of muscle hypertrophy* ***(ACSM, 2002)*** *is feasible and tolerable in mildly disabled patients with well-controlled RA, and to provide preliminary evidence for its efficacy as adjunct treatment of cachexia in this population'.* We identified 10 cohort studies, 4 non-randomised controlled trials, 3 RCTs, 1 article with describing a mix of RCTs and cohort studies and 1 book.

We rated OCEBM level of evidence for this source as '2-5' (Randomised trial or observational study with dramatic effect to mechanism-based reasoning) because:

- Marcora et al (2005) is a non-randomised 2-group, matched, parallel, controlled, pretest-posttest study
- The tertiary evidence underpinning training volumes and intensities considered optimal for inducing muscle hypertrophy ranged between these two levels of evidence.

Dose parameter<br>Underpinning evidence	Type of strength exercise	Sets	Repetitions	Load	Intensity	Recovery	Method of progression	Frequency	Programme duration	Consistency rating
**Lemmey et al (2009)**	1. Chest press,<br>2. Seated leg extension,<br>3. Rowing,<br>4. Bicep curl,<br>5. Triceps extension,<br>6. Leg press,<br>7. Leg curl,<br>8. Standing calf raises	2-3 sets	Weeks 1-4:<br>15 repetitions<br><br>Weeks 5-6:<br>12 repetitions<br><br>Weeks 7-24:<br>8 repetitions	Insufficiently described	Weeks 1-4:<br>60%1RM<br><br>Weeks 5-6:<br>70%1RM<br><br>Weeks 7-24:<br>80%1RM	1-2 minutes between sets	1RM reassessed every 4 weeks	2 x week	24 weeks	Exercise type:<br>n/a<br><br>Sets:<br>Consistent<br><br>Repetitions:<br>Inconsistent<br><br>Load:<br>n/a<br><br>Intensity:<br>Inconsistent<br><br>Recovery:<br>n/a<br><br>Progression:<br>Unclear<br><br>Frequency:<br>n/a<br><br>Duration:<br>n/a
**ACSM (2002)**	Citation not used to support parameter	1-3 sets for novice or intermediate individuals	8-12 repetitions for novice or intermediate individuals	Citation not used to support parameter	70-85%1RM for novice or intermediate individuals	Citation not used to support parameter	Citation not used to support parameter	Citation not used to support parameter	Citation not used to support parameter	

Dose parameter<br>Underpinning evidence	Type of strength exercise	Sets	Repetitions	Load	Intensity	Recovery	Method of progression	Frequency	Programme duration	Consistency rating
**Lemmey et al (2009)**	1. Chest press<br>2. Seated leg extension<br>3. Rowing<br>4. Bicep curl<br>5. Triceps extension<br>6. Leg press<br>7. Leg curl<br>8. Standing calf raises	2-3 sets	Weeks 1-4:<br>15 repetitions<br><br>Weeks 5-6:<br>12 repetitions<br><br>Weeks 7-24:<br>8 repetitions	Insufficiently described	Weeks 1-4:<br>60%1RM<br><br>Weeks 5-6:<br>70%1RM<br><br>Weeks 7-24:<br>80%1RM	1-2 minutes between sets	1RM reassessed every 4 weeks	2 x week	24 weeks	Exercise type:<br>Consistent<br><br>Sets:<br>Inconsistent<br><br>Repetitions:<br>Inconsistent<br><br>Load:<br>Unclear<br><br>Intensity:<br>Inconsistent<br><br>Recovery:<br>Consistent<br><br>Progression:<br>Inconsistent<br><br>Frequency:<br>Inconsistent<br><br>Duration:<br>Inconsistent
**Marcora et al (2005)**	1. Chest press<br>2. Seated leg extension<br>3. Rowing<br>4. Bicep curl<br>5. Triceps extension<br>6. Leg press<br>7. Leg curl<br>8. Standing calf raises	4 sets	Set 1:<br>15 repetitions<br><br>Sets 2-4:<br>8 repetitions<br><br>(Repetition velocity:<br>1-2 seconds concentric/ eccentric)	Insufficiently described	Set 1:<br>40%1RM<br><br>Sets 2-4:<br>80%1RM	1-2 minutes between sets and exercises	1RM reassessed at end of week 0, then every 2 weeks	3 x week	12 weeks	

Strasser et al (2007)

Slides 16-19

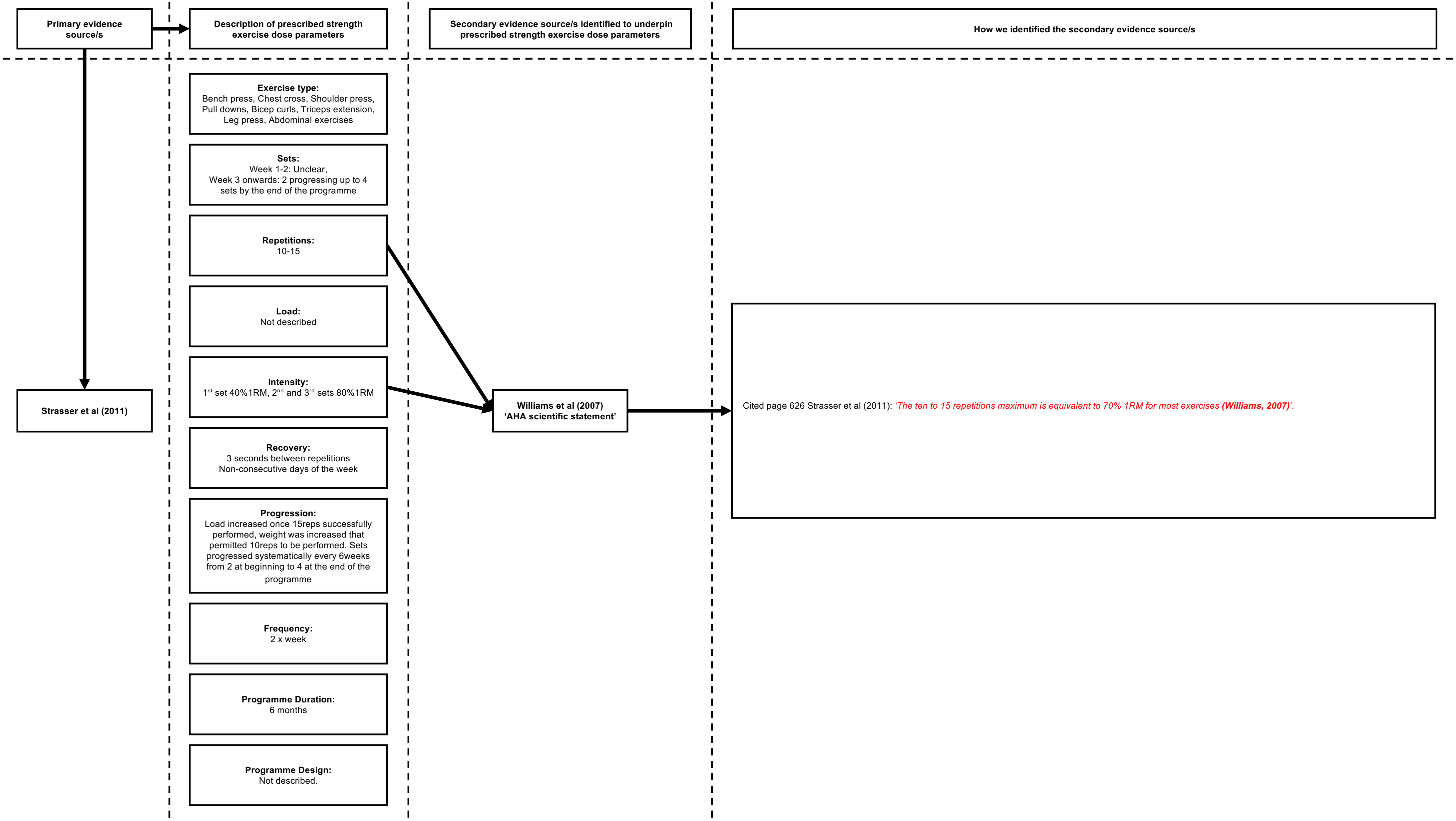
Primary evidence source/s
Description of prescribed strength exercise dose parameters
Secondary evidence source/s identified to underpin prescribed strength exercise dose parameters
How we identified the secondary evidence source/s
Strasser et al (2011)
Exercise type: Bench press, Chest cross, Shoulder press, Pull downs, Bicep curls, Triceps extension, Leg press, Abdominal exercises
Sets: Week 1-2: Unclear, Week 3 onwards: 2 progressing up to 4 sets by the end of the programme
Repetitions: 10-15
Load: Not described
Intensity: 1st set 40%1RM, 2nd and 3rd sets 80%1RM
Recovery: 3 seconds between repetitions Non-consecutive days of the week
Progression: Load increased once 15reps successfully performed, weight was increased that permitted 10reps to be performed. Sets progressed systematically every 6weeks from 2 at beginning to 4 at the end of the programme
Frequency: 2 x week
Programme Duration: 6 months
Programme Design: Not described.
Williams et al (2007) 'AHA scientific statement'
Cited page 626 Strasser et al (2011): 'The ten to 15 repetitions maximum is equivalent to 70% 1RM for most exercises (Williams, 2007)'.

Secondary evidence source/s identified to underpin prescribed strength exercise dose parameters

Oxford centre for evidence based medicine 2011 levels of evidence (OCEBM)

How we rated OCEBM level of evidence for the secondary evidence source/s

Williams et al (2007)
'AHA scientific statement'

→

OCEBM level
'Unclear'

→

Key information:

- Owing to Williams et al (2007) being identified as guideline, we we attempted to identify tertiary evidence source/s most appropriate to underpinning the identified prescription parameter/s it was used to support. In view that Strasser et al (2011) used Williams et al (2007) to underpin both repetitions and intensity, we searched Williams et al (2007) for tertiary evidence supporting these parameters.

Parameter: Repetitions and Intensity

- Page 579 from Williams et al (2007) describes Table 5 'Load-repetition relationship for resistance training'. We identified 1 tertiary evidence source (Dingwell et al, 2006) underpinning this table. Dingwell et al (2006) is a book chapter from Throw (2006) and describes exercise prescription in cardiac rehabilitation. Pages 111-113 describe exercise prescription for resistance training and table 4.1 located on page 113 describes 'Load-repetition relationship for resistance training'. It is unclear what evidence was used to support this table.

We rated OCEBM level of evidence for this source as 'unclear' because:

- It is unclear what evidence the table reported in the tertiary evidence source (Dingwell et al, 2006) is underpinned by.

Dose parameter / Underpinning evidence	Type of strength exercise	Sets	Repetitions	Load	Intensity	Recovery	Method of progression	Frequency	Programme duration	Consistency rating
**Strasser et al (2011)**	1. Bench press<br>2. Chest cross<br>3. Shoulder press<br>4. Pull downs<br>5. Bicep curls<br>6. Triceps extension<br>7. Leg press<br>8. Abdominal exercises	Week 1-2: Unclear<br><br>Week 3 onwards: 2 sets progressing up to 4 sets by the end of the programme	10-15 repetitions	Insufficiently described	1st set 40%1RM<br><br>2nd and 3rd sets 80%1RM	3 seconds between repetitions.<br><br>Non-consecutive days of the week	Load increased once 15reps successfully performed, weight was increased that permitted 10reps to be performed. Sets progressed systematically every 6weeks from 2 at beginning to 4 at the end of the programme.	2 x week	6 months	Exercise type: n/a<br><br>Sets: n/a<br><br>Repetitions: Consistent<br><br>Load: n/a<br><br>Intensity: Consistent<br><br>Recovery: n/a<br><br>Progression: n/a<br><br>Frequency: n/a<br><br>Duration: n/a
**Williams et al (2007)**	Citation not used to support parameter	Citation not used to support parameter	8-12 repetitions per set for healthy sedentary adults<br><br>or<br><br>10-15 repetitions at a low level of resistance, for example, <40% of 1RM, for older (>50-60 years of age), more frail persons, or cardiac patients	Citation not used to support parameter	40%1RM unclear for repetitions<br><br>80%1RM 8 repetitions	Citation not used to support parameter	Citation not used to support parameter	Citation not used to support parameter	Citation not used to support parameter	

van Rensburg et al (2012)

Slides 20-24

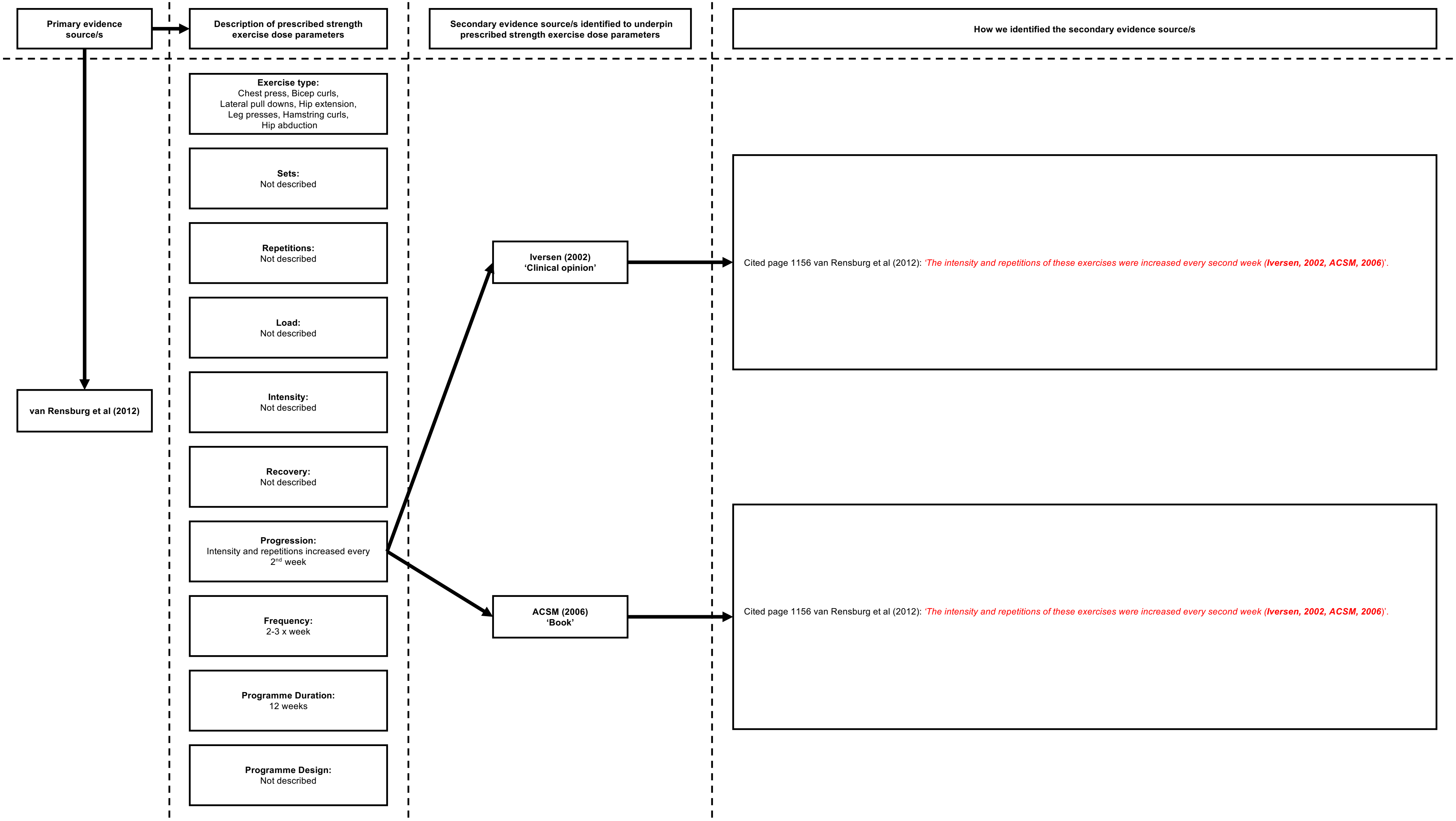
Primary evidence source/s
Description of prescribed strength exercise dose parameters
Secondary evidence source/s identified to underpin prescribed strength exercise dose parameters
How we identified the secondary evidence source/s
van Rensburg et al (2012)
Exercise type: Chest press, Bicep curls, Lateral pull downs, Hip extension, Leg presses, Hamstring curls, Hip abduction
Sets: Not described
Repetitions: Not described
Load: Not described
Intensity: Not described
Recovery: Not described
Progression: Intensity and repetitions increased every 2nd week
Frequency: 2-3 x week
Programme Duration: 12 weeks
Programme Design: Not described
Iversen (2002) 'Clinical opinion'
ACSM (2006) 'Book'
Cited page 1156 van Rensburg et al (2012): 'The intensity and repetitions of these exercises were increased every second week (Iversen, 2002, ACSM, 2006)'.
Cited page 1156 van Rensburg et al (2012): 'The intensity and repetitions of these exercises were increased every second week (Iversen, 2002, ACSM, 2006)'.

Secondary evidence source/s identified to underpin prescribed strength exercise dose parameters

Oxford centre for evidence based medicine 2011 levels of evidence (OCEBM)

How we rated OCEBM level of evidence for the secondary evidence source/s

---

Iversen (2002)
'Clinical opinion'

→ **OCEBM level**
'Unclear'

→

Key information:

- Iversen (2002) was not identified as pilot study for the primary evidence source, literature review or guideline. However, owing to Iversen (2002) being a clinical opinion, we attempted to identify tertiary evidence source/s most appropriate to underpinning the identified prescription parameter/s it was used to support. In view that van Rensburg et al (2012) used Iversen (2002) to underpin progression, we searched Iversen (2002) for tertiary evidence supporting this parameter.

Parameter: Progression

- Page 356-361 (Iversen, 2002) describe strengthening exercises being used during different stages of RA disease (active disease, subacute disease and inactive disease). We cautiously identified 5 tertiary evidence sources underpinning repetitions and intensity made up of 3 randomised controlled trials, 1 non-randomised controlled trial and 1 citation we were unable to retrieve.

We rated OCEBM level of evidence for this source as 'unclear' because:

- We rated the secondary evidence source as 'unclear' owing to the inadequate description of the parameters provided by the primary evidence source.

ACSM (2006)
'Book'

→ **OCEBM level**
'Incorrect citation'

→

Key information:

- ACSM (2006) was identified as a guideline, therefore we attempted to identify tertiary evidence source/s most appropriate to underpinning the identified prescription parameter/s it was used to support. In view that van Rensburg et al (2012) used ACSM (2006) to underpin repetitions, intensity and method of progression, we searched ACSM (2006) for tertiary evidence supporting these parameters. We were unable to cross-reference this with the primary evidence source because the secondary evidence source appeared incorrect.

We rated OCEBM level of evidence for this source as 'incorrect citation' because:

- The ACSM guidelines for exercise testing and prescription were published as a 7th edition in 2005 or an 8th edition in 2010. We were unable to locate the 2006 publication.

Dose parameter<br>Underpinning evidence	Type of strength exercise	Sets	Repetitions	Load	Intensity	Recovery	Method of progression	Frequency	Programme duration	Consistency rating
**van Rensburg et al (2012)**	1. Chest press<br>2. Bicep curls<br>3. Lateral pull downs<br>4. Hip extension<br>5. Leg presses<br>6. Hamstring curls<br>7. Hip abduction	Insufficiently described	Insufficiently described	Insufficiently described	Insufficiently described	Insufficiently described	Intensity and repetitions increased every 2nd week	2 x week	12 weeks	Exercise type: n/a<br>Sets: n/a<br>Repetitions: n/a<br>Load: n/a<br>Intensity: n/a<br>Recovery: n/a<br>Progression: Inconsistent<br>Frequency: n/a<br>Duration: n/a
**Iversen (2002)**	Citation not used to support parameter	Citation not used to support parameter	Citation not used to support parameter	Citation not used to support parameter	Citation not used to support parameter	Citation not used to support parameter	Isometric exercises are continued and the number of repetitions and sets can be increased as tolerated. Dynamic exercises with light resistance are now incorporated into the exercise regimen	Citation not used to support parameter	Citation not used to support parameter	

Dose parameter<br>Underpinning evidence	Type of strength exercise	Sets	Repetitions	Load	Intensity	Recovery	Method of progression	Frequency	Programme duration	Consistency rating
**van Rensburg et al (2012)**	1. Chest press<br>2. Bicep curls<br>3. Lateral pull downs<br>4. Hip extension<br>5. Leg presses<br>6. Hamstring curls<br>7. Hip abduction	Insufficiently described	Insufficiently described	Insufficiently described	Insufficiently described	Insufficiently described	Intensity and repetitions increased every 2nd week	2 x week	12 weeks	Exercise type:<br>n/a<br><br>Sets:<br>n/a<br><br>Repetitions:<br>n/a<br><br>Load:<br>n/a<br><br>Intensity:<br>n/a<br><br>Recovery:<br>n/a<br><br>Progression:<br>Unclear<br><br>Frequency:<br>n/a<br><br>Duration:<br>n/a
**ACSM (2006)**	Citation not used to support parameter	Citation not used to support parameter	Citation not used to support parameter	Citation not used to support parameter	Citation not used to support parameter	Citation not used to support parameter	Unclear	Citation not used to support parameter	Citation not used to support parameter	

Durcan et al (2014)

Slides 25-29

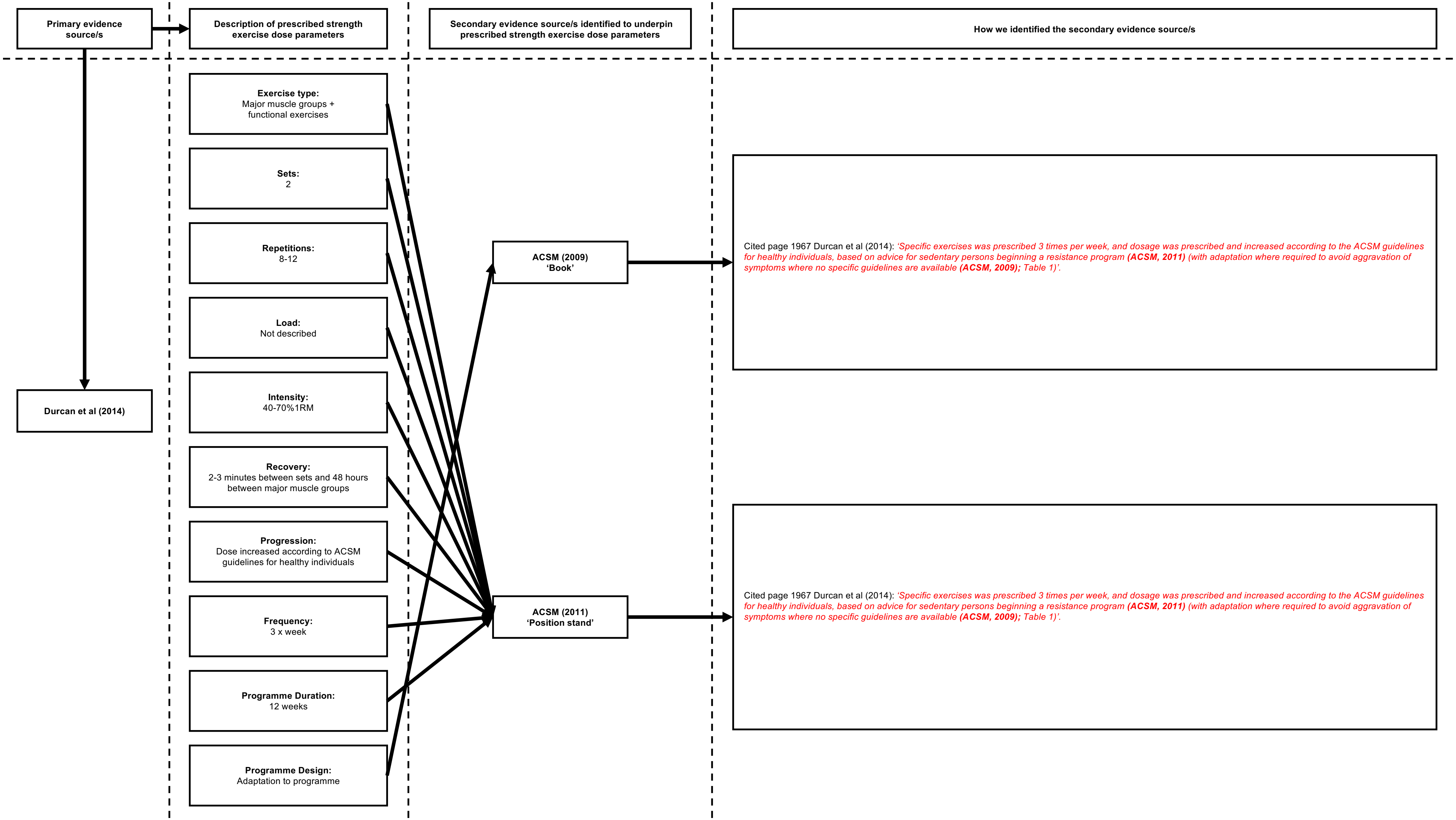

Primary evidence source/s
Description of prescribed strength exercise dose parameters
Secondary evidence source/s identified to underpin prescribed strength exercise dose parameters
How we identified the secondary evidence source/s
Durcan et al (2014)
Exercise type: Major muscle groups + functional exercises
Sets: 2
Repetitions: 8-12
Load: Not described
Intensity: 40-70%1RM
Recovery: 2-3 minutes between sets and 48 hours between major muscle groups
Progression: Dose increased according to ACSM guidelines for healthy individuals
Frequency: 3 x week
Programme Duration: 12 weeks
Programme Design: Adaptation to programme
ACSM (2009) 'Book'
ACSM (2011) 'Position stand'
Cited page 1967 Durcan et al (2014): 'Specific exercises was prescribed 3 times per week, and dosage was prescribed and increased according to the ACSM guidelines for healthy individuals, based on advice for sedentary persons beginning a resistance program (ACSM, 2011) (with adaptation where required to avoid aggravation of symptoms where no specific guidelines are available (ACSM, 2009); Table 1)'.
Cited page 1967 Durcan et al (2014): 'Specific exercises was prescribed 3 times per week, and dosage was prescribed and increased according to the ACSM guidelines for healthy individuals, based on advice for sedentary persons beginning a resistance program (ACSM, 2011) (with adaptation where required to avoid aggravation of symptoms where no specific guidelines are available (ACSM, 2009); Table 1)'.

Secondary evidence source/s identified to underpin prescribed strength exercise dose parameters	Oxford centre for evidence based medicine 2011 levels of evidence (OCEBM)	How we rated OCEBM level of evidence for the secondary evidence source/s
**ACSM (2009) 'Book'**	**OCEBM level 'Unclear'**	**Key information:**<br>▪ ACSM (2009) was not identified as pilot study for the primary evidence source, literature review or guideline. However, owing to ASCM (2009) being a book, we attempted to identify tertiary evidence source/s most appropriate to underpinning the identified prescription parameter/s it was used to support. In view that Durcan et al (2014) used ACSM (2009) to underpin adapting the dosage to avoid aggravation of symptoms, we searched ACSM (2009) for tertiary evidence supporting this parameter. We unable to identify tertiary evidence source/s underpinning this parameter.<br><br>**We rated OCEBM level of evidence for this source as 'unclear' because:**<br>▪ We rated the secondary evidence source as 'unclear' as we were unable to identify how the secondary evidence source underpinned adapting the dosage to avoid aggravation of symptoms.
**ACSM (2011) 'Position stand'**	**OCEBM level '2-5'**	**Background:**<br>▪ ACSM (2011) was identified as a guideline, we attempted to identify tertiary evidence source/s most appropriate to underpinning the identified prescription parameter/s it was used to support. In view that Durcan et al (2014) used ACSM (2011) to underpin exercise type, sets, repetitions, load, intensity, recovery, progression and frequency, we searched ACSM (2011) for tertiary evidence supporting these parameters.<br><br>Parameters: Exercise type, sets, repetitions, load, intensity, recovery, progression and frequency<br>▪ We refined our search in view that Durcan et al (2014) prescribed the dose according to the advice for sedentary persons beginning a resistance programme. it is likely the primary evidence source used the evidence-based recommendations table located on page 1336. This table does not directly link to the citations contained in the references section. However tertiary evidence may cautiously be identified under the headings 'What types of exercises improve muscular fitness?' and 'Are there differences in resistance training recommendations according to individual characteristics?' because 'novice exercisers and those who are older, very deconditioned, or frail' are described. We identified 9 citations that be be relevant to underpinning prescription of strength exercise in those beginning a programme. These included included 3 RCTs, 2 ACSM position stands, 2 cohort studies, 1 non-randomised controlled trial and 1 systematic review.<br><br>**We rated OCEBM level of evidence for this source as '2-5' (Randomised trial or observational study with dramatic effect to mechanism-based reasoning) because:**<br>▪ The tertiary evidence sources identified as most likely underpinning the parameter ranged between these two levels of evidence.

Dose parameter / Underpinning evidence	Type of strength exercise	Sets	Repetitions	Load	Intensity	Recovery	Method of progression	Frequency	Programme duration	Consistency rating
**Durcan et al (2014)**	Major muscle groups + functional exercises	2 sets	8-12 repetitions	Insufficiently described	40-70%1RM	2-3 minutes between sets and 48 hours between major muscle groups	Dose increased according to ACSM guidelines for healthy individuals	3 x week	12 weeks	Exercise type: Unclear<br>Sets: Unclear<br>Repetitions: Consistent<br>Load: Unclear<br>Intensity: Unclear<br>Recovery: Unclear<br>Progression: Unclear<br>Frequency: Unclear<br>Duration: Unclear
**ACSM (2009)**	Unclear	Unclear	Unclear	Unclear	Unclear	Unclear	Unclear	Unclear	Unclear	

Dose parameter / Underpinning evidence	Type of strength exercise	Sets	Repetitions	Load	Intensity	Recovery	Method of progression	Frequency	Programme duration	Consistency rating
**Durcan et al (2014)**	Major muscle groups + functional exercises	2 sets	8-12 repetitions	Insufficiently described	40-70%1RM	2-3 minutes between sets and 48 hours between major muscle groups	Dose increased according to ACSM guidelines for healthy individuals	3 x week	12 weeks	Exercise type: Consistent<br>Sets: Consistent<br>Repetitions: Consistent<br>Load: Unclear<br>Intensity: Consistent<br>Recovery: Consistent<br>Progression: Consistent<br>Frequency: Consistent<br>Duration: Unclear
**ACSM (2011)**	Resistance exercise involving each major muscle group are recommended. A variety of exercise equipment and/or body weight can be used to perform these exercises.	1-4 sets	8-20 repetitions	Unclear	40-≥80%1RM	Rest intervals of 2-3minutes between each set of repetitions are effective. A rest of ≥48 hours between sessions for any single muscle group is recommended	A gradual progression of greater resistance and/or more repetitions per set and/or increasing frequency is recommended.	Each major muscle group should be trained on 2-3 x week.	No specific duration of training has been identified for effectiveness.	

Manning et al (2014)

Slides 30-33

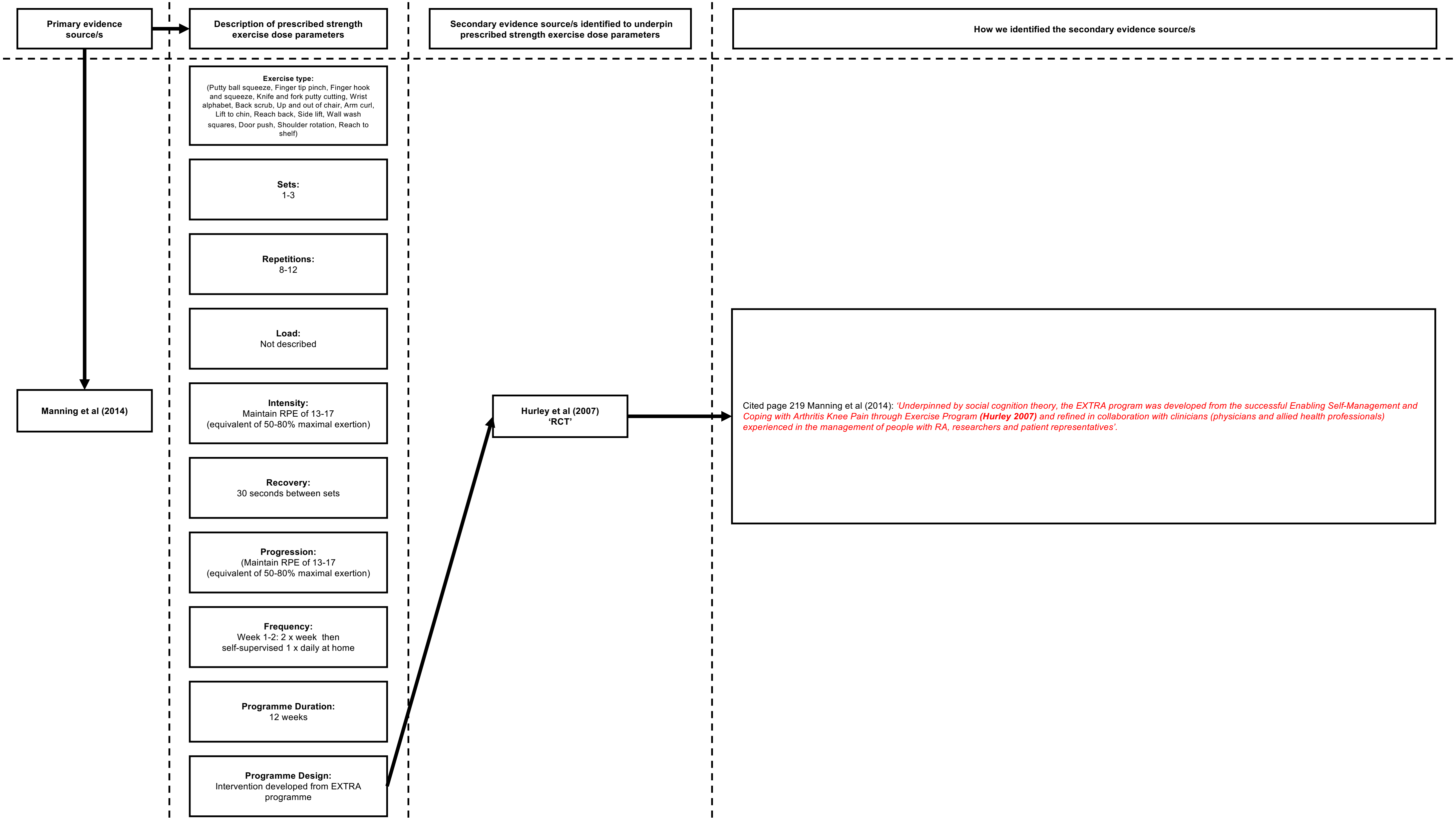
Primary evidence source/s
Description of prescribed strength exercise dose parameters
Secondary evidence source/s identified to underpin prescribed strength exercise dose parameters
How we identified the secondary evidence source/s
Manning et al (2014)
Exercise type:
(Putty ball squeeze, Finger tip pinch, Finger hook and squeeze, Knife and fork putty cutting, Wrist alphabet, Back scrub, Up and out of chair, Arm curl, Lift to chin, Reach back, Side lift, Wall wash squares, Door push, Shoulder rotation, Reach to shelf)
Sets:
1-3
Repetitions:
8-12
Load:
Not described
Intensity:
Maintain RPE of 13-17
(equivalent of 50-80% maximal exertion)
Recovery:
30 seconds between sets
Progression:
(Maintain RPE of 13-17
(equivalent of 50-80% maximal exertion)
Frequency:
Week 1-2: 2 x week then
self-supervised 1 x daily at home
Programme Duration:
12 weeks
Programme Design:
Intervention developed from EXTRA programme
Hurley et al (2007)
'RCT'
Cited page 219 Manning et al (2014): *'Underpinned by social cognition theory, the EXTRA program was developed from the successful Enabling Self-Management and Coping with Arthritis Knee Pain through Exercise Program* ***(Hurley 2007)*** *and refined in collaboration with clinicians (physicians and allied health professionals) experienced in the management of people with RA, researchers and patient representatives'.*

Secondary evidence source/s identified to underpin prescribed strength exercise dose parameters	Oxford centre for evidence based medicine 2011 levels of evidence (OCEBM)	How we rated OCEBM level of evidence for the secondary evidence source/s
**Hurley et al (2007)**<br>**'RCT'** →	**OCEBM level**<br>**'2'** →	**Key information:**<br>▪ Hurley et al (2007) was not identified as a pilot study for the primary evidence source, literature review or guideline. Therefore, as stipulated in the study protocol we did not identify tertiary evidence source/s.<br><br>**We rated OCEBM level of evidence for this source as '2' (Randomised trial or observational study with dramatic effect) because:**<br>▪ Hurley et al (2007) is a randomised controlled trial.

Dose parameter<br>Underpinning evidence	Type of strength exercise	Sets	Repetitions	Load	Intensity	Recovery	Method of progression	Frequency	Programme duration	Consistency rating
**Manning et al (2014)**	1. (Putty ball squeeze<br>2. Finger tip pinch<br>3. Finger hook and squeeze<br>4. Knife and fork putty cutting<br>5. Wrist alphabet<br>6. Back scrub<br>7. Up and out of chair<br>8. Arm curl<br>9. Lift to chin<br>10. Reach back<br>11. Side lift<br>12. Wall wash squares<br>13. Door push<br>14. Shoulder rotation<br>15. Reach to shelf	1-3 sets	8-12 repetitions	Insufficiently described	Maintain RPE of 13-17 (equivalent of 50-80% maximal exertion)	30 seconds between sets	(Maintain RPE of 13-17 (equivalent of 50-80% maximal exertion	Week 1-2: 2 x week then self-supervised 1 x daily at home	12 weeks	Exercise type: Inconsistent<br>Sets: Unclear<br>Repetitions: Inconsistent<br>Load: Unclear<br>Intensity: Unclear<br>Recovery: Unclear<br>Progression: Inconsistent<br>Frequency: Inconsistent<br>Duration: Inconsistent
**Hurley et al (2007)**	1. Exercise bike<br>2. Quadriceps bench<br>3. Theraband<br>4. Sit to stand<br>5. Step ups<br>6. Wall squats<br>7. Step downs<br>8. Knee wedge	1. Unclear<br>2. Unclear<br>3. Unclear<br>4. Unclear<br>5. Unclear<br>6. Unclear<br>7. Unclear<br>8. Unclear	1. 5 minutes<br>2. 24 reps<br>3. 2 minutes<br>4. 2 minutes<br>5. 1 minute<br>6. 1 minute<br>7. 1 minute<br>8. 1 minute	1. Unclear<br>2. Unclear<br>3. Light/moderate/heavy<br>4. Unclear<br>5. Unclear<br>6. Unclear<br>7. Unclear<br>8. 0-5kg	1. Unclear<br>2. Unclear<br>3. Unclear<br>4. Unclear<br>5. Unclear<br>6. Unclear<br>7. Unclear<br>8. Unclear	1. Unclear<br>2. 5 secs<br>3. Unclear<br>4. Unclear<br>5. Unclear<br>6. Unclear<br>7. Unclear<br>8. Unclear	1. Increase time or resistance<br>2. Increase repetitions<br>3. Increase time or resistance<br>4. Increase time or decrease seat height<br>5. Increase time or step height (low, medium, high)<br>6. Increase time<br>7. Progression of exercise 6, increase time<br>8. Increase load<br>9. Unclear	2 x week	6 weeks	

Lamb et al (2015)

Slides 34-42

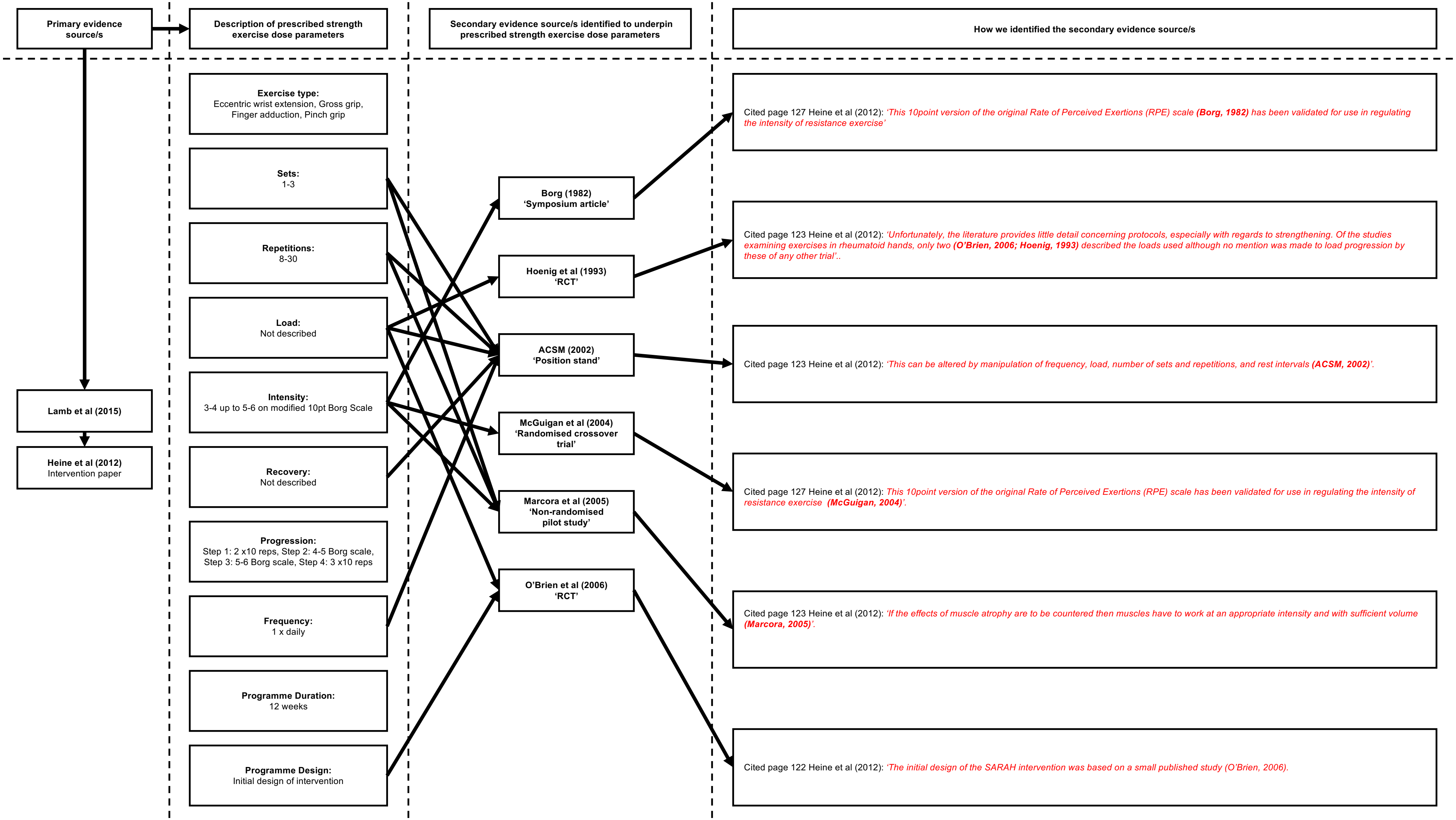

Primary evidence source/s
Description of prescribed strength exercise dose parameters
Secondary evidence source/s identified to underpin prescribed strength exercise dose parameters
How we identified the secondary evidence source/s
Lamb et al (2015)
Heine et al (2012) Intervention paper
Exercise type: Eccentric wrist extension, Gross grip, Finger adduction, Pinch grip
Sets: 1-3
Repetitions: 8-30
Load: Not described
Intensity: 3-4 up to 5-6 on modified 10pt Borg Scale
Recovery: Not described
Progression: Step 1: 2 x10 reps, Step 2: 4-5 Borg scale, Step 3: 5-6 Borg scale, Step 4: 3 x10 reps
Frequency: 1 x daily
Programme Duration: 12 weeks
Programme Design: Initial design of intervention
Borg (1982) 'Symposium article'
Hoenig et al (1993) 'RCT'
ACSM (2002) 'Position stand'
McGuigan et al (2004) 'Randomised crossover trial'
Marcora et al (2005) 'Non-randomised pilot study'
O'Brien et al (2006) 'RCT'
Cited page 127 Heine et al (2012): 'This 10point version of the original Rate of Perceived Exertions (RPE) scale (Borg, 1982) has been validated for use in regulating the intensity of resistance exercise'
Cited page 123 Heine et al (2012): 'Unfortunately, the literature provides little detail concerning protocols, especially with regards to strengthening. Of the studies examining exercises in rheumatoid hands, only two (O'Brien, 2006; Hoenig, 1993) described the loads used although no mention was made to load progression by these of any other trial'..
Cited page 123 Heine et al (2012): 'This can be altered by manipulation of frequency, load, number of sets and repetitions, and rest intervals (ACSM, 2002)'.
Cited page 127 Heine et al (2012): This 10point version of the original Rate of Perceived Exertions (RPE) scale has been validated for use in regulating the intensity of resistance exercise (McGuigan, 2004)'.
Cited page 123 Heine et al (2012): 'If the effects of muscle atrophy are to be countered then muscles have to work at an appropriate intensity and with sufficient volume (Marcora, 2005)'.
Cited page 122 Heine et al (2012): 'The initial design of the SARAH intervention was based on a small published study (O'Brien, 2006).

Secondary evidence source/s identified to underpin prescribed strength exercise dose parameters

Oxford centre for evidence based medicine 2011 levels of evidence (OCEBM)

How we rated OCEBM level of evidence for the secondary evidence source/s

---

Borg (1982)
'Symposium article'

OCEBM level
'5'

Key information:
- Borg (1982) was not identified as pilot study for the primary evidence source, literature review or guideline. Therefore, we did not identify tertiary evidence source/s.

We rated OCEBM level of evidence for this source as '5' because:
- Borg (1982) is a symposium article.

Hoenig et al (1993)
'RCT'

OCEBM level
'2'

Key information:
- Hoenig et al (1993) was not identified as pilot study for the primary evidence source, literature review or guideline. Therefore, we did not identify tertiary evidence source/s.

We rated OCEBM level of evidence for this source as '2' (Randomised trial or observational study with dramatic effect) because:
- Hoenig et al (1993) is a randomised controlled trial.

ACSM (2002)
'Position stand'

OCEBM level
'2-5'

Key information:
- ACSM (2002) was identified as a guideline. Therefore, we attempted to identify tertiary evidence source/s most appropriate to underpinning the identified prescription parameter/s it was used to support. In view that Lamb et al (2015) via Heine et al (2012) used ACSM (2002) to underpin sets, repetitions, load and recovery and frequency, we searched ACSM (2002) for tertiary evidence supporting these parameters.

Parameter: Sets and Repetitions (Volume) and Loading
- We groups these parameters together as the secondary evidence occasionally grouped these together (e.g. page 370 and 372), making differentiation difficult. Pages 367, 370 and 372 contained citations. We cautiously identified 13 cohort studies, 8 non-randomised controlled trials, 7 unknown citations, 6 randomised controlled trials, 2 non-systematic reviews, 2 books and 1 book chapter.

Parameter: Recovery
- Pages 368, 370 and 372 contained citations. We cautiously identified 5 cohort studies, 2 citations, 1 randomised controlled trial and 1 non-randomised controlled trial.

Parameter: Frequency
- Pages 369 and 371 contained citations. We cautiously identified 7 cohort studies, 5 randomised controlled trials, 5 non-randomised controlled trials, 2 books and 1 unknown citation.

We rated OCEBM level of evidence for this source '2-5' (Randomised trial or observational study with dramatic effect to mechanism-based reasoning) because:
- The tertiary evidence underpinning training volumes and intensities considered optimal for inducing muscle hypertrophy ranged between these two levels of evidence.

McGuigan et al (2004)
'Randomised crossover trial'

OCEBM level
'2'

Key information:
McGuigan et al (2004) was not identified as pilot study for the primary evidence source, literature review or guideline. Therefore, we did not identify tertiary evidence source/s

We rated this secondary evidence source level 2 (Randomised trial or observational study with dramatic effect) because:
- McGuigan et al (2004) is randomised cross-over trial.

Marcora et al (2005)
'Non-randomised pilot study'

OCEBM level
'3'

Key information:
- Marcora et al (2005) was not identified as pilot study for the primary evidence source, literature review or guideline. Therefore, we did not identify tertiary evidence source/s.

We rated OCEBM level of evidence for this source as '3' (Non-Randomised controlled cohort/follow-up study) because:
- Marcora et al (2005) is a non-randomised 2-group, matched, parallel, controlled, pretest-posttest study

---

O'Brien et al (2006)
'RCT'

OCEBM level
'2'

Key information:
- O'Brien et al (2006) was not identified as pilot study for the primary evidence source, literature review or guideline. Therefore, we did not identify tertiary evidence source/s.

We rated this secondary evidence source level 2 (Randomised trial or observational study with dramatic effect) because:
- O'Brien et al (2006) is a randomised controlled trial.

Dose parameter / Underpinning evidence	Type of strength exercise	Sets	Repetitions	Load	Intensity	Recovery	Method of progression	Frequency	Programme duration	Consistency rating
**Lamb et al (2015)**	1. Eccentric wrist extension<br>2. Gross grip<br>3. Finger adduction<br>4. Pinch grip	1-3 sets	8-30 repetitions	Insufficiently described	3-4 to 5-6 on modified Borg scale	Insufficiently described	Step 1: 2 x10 reps<br>Step 2: 4-5 Borg scale<br>Step 3: 5-6 Borg scale<br>Step 4: 3 x10 reps	1 x daily	12 weeks	Exercise type: n/a<br>Sets: n/a<br>Repetitions: n/a<br>Load: Unclear<br>Intensity: Unclear<br>Recovery: n/a<br>Progression: n/a<br>Frequency: n/a<br>Duration: n/a
**Borg (1982)**	Citation not used to support parameter	Citation not used to support parameter	Citation not used to support parameter	Citation not used to support parameter	Unclear	Citation not used to support parameter	Citation not used to support parameter	Citation not used to support parameter	Citation not used to support parameter	

Dose parameter<br>Underpinning evidence	Type of strength exercise	Sets	Repetitions	Load	Intensity	Recovery	Method of progression	Frequency	Programme duration	Consistency rating
**Lamb et al (2015)**	1. Eccentric wrist extension<br>2. Gross grip<br>3. Finger adduction<br>4. Pinch grip	1-3 sets	8-30 repetitions	Insufficiently described	3-4 to 5-6 on modified Borg scale	Insufficiently described	Step 1:<br>2 x10 reps<br><br>Step 2:<br>4-5 Borg scale<br><br>Step 3:<br>5-6 Borg scale<br><br>Step 4:<br>3 x10 reps	1 x daily	12 weeks	Exercise type:<br>n/a<br><br>Sets:<br>n/a<br><br>Repetitions:<br>n/a<br><br>Load:<br>Unclear<br><br>Intensity:<br>n/a<br><br>Recovery:<br>n/a<br><br>Progression:<br>n/a<br><br>Frequency:<br>n/a<br><br>Duration:<br>n/a
**Hoenig et al (1993)**	Citation not used to support parameter	Citation not used to support parameter	Citation not used to support parameter	Theraputty with a plasticity rating of 85 (medium soft grade/BeOK red)	Citation not used to support parameter	Citation not used to support parameter	Citation not used to support parameter	Citation not used to support parameter	Citation not used to support parameter	

Dose parameter<br>Underpinning evidence	Type of strength exercise	Sets	Repetitions	Load	Intensity	Recovery	Method of progression	Frequency	Programme duration	Consistency rating
**Lamb et al (2015)**	1. Eccentric wrist extension<br>2. Gross grip<br>3. Finger adduction<br>4. Pinch grip	1-3 sets	8-30 repetitions	Insufficiently described	3-4 to 5-6 on modified Borg scale	Insufficiently described	Step 1:<br>2 x10 reps<br><br>Step 2:<br>4-5 Borg scale<br><br>Step 3:<br>5-6 Borg scale<br><br>Step 4:<br>3 x10 reps	1 x daily	12 weeks	Exercise type:<br>n/a<br><br>Sets:<br>Consistent<br><br>Repetitions:<br>Consistent<br><br>Load:<br>Unclear<br><br>Intensity:<br>n/a<br><br>Recovery:<br>Unclear<br><br>Progression:<br>n/a<br><br>Frequency:<br>Inconsistent<br><br>Duration:<br>n/a
**ACSM (2002)**	Citation not used to support parameter	1-3	8-12	Novice<br>60-70%1RM	Citation not used to support parameter	2-3 minutes for core and 1-2 minutes for others	Citation not used to support parameter	Novice<br>2-3 x week<br><br>Intermediate<br>2-4 x week<br><br>Advanced<br>4-6 x week	Citation not used to support parameter	

**Dose parameter**<br>**Underpinning evidence**	**Type of strength exercise**	**Sets**	**Repetitions**	**Load**	**Intensity**	**Recovery**	**Method of progression**	**Frequency**	**Programme duration**	**Consistency rating**
**Lamb et al (2015)**	1. Eccentric wrist extension<br>2. Gross grip<br>3. Finger adduction<br>4. Pinch grip	1-3 sets	8-30 repetitions	Insufficiently described	3-4 to 5-6 on modified Borg scale	Insufficiently described	Step 1:<br>2 x10 reps<br><br>Step 2:<br>4-5 Borg scale<br><br>Step 3:<br>5-6 Borg scale<br><br>Step 4:<br>3 x10 reps	1 x daily	12 weeks	Exercise type:<br>n/a<br><br>Sets:<br>n/a<br><br>Repetitions:<br>n/a<br><br>Load:<br>n/a<br><br>Intensity:<br>Unclear<br><br>Recovery:<br>n/a<br><br>Progression:<br>n/a<br><br>Frequency:<br>n/a<br><br>Duration:<br>n/a
**McGuigan et al (2002)**	Citation not used to support parameter	Citation not used to support parameter	Citation not used to support parameter	Citation not used to support parameter	Unclear	Citation not used to support parameter	Citation not used to support parameter	Citation not used to support parameter	Citation not used to support parameter	

Dose parameter / Underpinning evidence	Type of strength exercise	Sets	Repetitions	Load	Intensity	Recovery	Method of progression	Frequency	Programme duration	Consistency rating
**Lamb et al (2015)**	1. Eccentric wrist extension<br>2. Gross grip<br>3. Finger adduction<br>4. Pinch grip	1-3 sets	8-30 repetitions	Insufficiently described	3-4 to 5-6 on modified Borg scale	Insufficiently described	Step 1: 2 x10 reps<br>Step 2: 4-5 Borg scale<br>Step 3: 5-6 Borg scale<br>Step 4: 3 x10 reps	1 x daily	12 weeks	Exercise type: Inconsistent<br>Sets: Inconsistent<br>Repetitions: Inconsistent<br>Load: n/a<br>Intensity: Inconsistent<br>Recovery: n/a<br>Progression: n/a<br>Frequency: n/a<br>Duration: n/a
**Marcora et al (2005)**	1. Chest press<br>2. Seated leg extension<br>3. Seated row<br>4. Bicep curl<br>5. Triceps extension<br>6. Leg press<br>7. Leg curl<br>8. Standing calf raises	4 sets	Set 1: 15 repetitions<br>Sets 2-4: 8 repetitions<br>(Repetition velocity: 1-2 seconds concentric/ Eccentric)	Citation not used to support parameter	Set 1: 40%1RM<br>Sets 2-4: 80%1RM	Citation not used to support parameter	Citation not used to support parameter	Citation not used to support parameter	Citation not used to support parameter	

Dose parameter / Underpinning evidence	Type of strength exercise	Sets	Repetitions	Load	Intensity	Recovery	Method of progression	Frequency	Programme duration	Consistency rating
**Lamb et al (2015)**	1. Eccentric wrist extension<br>2. Gross grip<br>3. Finger adduction<br>4. Pinch grip	1-3 sets	8-30 repetitions	Insufficiently described	3-4 to 5-6 on modified Borg scale	Insufficiently described	Step 1: 2 x10 reps<br>Step 2: 4-5 Borg scale<br>Step 3: 5-6 Borg scale<br>Step 4: 3 x10 reps	1 x daily	12 weeks	Exercise type: Consistent<br>Sets: Unclear<br>Repetitions: Inconsistent<br>Load: Unclear<br>Intensity: Unclear<br>Recovery: Unclear<br>Progression: Inconsistent<br>Frequency: Inconsistent<br>Duration: Inconsistent
**O' Brien et al (2006)**	1. Pinch grip using a towel<br>2. Wrist extension using theratube resistive band.	Unclear	Baseline: 5 repetitions<br>1 month: 10 repetitions<br>3 months: 20 repetitions	Unclear	Unclear	Unclear	Increase repetitions from 5 at baseline to 10 at 1 month and 20 repetitions of the exercises from 3 months onwards.	2 x daily	6 months	

Seneca et al (2015)

Slides 43-46

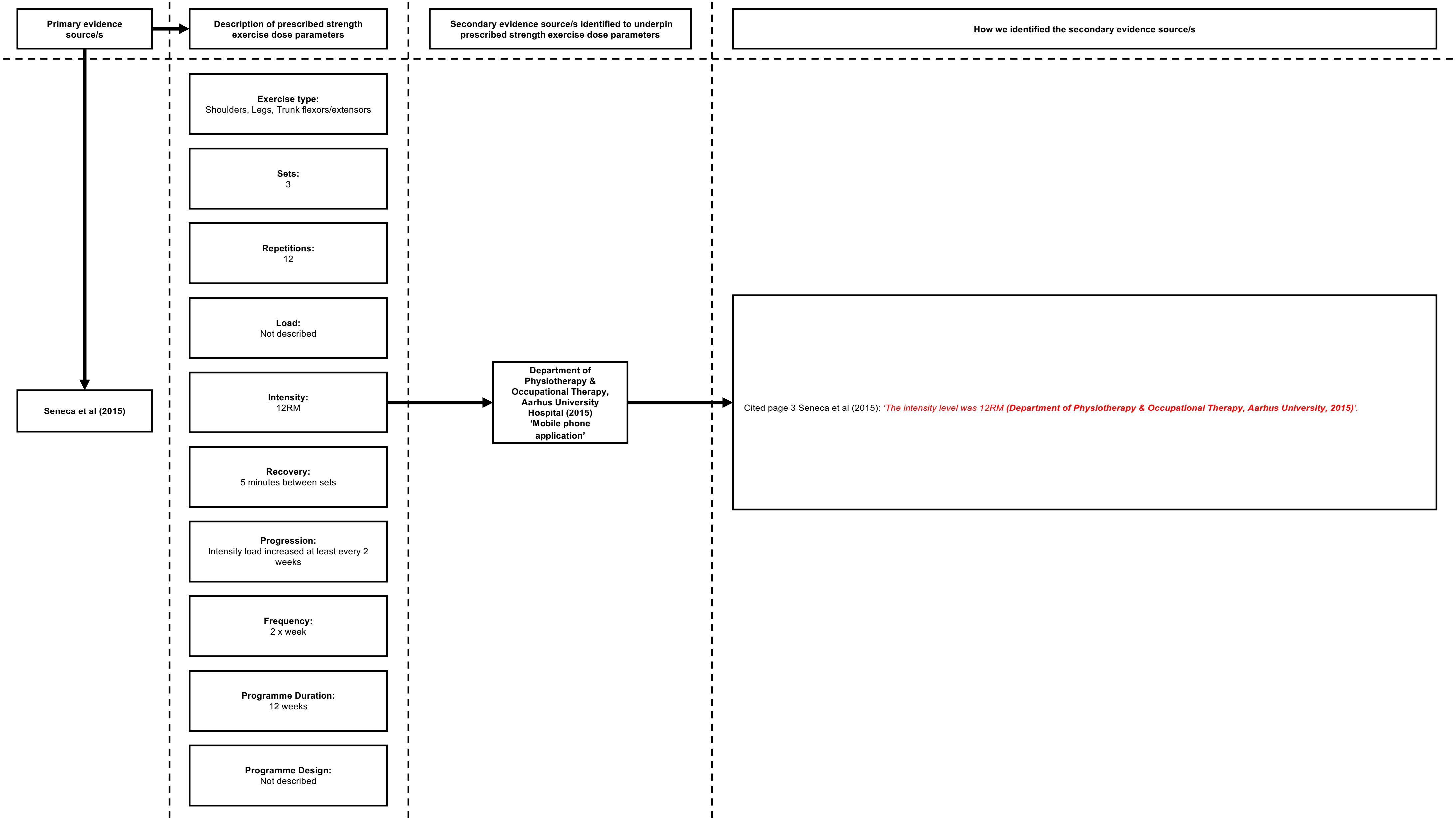
Primary evidence source/s
Description of prescribed strength exercise dose parameters
Secondary evidence source/s identified to underpin prescribed strength exercise dose parameters
How we identified the secondary evidence source/s
Exercise type: Shoulders, Legs, Trunk flexors/extensors
Sets: 3
Repetitions: 12
Load: Not described
Seneca et al (2015)
Intensity: 12RM
Department of Physiotherapy & Occupational Therapy, Aarhus University Hospital (2015) 'Mobile phone application'
Cited page 3 Seneca et al (2015): 'The intensity level was 12RM (Department of Physiotherapy & Occupational Therapy, Aarhus University, 2015)'.
Recovery: 5 minutes between sets
Progression: Intensity load increased at least every 2 weeks
Frequency: 2 x week
Programme Duration: 12 weeks
Programme Design: Not described

Secondary evidence source/s identified to underpin prescribed strength exercise dose parameters

Oxford centre for evidence based medicine 2011 levels of evidence (OCEBM)

How we rated OCEBM level of evidence for the secondary evidence source/s

Department of Physiotherapy & Occupational Therapy, Aarhus University Hospital (2015) 'Mobile phone application'

→

OCEBM level 'Unclear'

→

Key information

- Department of Physiotherapy & Occupational Therapy, Aarhus University Hospital (2015) was not identified as a pilot study for the primary evidence source, literature review or guideline. However, owing to Department of Physiotherapy & Occupational Therapy, Aarhus University Hospital (2015) being a mobile phone application, we attempted to identify tertiary evidence source/s most appropriate to underpinning the identified prescription parameter/s it was used to support. In view that Seneca et al (2015) used Department of Physiotherapy & Occupational Therapy, Aarhus University Hospital (2015) to underpin intensity, we searched Department of Physiotherapy & Occupational Therapy, Aarhus University Hospital (2015) for tertiary evidence supporting this parameter. We were unable to identify tertiary evidence source/s.

We rated OCEBM level of evidence for this source as 'unclear' because:

- The evidence source was a mobile phone application and the secondary evidence did not provide tertiary evidence sources.

Dose parameter / Underpinning evidence	Type of strength exercise	Sets	Repetitions	Load	Intensity	Recovery	Method of progression	Frequency	Programme duration	Consistency rating
**Seneca et al (2015)**	1. Shoulders<br>2. Legs<br>3. Trunk flexors/extensors	3 sets	12 repetitions	Insufficiently described	12RM (Repetition Maximum)	5 minutes between sets	Intensity load increased at least every 2 weeks	2 x week	12 weeks	Exercise type: n/a<br>Sets: n/a<br>Repetitions: Inconsistent<br>Load: n/a<br>Intensity: Consistent<br>Recovery: n/a<br>Progression: n/a<br>Frequency: n/a<br>Duration: n/a
**Department of Physiotherapy & Occupational Therapy, Aarhus University Hospital (2015)**	Citation not used to support parameter	Citation not used to support parameter	Citation not used to support parameter	Citation not used to support parameter	The right workout weight is the weight you can just lift 12 times.	Citation not used to support parameter	Citation not used to support parameter	Citation not used to support parameter	Citation not used to support parameter	

Dulgeroglu et al (2016)

Slides 48-51

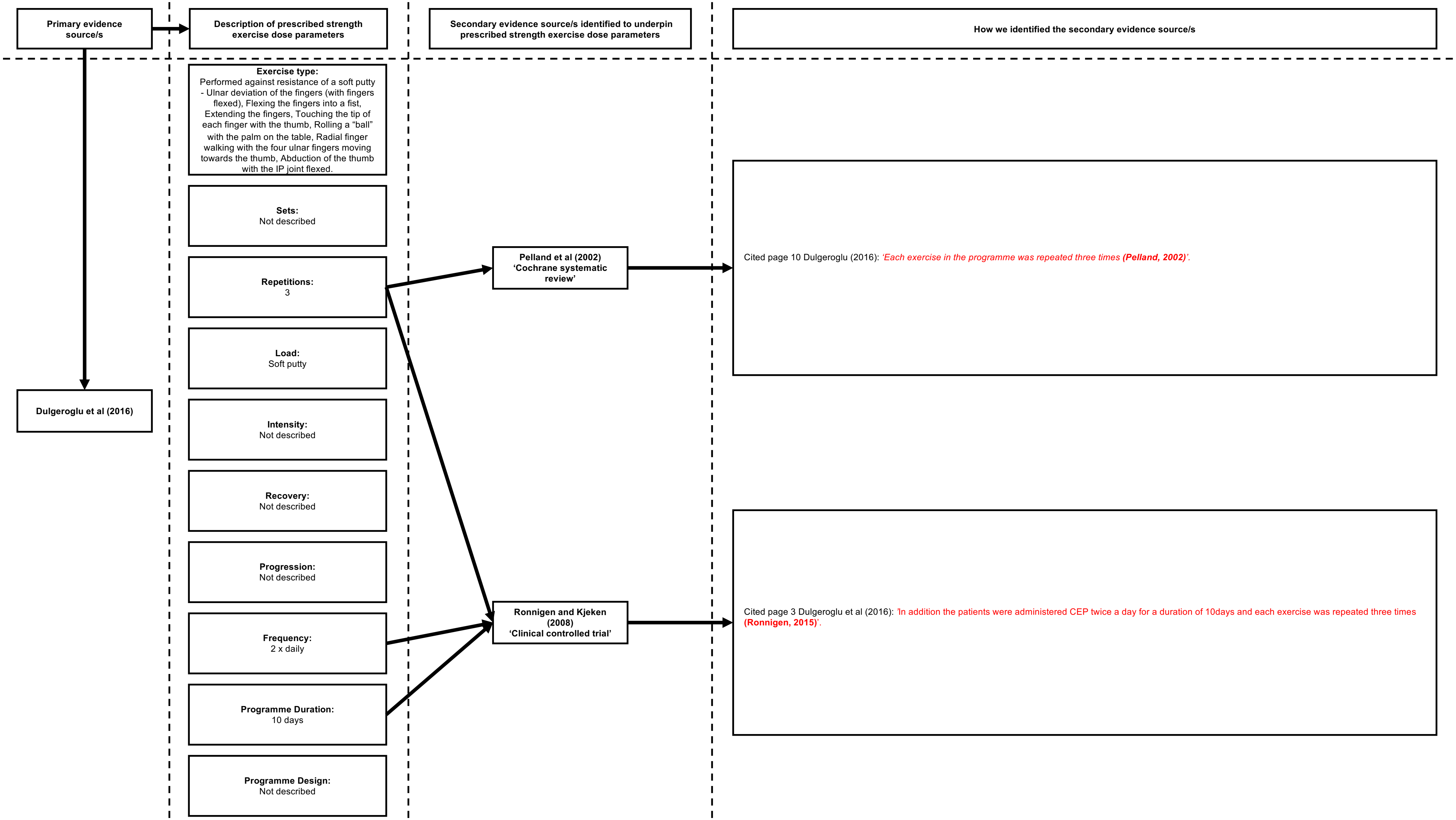
Primary evidence source/s
Description of prescribed strength exercise dose parameters
Secondary evidence source/s identified to underpin prescribed strength exercise dose parameters
How we identified the secondary evidence source/s
Exercise type: Performed against resistance of a soft putty - Ulnar deviation of the fingers (with fingers flexed), Flexing the fingers into a fist, Extending the fingers, Touching the tip of each finger with the thumb, Rolling a "ball" with the palm on the table, Radial finger walking with the four ulnar fingers moving towards the thumb, Abduction of the thumb with the IP joint flexed.
Sets: Not described
Repetitions: 3
Load: Soft putty
Intensity: Not described
Recovery: Not described
Progression: Not described
Frequency: 2 x daily
Programme Duration: 10 days
Programme Design: Not described
Dulgeroglu et al (2016)
Pelland et al (2002) 'Cochrane systematic review'
Ronnigen and Kjeken (2008) 'Clinical controlled trial'
Cited page 10 Dulgeroglu (2016): 'Each exercise in the programme was repeated three times (Pelland, 2002)'.
Cited page 3 Dulgeroglu et al (2016): 'In addition the patients were administered CEP twice a day for a duration of 10days and each exercise was repeated three times (Ronnigen, 2015)'.

Secondary evidence source/s identified to underpin prescribed strength exercise dose parameters	Oxford centre for evidence based medicine 2011 levels of evidence (OCEBM)	How we rated OCEBM level of evidence for the secondary evidence source/s
**Pelland et al (2002) 'Cochrane systematic review'** →	**OCEBM Level 'Incorrect citation'** →	**Key information:** ▪ Pelland et al (2002) was identified as a literature review, therefore we attempted to identify tertiary evidence source/s most appropriate to underpinning the identified prescription parameter/s it was used to support. In view that Dulgeroglu et al (2016) used Pelland et al (2002) to underpin repetitions, we searched Pelland (2002) for tertiary evidence supporting these parameters.<br>Parameters: Repetitions.<br>▪ We refined our search to identify the included studies in the review. We identified 1 tertiary evidence source (Oldham and Stanley, 1989) included (met inclusion criteria) in the systematic review. (Oldham and Stanley,1989) investigated two methods of electrical stimulation and involved 15 participants with unequal allocation between the intervention (n=12) and 1 control (n=3) groups.<br>**We rated OCEBM level of evidence for this source as 'incorrect citation' because:**<br>▪ Both secondary and tertiary evidence sources related to electrostimulation and not volitional strength exercise.<br>.
**Ronnigen and Kjeken (2008) 'Clinical controlled trial** →	**OCEBM level '3'** →	**Key information:**<br>▪ Ronnigen and Kjeken (2008) was not identified as a pilot study for the primary evidence source, literature review or guideline. Therefore, as stipulated in the study protocol we did not identify tertiary evidence source/s.<br>**We rated OCEBM level of evidence for this source as '3' (Non-Randomised controlled cohort/follow-up study) because:**<br>▪ Ronnigen and Kjeken (2008) is a non-randomised cohort study.

Dose parameter / Underpinning evidence	Type of strength exercise	Sets	Repetitions	Load	Intensity	Recovery	Method of progression	Frequency	Programme duration	Consistency rating
**Dulgeroglu et al (2016)**	1. Ulnar deviation of the fingers (with fingers flexed)<br>2. Flexing the fingers into a fist,<br>3. Extending the fingers<br>4. Touching the tip of each finger with the thumb<br>5. Rolling a "ball" with the palm on the table<br>6. Radial finger walking with the four ulnar fingers moving towards the thumb, Abduction of the thumb with the IP joint flexed.	Insufficiently described	3 repetitions	Soft putty	Insufficiently described	Insufficiently described	Insufficiently described	2 x daily	10 days	Exercise type: n/a<br>Sets: n/a<br>Repetitions: Inconsistent<br>Load: n/a<br>Intensity: n/a<br>Recovery: n/a<br>Progression: n/a<br>Frequency: n/a<br>Duration: n/a
**Pelland et al (2002)**	Citation not used to support parameter	Citation not used to support parameter	Unclear	Citation not used to support parameter	Citation not used to support parameter	Citation not used to support parameter	Citation not used to support parameter	Citation not used to support parameter	Citation not used to support parameter	

Dose parameter / Underpinning evidence	Type of strength exercise	Sets	Repetitions	Load	Intensity	Recovery	Method of progression	Frequency	Programme duration	Consistency rating
**Dulgeroglu et al (2016)**	1. Ulnar deviation of the fingers (with fingers flexed)<br>2. Flexing the fingers into a fist,<br>3. Extending the fingers<br>4. Touching the tip of each finger with the thumb<br>5. Rolling a "ball" with the palm on the table<br>6. Radial finger walking with the four ulnar fingers moving towards the thumb,<br>7. Abduction of the thumb with the IP joint flexed.	Insufficiently described	3 repetitions	Soft putty	Insufficiently described	Insufficiently described	Insufficiently described	2 x daily	10 days	Exercise type: n/a<br><br>Sets: n/a<br><br>Repetitions: Consistent<br><br>Load: n/a<br><br>Intensity: n/a<br><br>Recovery: n/a<br><br>Progression: n/a<br><br>Frequency: Inconsistent<br><br>Duration: Inconsistent
**Ronnigen and Kjeken et al (2008)**	Citation not used to support parameter	Citation not used to support parameter	3 repetitions	Citation not used to support parameter	Citation not used to support parameter	Citation not used to support parameter	Citation not used to support parameter	Participants in the conservative hand exercise programme instructed to exercise to usual training regimes	14 weeks	

Lourenzi et al (2017)

Slides 52-57

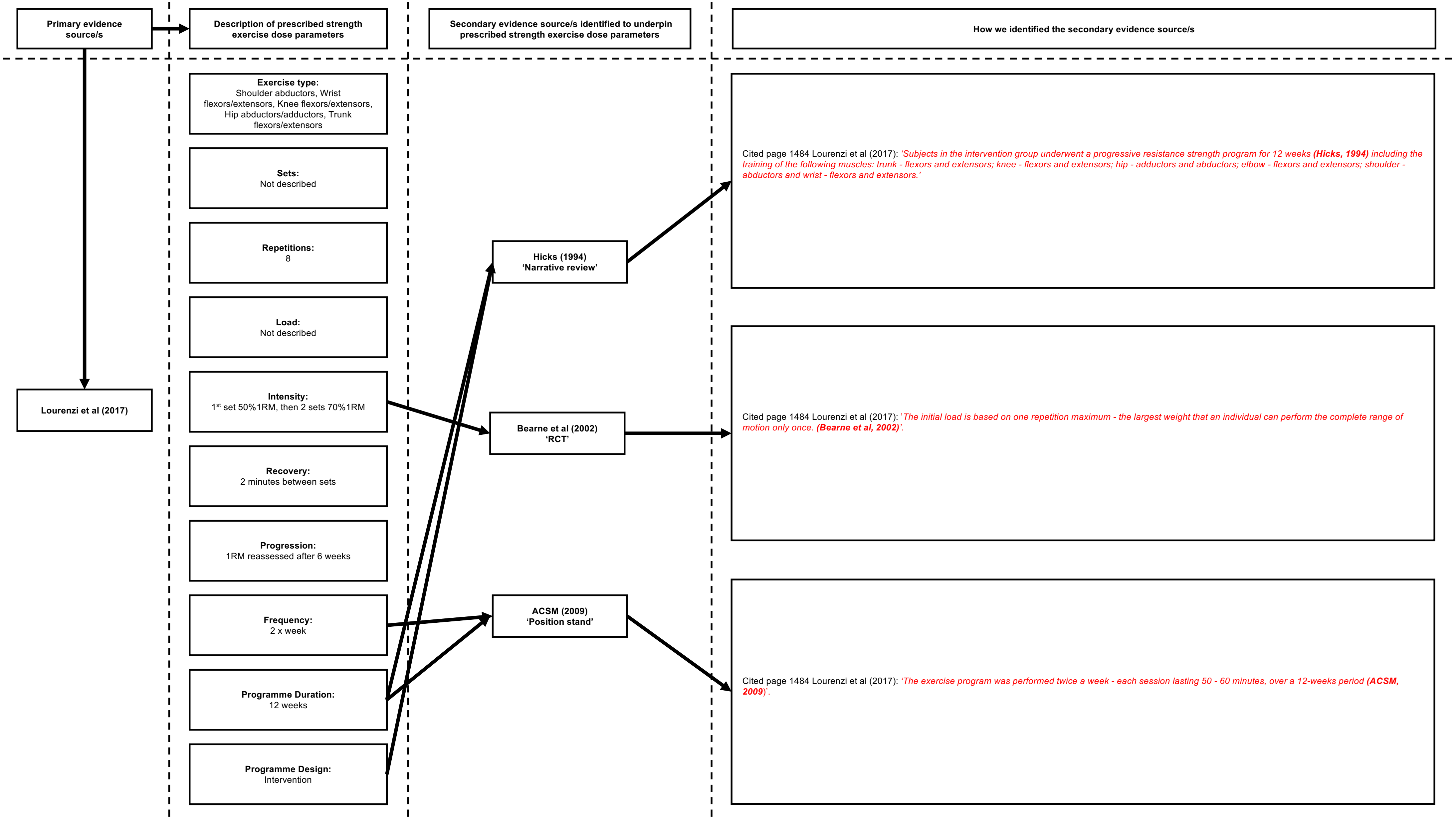
Primary evidence source/s
Description of prescribed strength exercise dose parameters
Secondary evidence source/s identified to underpin prescribed strength exercise dose parameters
How we identified the secondary evidence source/s
Lourenzi et al (2017)
Exercise type: Shoulder abductors, Wrist flexors/extensors, Knee flexors/extensors, Hip abductors/adductors, Trunk flexors/extensors
Sets: Not described
Repetitions: 8
Load: Not described
Intensity: 1st set 50%1RM, then 2 sets 70%1RM
Recovery: 2 minutes between sets
Progression: 1RM reassessed after 6 weeks
Frequency: 2 x week
Programme Duration: 12 weeks
Programme Design: Intervention
Hicks (1994) 'Narrative review'
Bearne et al (2002) 'RCT'
ACSM (2009) 'Position stand'
Cited page 1484 Lourenzi et al (2017): *'Subjects in the intervention group underwent a progressive resistance strength program for 12 weeks **(Hicks, 1994)** including the training of the following muscles: trunk - flexors and extensors; knee - flexors and extensors; hip - adductors and abductors; elbow - flexors and extensors; shoulder - abductors and wrist - flexors and extensors.'*
Cited page 1484 Lourenzi et al (2017): *'The initial load is based on one repetition maximum - the largest weight that an individual can perform the complete range of motion only once. **(Bearne et al, 2002)**'.*
Cited page 1484 Lourenzi et al (2017): *'The exercise program was performed twice a week - each session lasting 50 - 60 minutes, over a 12-weeks period **(ACSM, 2009)**'.*

Secondary evidence source/s identified to underpin prescribed strength exercise dose parameters	Oxford centre for evidence based medicine 2011 levels of evidence (OCEBM)	How we rated OCEBM level of evidence for the secondary evidence source/s
**Hicks (1994)**<br>**'Narrative review'**	**OCEBM level**<br>**'2-5'**	**Key information:**<br>▪ Hicks (1994) was identified as a literature review we attempted to identify tertiary evidence source/s most appropriate to underpinning the identified prescription parameter/s it was used to support. In view that Lourenzi et al (2017) used Hicks (1994) to underpin programme design using progressive resistance training and programme duration, we searched Hicks (1994) for tertiary evidence supporting these parameters. We refined our search by focusing on evidence underpinning dynamic strength exercise owing to Lourenzi et al (2017) using dynamic strength exercise in their intervention. It is most likely Lourenzi et al (2017) used sections from Hicks (1994) describing isotonic and isokinetic exercise located on pages 711 -714 and a section describing hand and wrist on page 722 (Hicks, 1994). We cautiously identified 12 citations underpinning progressive resistance training. These included 3 randomised controlled trials, 2 cross-sectional studies and 1 narrative review. We were not able to obtain the remaining 6 citations to be able to gather further information.<br><br>**We rated OCEBM level of evidence for this source '2-5' because:**<br>▪ The tertiary evidence sources identified as most likely underpinning the parameter ranged between these two levels of evidence.
**Bearne et al (2002)**<br>**'RCT'**	**OCEBM level**<br>**'2'**	**Key information:**<br>▪ Bearne et al (2002) was not identified as a pilot study for the primary evidence source, literature review or guideline. Therefore, we did not identify tertiary evidence source/s.<br><br>**We rated OCEBM level of evidence for this source as '2' (Randomised trial or observational study with dramatic effect) because:**<br>▪ Bearne et al (2002) is a randomised controlled trial.
**ACSM (2009)**<br>**'Position stand'**	**OCEBM level**<br>**'2-4' for frequency**<br>**'Unclear for programme duration'**	**Key information**<br><br>▪ ACSM (2009) was identified as a guideline, therefore we attempted to identify tertiary evidence source/s most appropriate to underpinning the identified prescription parameter/s it was used to support. In view that Lourenzi et al (2017) used ACSM (2009) to underpin both frequency and programme duration, we searched ACSM (2009) for tertiary evidence supporting these parameters.<br><br>Parameter: Frequency<br>▪ It is most likely Lourenzi et al (2017) used Table 2. 'summary of progressive resistance training recommendations' located on page 700. However, this table does not directly link to the citations contained in the references section. However, tertiary evidence may be cautiously identified for underpinning frequency within the position stand evidence statement and recommendations (evidence categories A, B and C). We identified 31 possible citations relevant to underpinning frequency. These included 13 randomised controlled trials, 11 non-randomised trials, 4 cohort studies, 1 systematic review + meta-analysis, 1 narrative review and 1 unknown.<br><br>Parameter: Programme duration<br>▪ We were unable to identify tertiary evidence source/s underpinning programme duration.<br><br>**We rated OCEBM level of evidence for this source as '2-4' for frequency and 'unclear' for programme duration because:**<br>▪ The tertiary evidence sources identified as most likely underpinning the parameter 'frequency' ranged between these two levels of evidence and 'unclear' for the parameter 'programme duration' as we could locate underpinning tertiary evidence.

Dose parameter / Underpinning evidence	Type of strength exercise	Sets	Repetitions	Load	Intensity	Recovery	Method of progression	Frequency	Programme duration	Consistency rating
**Lourenzi et al (2017)**	1. Shoulder abductors<br>2. Wrist flexors/extensors<br>3. Knee flexors/extensors<br>4. Hip abductors/adductors<br>5. Trunk flexors/extensors	Insufficiently described	8 repetitions	Insufficiently described	1st set 50%1RM then 2 sets 70%1RM	2 minutes between sets	1RM reassessed after 6 weeks	2 x week	12 weeks	Exercise type: Unclear<br>Sets: Unclear<br>Repetitions: Inconsistent<br>Load: Unclear<br>Intensity: Unclear<br>Recovery: Inconsistent<br>Progression: Inconsistent<br>Frequency: Consistent
**Hicks (1994)**	1. Isometric<br>2. Isotonic<br>3. Isokinetic	Unclear	10 repetitions	1-2 pounds	Unclear	Isometric exercise 20 seconds rest between contractions<br>Isometric exercise Unclear	After 1 month of isometric exercises the patient can be advanced to an isotonic exercise program	The exercise program is done 3 x week for each muscle group to be exercised.	Programs usually require 12-weeks duration.	

Dose parameter<br>Underpinning evidence	Type of strength exercise	Sets	Repetitions	Load	Intensity	Recovery	Method of progression	Frequency	Programme duration	Consistency rating
**Lourenzi et al (2017)**	1. Shoulder abductors<br>2. Wrist flexors/extensors<br>3. Knee flexors/extensors<br>4. Hip abductors/adductors<br>5. Trunk flexors/extensors	Insufficiently described	8 repetitions	Insufficiently described	1st set 50%1RM then 2 sets 70%1RM	2 minutes between sets	1RM reassessed after 6 weeks	2 x week	12 weeks	Exercise type: n/a<br>Sets: n/a<br>Repetitions: n/a<br>Load: n/a<br>Intensity: Inconsistent<br>Recovery: n/a<br>Progression: n/a<br>Frequency: n/a<br>Duration: n/a
**Bearne et al (2002)**	Citation not used to support parameter	Citation not used to support parameter	Citation not used to support parameter	Citation not used to support parameter	Isometric MVC Functional 1-5 minutes recording number of repetitions	Citation not used to support parameter	Citation not used to support parameter	Citation not used to support parameter	Citation not used to support parameter	

Dose parameter / Underpinning evidence	Type of strength exercise	Sets	Repetitions	Load	Intensity	Recovery	Method of progression	Frequency	Programme duration	Consistency rating
**Lourenzi et al (2017)**	1. Shoulder abductors<br>2. Wrist flexors/extensors<br>3. Knee flexors/extensors<br>4. Hip abductors/adductors<br>5. Trunk flexors/extensors	Insufficiently described	8 repetitions	Insufficiently described	1st set 50%1RM then 2 sets 70%1RM	2 minutes between sets	1RM reassessed after 6 weeks	2 x week	12 weeks	Exercise type: n/a<br>Sets: n/a<br>Repetitions: n/a<br>Load: n/a<br>Intensity: n/a<br>Recovery: n/a<br>Progression: n/a<br>Frequency: Consistent<br>Duration: Unclear
**ACSM (2009)**	Citation not used to support parameter	Citation not used to support parameter	Citation not used to support parameter	Citation not used to support parameter	Citation not used to support parameter	Citation not used to support parameter	Citation not used to support parameter	Novice 2 -3 x week<br>Intermediate 3-4 x week<br>Advanced 4-6 x week	Unclear	

Piva et al (2018)

Slides 58-63

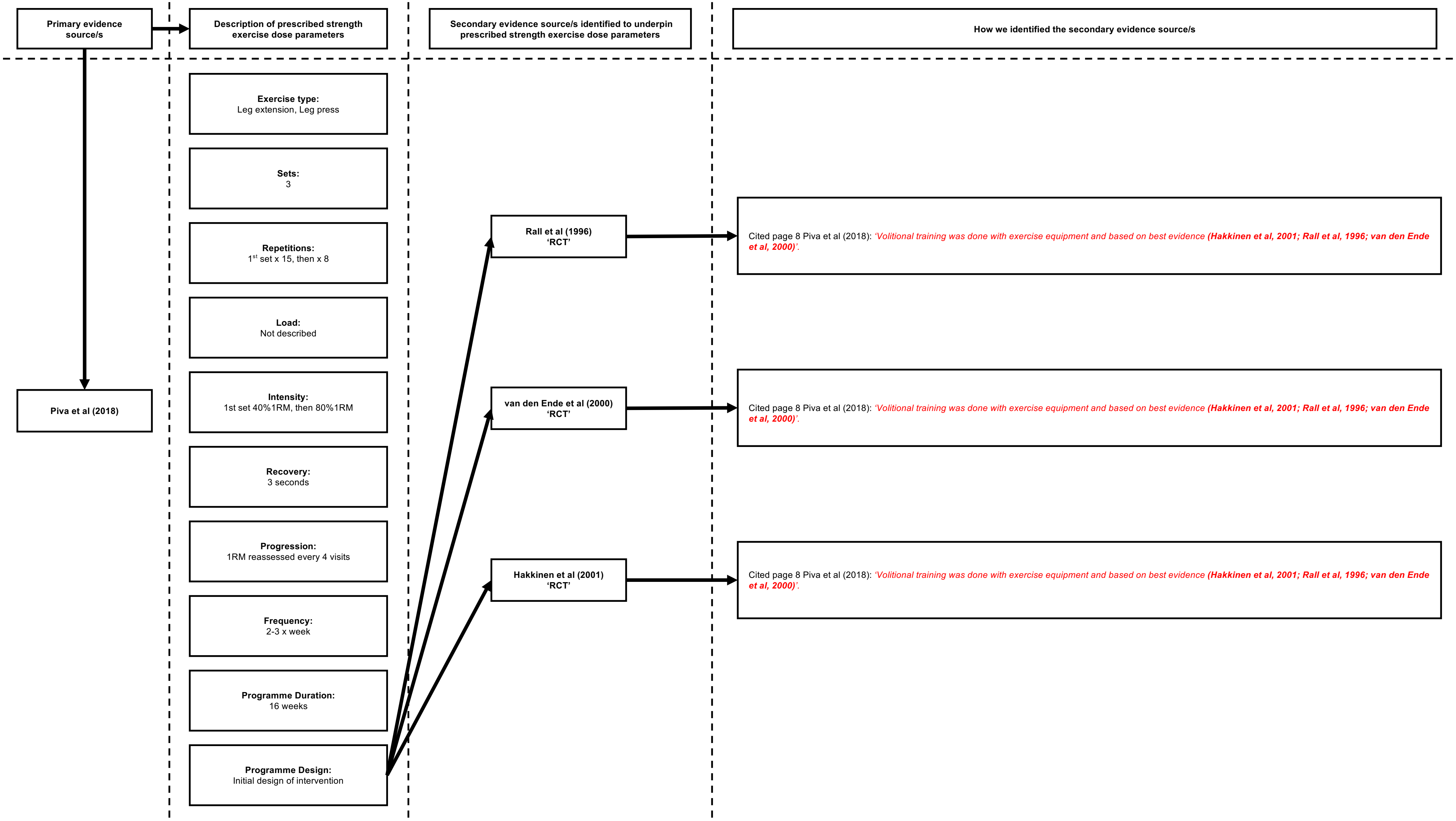
Primary evidence source/s
Description of prescribed strength exercise dose parameters
Secondary evidence source/s identified to underpin prescribed strength exercise dose parameters
How we identified the secondary evidence source/s
Piva et al (2018)
Exercise type: Leg extension, Leg press
Sets: 3
Repetitions: 1st set x 15, then x 8
Load: Not described
Intensity: 1st set 40%1RM, then 80%1RM
Recovery: 3 seconds
Progression: 1RM reassessed every 4 visits
Frequency: 2-3 x week
Programme Duration: 16 weeks
Programme Design: Initial design of intervention
Rall et al (1996) 'RCT'
van den Ende et al (2000) 'RCT'
Hakkinen et al (2001) 'RCT'
Cited page 8 Piva et al (2018): 'Volitional training was done with exercise equipment and based on best evidence (Hakkinen et al, 2001; Rall et al, 1996; van den Ende et al, 2000)'.
Cited page 8 Piva et al (2018): 'Volitional training was done with exercise equipment and based on best evidence (Hakkinen et al, 2001; Rall et al, 1996; van den Ende et al, 2000)'.
Cited page 8 Piva et al (2018): 'Volitional training was done with exercise equipment and based on best evidence (Hakkinen et al, 2001; Rall et al, 1996; van den Ende et al, 2000)'.

Secondary evidence source/s identified to underpin prescribed strength exercise dose parameters	Oxford centre for evidence based medicine 2011 levels of evidence (OCEBM)	How we rated OCEBM level of evidence for the secondary evidence source/s
**Rall et al (1996)** **'RCT'**	**OCEBM level** **'2'**	**Key information:** ▪ Rall et al (1996) was not identified as a pilot study for the primary evidence source, literature review or guideline. Therefore, we did not identify tertiary evidence source/s. **We rated OCEBM level of evidence for this source as '2' (Randomised trial or observational study with dramatic effect) because:** ▪ Rall et al (1996) is a randomised controlled trial.
**van den Ende et al (2000)** **'RCT'**	**OCEBM level** **'2'**	**Key information:** ▪ van den Ende et al (2001)) was not identified as a pilot study for the primary evidence source, literature review or guideline. Therefore, we did not identify tertiary evidence source/s. **We rated OCEBM level of evidence for this source as '2' (Randomised trial or observational study with dramatic effect) because:** ▪ van den Ende et al (2001) is a randomised controlled trial.
**Hakkinen et al (2001)** **'RCT'**	**OCEBM level** **'2'**	**Key information:** ▪ Hakkinen et al (2001) was not identified as a pilot study for the primary evidence source, literature review or guideline. Therefore, we did not identify tertiary evidence source/s. **We rated OCEBM level of evidence for this source as '2' (Randomised trial or observational study with dramatic effect) because:** ▪ Hakkinen et al (2001) is a randomised controlled trial.

Dose parameter<br>Underpinning evidence	Type of strength exercise	Sets	Repetitions	Load	Intensity	Recovery	Method of progression	Frequency	Programme duration	Consistency rating
**Piva et al (2018)**	1. Leg extension<br>2. Leg press	3 sets	1st set<br>15 repetitions<br><br>2nd and 3rd sets<br>8 repetitions	Insufficiently described	1st set<br>40%1RM<br>then<br>80%1RM	3 seconds	1RM reassessed every 4 visits	2-3 x week	16 weeks	Exercise type:<br>Consistent<br><br>Sets:<br>Consistent<br><br>Repetitions:<br>Inconsistent<br><br>Load:<br>Unclear<br><br>Intensity:<br>Inconsistent<br><br>Recovery:<br>Inconsistent<br><br>Progression:<br>Inconsistent<br><br>Frequency:<br>Inconsistent<br><br>Duration:<br>Inconsistent
**Rall et al (1996)**	Major muscle groups using chest press, leg press, leg extension, back extension and abdominal curl machines.	3 sets	8 repetitions	Unclear	80%1RM	2-3 seconds rest between repetitions and 2 minutes rest between sets.<br><br>Training sessions separated by 2-3 days rest	Strength testing performed at baseline and every 2 weeks to maintain a constant training intensity of 80% as strength improved.	2 x week	12 weeks	

Dose parameter<br>Underpinning evidence	Type of strength exercise	Sets	Repetitions	Load	Intensity	Recovery	Method of progression	Frequency	Programme duration	Consistency rating
**Piva et al (2018)**	1. Leg extension<br>2. Leg press	3 sets	1st set<br>15 repetitions<br><br>2nd and 3rd sets<br>8 repetitions	Insufficiently described	1st set<br>40%1RM<br>then<br>80%1RM	3 seconds	1RM reassessed every 4 visits	2-3 x week	16 weeks	Exercise type: Inconsistent<br><br>Sets: Consistent<br><br>Repetitions: Inconsistent<br><br>Load: Unclear<br><br>Intensity: Inconsistent<br><br>Recovery: Inconsistent<br><br>Progression: Inconsistent<br><br>Frequency: Inconsistent<br><br>Duration: Inconsistent
**van den Ende et al (2000)**	1. Isometric knee extension<br>2. Isometric knee flexion<br>3. Isometric shoulder girdle muscles<br>4. Isometric larger joints<br>5. Isokinetic knee extension<br>6. Isokinetic knee flexion	Isometric:<br>3 sets<br><br>Isokinetic:<br>3 sets<br><br>Isometric shoulder:<br>1 set	Isometric:<br>5 repetitions<br><br>Isokinetic:<br>8 repetitions<br><br>Isometric shoulder:<br>6 repetitions	Unclear	Isometric: 70% MVC for 6 seconds at 45° flexion<br><br>Isokinetic: 70% MVC at angular velocity 60°/s<br><br>Isometric shoulder: 6 seconds	Isometric: 30 seconds between sets<br><br>Isokinetic: unclear<br><br>Isometric: unclear	MVC determined every week by exercise therapist	5 x week	24 weeks	

<table>
<tr><th>Dose parameter
Underpinning evidence</th><th>Type of strength exercise</th><th>Sets</th><th>Repetitions</th><th>Load</th><th>Intensity</th><th>Recovery</th><th>Method of progression</th><th>Frequency</th><th>Programme duration</th><th>Consistency rating</th></tr>
<tr><td>Piva
et al
(2018)</td><td>1. Leg extension
2. Leg press</td><td>3 sets</td><td>1st set
15 repetitions
2nd and 3rd sets
8 repetitions</td><td>Insufficiently described</td><td>1st set
40%1RM
then
80%1RM</td><td>3 seconds</td><td>1RM reassessed every 4 visits</td><td>2-3 x week</td><td>16 weeks</td><td rowspan="2">Exercise type:
Unclear
Sets:
Inconsistent
Repetitions:
Inconsistent
Load:
Unclear
Intensity:
Inconsistent
Recovery:
Unclear
Progression:
Inconsistent
Frequency:
Inconsistent
Duration:
Inconsistent</td></tr>
<tr><td>Hakkinen
et al
(2001)</td><td>1. Upper limb exercises
2. Lower limb exercises
3. Abdominal exercises
4. Back exercises</td><td>2 sets</td><td>8-12 repetitions</td><td>Upper limb:
Elastic bands
Lower limb:
Elastic bands
Abdominal:
Dumbbells
Back:
Dumbbells</td><td>50-70%1RM</td><td>Insufficiently described</td><td>Intensity of strength training re-evaluated every 6 months</td><td>2 x week</td><td>24 months</td></tr>
</table>
