# Supplementary material for: A systematic review exploring the evidence reported to underpin exercise dose in clinical trials of rheumatoid arthritis
Source: Rheumatology (Oxford). 2020 Aug 11;59(11):3147–57. doi: 10.1093/rheumatology/keaa150 (PMC7590408; doi:10.1093/rheumatology/keaa150)
Supplement: keaa150_supplementary_data [file keaa150_supplementary_data.zip › Supplementary table S1_GB05082020docx.docx]

Characteristics of included primary evidence sources (presented in chronological order of publication).

| **RCT**  Author and year | **Country of origin** | **Primary aim** | **Sample characteristics** | | **Intervention content** | |
| --- | --- | --- | --- | --- | --- | --- |
|  |  |  | **Control**  **group** | **Intervention**  **group** | **Control group** | **Intervention group** |
|  | |  |  |  |  |  |
| **van den Ende**  **et al**  **(2000)**  ^[40]^ | Netherlands | To examine the consequences of an intensive exercise regimen on disease activity in active RA. | n=30  Mean age:  58 ± 14  Male/female:  41%/59% | n=34  Mean age:  62 ± 13  Male/female:  41%/59% | Conservative exercise programme that included range of movement (ROM) exercises for hands and feet and isometric exercises for the larger joints.  Strength exercises used:   1. Isometric exercises of the larger joints. | Control intervention plus a dynamic, intensive exercise regime of isometric and isokinetic exercises and cycling using a home trainer.  Strength exercises used:   1. Isometric shoulder girdle in prone position. 2. Isometric knee extensor/flexor. 3. Isokinetic knee extensor/flexor.   Duration: Unclearly reported.  Follow-up: 24 weeks |
| **Buljina**  **et al**  **(2001)**  ^[41]^ | Bosnia and Herzegovina | To study the short-term effects of physical therapy (ice massage or wax packs, thermal baths, and faradic hand baths) and exercise therapy on the rheumatoid hand. | n=50  Mean age:  48.46 ± 10.65  Male/female:  26%/74% | n=50  Mean age:  47.94 ± 11.22  Male/female:  24%/76% | Waiting list control – participants waited 4 weeks until enrolment into physical and exercise therapy programme. | Physical and exercise therapy programme including thermal baths, therapeutic heat or cold, faradic hand baths and wax bath treatment.  Strength exercises used:   1. Finger abduction. 2. Finger adduction. 3. Gross grip.   Duration: 3 weeks.  Follow-up: 3 weeks |
| **Hakkinen**  **et al**  **(2001)**  ^[42]^ | Finland | To investigate whether the 24-month strength training program used to increase muscle strength and physical function in patients with early RA also produces positive effects on bone mineral density in these patients. | n=31  Mean age:  49 ± 11  Male/female:  35%/65% | n=31  Mean age:  49 ± 10  Male/female:  42%/58% | ROM and stretching exercises and free to continue recreational activities with the exception of strength training of any kind. | Dynamic strength training.  Strength exercises used:   1. Exercises for upper and lower extremities using resistance bands. 2. Abdominal and back exercises using dumbbells.   Duration: 24 months.  Follow-up: 24 months. |
| **Bearne**  **et al**  **(2002)**  ^[43]^ | United Kingdom | To compare quadriceps sensorimotor function, lower limb functional performance and disability in patients with RA and healthy subjects, and to investigate the efficacy and safety of a brief rehabilitation regime. | Waiting list control group:  n=46  Mean age:  59.5 (range: 30–82)  Male/female:  Unclear  Healthy subjects  (comparative group)  n=25  Mean age:  65.5 (range: 50–82)  Male/female:  36%/64% | n=47  Mean age:  59.5 (range: 30 - 82)  Male/female:  Unclear | Waiting list control - participants waited 8 weeks before being invited to take part in the progressive exercise programme. | Progressive, individually tailored exercise programme including strength, functional and balance exercise.  Strength exercises used:   1. Isometric quadriceps. 2. Functional exercises (e.g. sit to stand, step-up’s etc.).   Duration: 10 weeks.  Follow-up: 12 months. |
| **De Jong**  **et al**  **(2003)**  ^[44]^ | Netherlands | To compare the effectiveness and safety of a long-term intensive exercise program with those of physical therapy (usual care) | n=158  Mean age:  53.5 (18) IQR  Male/female:  21%/79% | n=151  Mean age:  54.0 (16) IQR  Male/female:  21%/79% | Only treated by a physical therapist if this was regarded as necessary by their attending physician. | Rheumatoid Arthritis Patients In Training (RAPIT Programme). Each session included warm-up, bicycle training, exercise circuit, sport or game and cool-down.  Strength exercises used:  Unclear  Duration: 24 months.  Follow-up: 24 months. |
| **Veitiene**  **and Tamulaitiene (2004)**  ^[45]^ | Lithuania | To compare the efficiency of home and outpatient exercise program in patients with RA. | n=21  Mean age:  59.8 ± 11.1  Male/female:  5%/95% | n=10  Mean age:  64.4 ± 9.4  Male/female:  0%/100% | ROM and isometric strength exercise programme supervised in the outpatient department.  Strength exercises used:   1. Sit-up exercises on the back 2. Isometric exercises | ROM and isometric strength exercise programme conducted at home.  Duration: 3 months.  Follow-up: 3 months. |
| **O’Brien**  **et al**  **(2006)**  ^[39]^ | United Kingdom | To evaluate the clinical effectiveness of three different hand therapy approaches (two of which employed different hand exercise regimes) on changes in impairment and activity limitation in patients with RA over a 6-month period. | n=103  Mean age:  65 ± 8  Male/female: 21%79% | Group 1:  n=21  Group 2:  n=24  Mean age:  65 ± 7  Male/female: unclear  (reported 25%/75% for group 1 + 2) | Joint protection literature covering the basic principles of joint protection, energy conservation, ‘top tips’ relating to personal and household activities, postural advice, types of splinting and issues related to sexuality. | Group 1: Control and eight strengthening and mobilising exercises.  Strength exercises used:   1. Pinch grip exercises 2. Strengthening the intrinsic/thenar eminence muscles (using a towel). 3. Wrist extension with Theratubes band.   Duration: 6 months.  Follow-up: 6 months.  Group 2: Control and eight stretching exercises, without any specific strengthening exercises.  Duration: 6 months.  Follow-up: 6 months. |
| **van den Berg**  **et al**  **(2006)**  ^[46]^ | **Country:**  Netherlands | To compare the effectiveness of two internet-based physical activity interventions for patients with rheumatoid arthritis. | n=78  Mean age:  49.8 (13.9) median (IQR) years  (Mean age: + SD not reported)  Male/female:  23%/77% | n=82  Mean Age: 49.5 (12.9) median (IQR) years  (Mean Age: + SD not reported)  Male/female:  24%/76% | Access to web pages where general information about aerobic, muscle strengthening, and range of movement exercises and the promotion of physical activity in patients with RA was presented. | Access to web pages where individualised training intervention consisting of muscle strengthening exercises, ROM exercise and cycling on a bicycle ergometer was presented.  Strength exercises used:  Not described.  Duration: 12 months.  Follow-up: 12 months. |
| **Eversden**  **et al**  **(2007)**  ^[47]^ | United Kingdom | To compare individualised exercises whilst immersed in a heated pool to similar exercises on land for their effect on overall improvement in health, physical function and quality of life in people with RA. | n=58  Age:  56.1 ± 11.9  Male/female:  32%/68% | n=57  Age:  55.2 ± 13.3  Male/female:  28%/72% | Land based exercise including mobilising and stretching, joint mobility, muscle strength and functional activities.  Strength exercises used:  Not described.  Duration: 6 weeks.  Follow-up: 3 months. | Hydrotherapy exercises including mobilising and stretching, joint mobility, muscle strength and functional activities. |
| **Neuberger**  **et al**  **(2007)**  ^[59]^ | United States of America | To determine the effects of participation in a low-impact aerobic exercise program on fatigue, pain, and depression; to examine whether intervention groups compared with a control group differed on functional (grip strength and walk time) and disease activity (total joint count, erythrocyte sedimentation rate, and C-reactive protein) measures and aerobic fitness at the end of the intervention; and to test which factors predicted exercise participation. | n=105  Mean age:  (entire sample  55.5 years (range 40–70 years))  Male/female:  Unclear  (entire sample  82.7% were women) | Class exercise group  n=102  Mean age:  Not described  (Entire sample  55.5 years (range 40–70 years))  Male/female:  Not described (entire sample  82.7% were women)  Home exercise group  n=103  Mean age:  Not described  (entire sample  55.5 years (range 40–70 years))  Male/female:  Not described (entire sample  82.7% were women) | Asked to keep exercise levels at baseline amounts. | Class exercise group:  The exercises were performed at a fitness centre and consisted of 4 phases: warm-up, low-impact aerobics, strengthening, and cool-down exercises.  Strength exercises used:  Unclear.  Duration: 12 weeks.  Follow-up: 12 weeks.  Home exercise group:  The exercises were performed at home using a video recording and  consisted of 4 phases: warm-up, low-impact aerobics, strengthening, and cool-down exercises.  Strength exercises used:  Unclear.  Duration: 12 weeks.  Follow-up: 12 weeks. |
| **Flint-Wagner**  **et al**  **(2009)**  ^[60]^ | United States of America | To investigate the effects of a 16-week, high-intensity, individualized, strength training program in infliximab-treated RA patients. | n=8  Mean age:  49.0 ± 12.6  Male/female:  Not described | n=16  Mean age:  52.2 ± 13  Male/female:  Not described | Continued with care overseen by their rheumatologists | Exercise programme: included a walking warm-up, strength training, aerobic exercise, abdominal exercises, and a cool-down period with walking and static stretching.  Strength exercises used:   1. Incline press. 2. Row. 3. Hammer curl. 4. Leg press. 5. Leg curl. 6. Hip abduction. 7. Hip adduction. 8. Calf raise.   Duration: 16 weeks.  Follow-up: 16 weeks. |
| **Lemmey**  **et al**  **(2009)**  ^[48]^ | United Kingdom | To confirm preliminary observations (i.e. that progressive resistance training reverses debilitating cachexia and improves function in patients with RA) and to investigate the role of the local IGF system in exercise-induced hypertrophy of skeletal muscle in patients with RA. | n=18  Mean age:  60.6 ± 11.2  Male/female:  16%/84% | n=18  Mean age:  55.6 ± 8.3  Male/female:  20%/80% | Home ROM exercises. | Progressive resistance training + low intensity ROM exercises.  Strength exercises used:   1. Chest press. 2. Rowing. 3. Bicep curl. 4. Triceps extension. 5. Seated leg extension. 6. Leg press. 7. Leg curl. 8. Standing calf raises.   Duration: 24 weeks.  Follow-up: 24 weeks. |
| **van Rensburg**  **et al**  **(2010)**  ^[66]^ | South Africa | To measure the effect of an endurance training programme on the fitness parameters, quality of life and disease activity of females suffering from RA. | n=12  Mean age:  49.7 ± 4.3  Male/female:  0%/100% | n=25  Mean age:  47.3 ± 9.2  Male/female:  0%/100% | Continue with their sedentary lifestyles. | Land exercise group:  Warm-up phase, aerobic exercise, strength training, and flexibility training.  Strength exercises used:  Unclear.  Duration: 12 weeks.  Follow-up: 12 weeks.  Aquatic exercise group:  Exercise programme consisting of a warm-up phase, aerobic exercise, strength training, and flexibility training. |
| **Breedland**  **et al**  **(2011)**  ^[49]^ | Netherlands | To evaluate the effects of a group-based exercise and educational program on the physical performance and disease self- management of people with RA. | n=15  Mean age:  51.8 ± 9.4  Male/female:  37%/63% | n=19  Mean age:  45 ± 11.9  Male/female:  20%/80% | Waiting list control | The FIT programme: 8-week, multidisciplinary group therapy program, consisting of physical exercise designed to increase aerobic capacity and muscle strength (force-generating capacity) with an educational programme to improve health status and self-efficacy for disease-self-management.  Strength exercises used:   1. Chest press. 2. En Tree pulley device. 3. Rowing. 4. Leg press. 5. Leg extension. 6. Leg curl. 7. Abdominal trainer. 8. Back trainer.   Duration: 8 weeks.  Follow-up: 22 weeks. |
| **Strasser**  **et al**  **(2011)**  ^[50]^ | Austria | To evaluate the effects of 6 months of combined strength and endurance training on: (1) the disease activity and functional ability in patients with RA and (2) the muscle strength, cardio-respiratory fitness, and anthropometry parameters in RA patients. | n=20  Mean age:  55.6 ± 9.7  Male/female:  15%/85% | n=20  Mean age:  59.3±7.9  Male/female:  5%/95% | Stretching exercises + normal recreational activities (except strength and endurance) | Strength training programme:  Exercises for major muscle groups. Endurance training was performed on a cycle ergometer.  Strength exercises used:   1. Bench press (pectoralis). 2. Chest cross (horizontal shoulder flexion). 3. Shoulder press (trapezius). 4. Pull downs (latissimus dorsi). 5. Bicep curls. 6. Triceps extension. 7. Leg press (quadriceps femoris). 8. Abdominal exercises. |
| **Rahnama**  **et al**  **(2012)**  ^[62]^ | Iran | To investigate the effects of two types of rehabilitation techniques, including aerobic and strengthening exercises on patients with knee rheumatoid arthritis. | n=16  Mean age:  59.6 ± 8.3  Male/female:  100%/0% | Strength  group  n=16  Mean age:  57.0 ± 7.4  Male/female:  100%/0%  Aerobic  group  n=16  Mean age:  59.4 ± 8.1  Male/female:  0%/100% | Beseeched to follow their ordinary life style. | Strength Group:  Progressive strength exercise  Strength exercises used:   1. Quadriceps. 2. Hamstrings.   Duration: 6 months.  Follow-up: 6 months.  Aerobic group:   1. Each subject ran on the treadmill for about 30 minutes, while the speed was set according to the patient ability. |
| **van Rensburg**  **et al**  **(2012)**  ^[65]^ | South Africa | The aim of the current study was to evaluate the effect of exercise on cardiac autonomic function as measured by short-term heart rate variability in RA patients. | n=22  Mean age:  47.08 ± 7.05  Male/female:  0%/100% | n=24  Mean age:  46.81 ± 9.23  Male/female:  0%/100% | Continue with their sedentary lifestyle | Exercise intervention:  Warm-up exercises, strengthening exercises, aerobic exercises and a cool down period which included stretching.  Strength exercises used:   1. Chest press. 2. Bicep curls. 3. Lateral pull-downs. 4. Hip extension. 5. Leg press. 6. Hamstring curls. 7. Hip abduction.   Duration: 12 weeks.  Follow-up: 12 weeks. |
| **Cima**  **et al**  **(2013)**  ^[67]^ | Brazil | To evaluate the effects of an exercise programme aimed at improving the force of intrinsic and extrinsic hand muscles of individuals who have RA hand deformities as well as to analyse the impact of these exercises on hand functionality. | n=7  Mean age:  60.4 ±7.4 years  Male/female:  0%/100% | n=13  Mean age:  53 ±10 years  Male/female:  0%/100% | No exercise for wrist and hand muscles. | Rehabilitation programme:  Consisted of exercises to strengthen the intrinsic and extrinsic muscles of the hands.  Strength exercises used:   1. Digiflex hand exerciser. 2. Flexed fingers squeezing the modelling mass. 3. Co-ordination movement of the flexo-extension of the fingers with the modelling mass. 4. Exercises for the intrinsic muscles with the modelling mass. 5. Pulp to pulp finger pinch performed with all fingers pulling an elastic band. 6. Exercises for hand intrinsic muscles with elastic. 7. Exercises for the hand intrinsic muscles with the modelling mass.   Duration: 10 weeks.  Follow-up: 10 weeks. |
| **Dogu**  **et al**  **(2013)**  ^[51]^ | Turkey | To evaluate the effect of 6-week-long isotonic and isometric hand exercises on pain, hand functions, dexterity and quality of life in women diagnosed as RA. The secondary objective of our work was to evaluate the effects of both exercise types on handgrip strength and disease activity | n=24  Age:  50.38 ± 9.32 years  Male/female:  0%/100% | n=23  Age:  54.91 ± 9.27 years  Male/female:  0%/100% | Isometric hand exercises and wax bath.  Strength exercises used:   1. Push hands by facing palms towards each other. 2. Ulnar deviation against pressure with fingers in flexion. 3. Pushing lid of the perfume bottle while interphalangeal (IP) joint of the thumb in flexion. 4. Abduction/adduction by placing the hands of the physician in between fingers. 5. Fingers at 90^°^ metacarpophalangeal (MCP) flexion, flexion and extension of the fingers against pressure. 6. Gripping a glass of water placed into the hand.   Duration: 6 weeks.  Follow-up: 6 weeks. | Isotonic hand exercises (ROM exercise) and wax bath. |
| **Durcan**  **et al**  **(2014)**  ^[52]^ | Ireland | To evaluate the effect of an exercise program on self-reported sleep quality and fatigue in RA. | n=38  Age:  59 ± 12 years  Male/female:  47%/53% | n=42  Age:  61 ± 8 years  Male/female:  25%/75% | The control group was composed of patients with RA who received advice only on the benefits of exercise in RA. | 12-week home exercise programme targeting deficiencies in strength, ROM and coordination and walking programme.  Strength exercises used:   1. Major muscle groups. 2. Functional exercises.   Duration: 12 weeks.  Follow-up: 12 weeks. |
| **Jahanbin**  **et al**  **(2014)**  ^[63]^ | Iran | To investigate the effects of conditioning exercises on the health status and pain in patients suffering from RA. | n=33  Mean age:  48.87 ± 9.24  Male/female:  Not described | n=32  Mean age:  48.6 ± 10.51  Male/female:  Not described | Not described | Physical training programme consisting of conditioning exercises included aerobic, isometric, and isotonic exercises.  Strength exercises used:   1. Isometric exercise. 2. Isotonic exercise.   Duration: 8 weeks.  Follow-up: 8 weeks. |
| **Manning**  **et al**  **(2014)**  ^[53]^ | United Kingdom | To evaluate the effectiveness of a brief supervised education, self-management, and global upper extremity exercise training program, supplementing a home exercise regimen, for people with RA; the Education, Self-Management, and Upper Extremity Exercise Training in People with Rheumatoid Arthritis (EXTRA) program). | n=56  Mean age:  57 ± 15)  Male/female:  32%/68% | n=56  Mean age:  53 ± 16  Male/female:  14%/86% | Usual care - continued to be managed by their medical team. | Education, Self-Management, and Upper Extremity Exercise Training for People with Rheumatoid Arthritis (EXTRA) program (4 group sessions and home programme).  Strength exercises used:  (6 out 16 exercises used)   1. Putty ball squeeze. 2. Putty fingertip pinch. 3. Putty finger hook and squeeze. 4. Knife and fork putty cutting. 5. Paper clips and envelope challenge. 6. Wrist alphabet with band. 7. Back scrub. 8. Up and out of chair. 9. Arm curl with band. 10. Lift to chin with band. 11. Reach back with band. 12. Side lift with band. 13. Wall wash squares with band. 14. Door push with band. 15. Shoulder rotation with band. 16. Reach to shelf with band.   Duration: 12 weeks.  Follow-up: 36 weeks. |
| **Lamb**  **et al**  **(2015)**  ^[54]^ | United Kingdom | To estimate, for people whose RA is controlled by various drug regimens, the effectiveness and cost-effectiveness of adding an individually tailored, progressive exercise programme for the hands and arms, in addition to best practice usual care. | n=244  Mean age:  63·5 ± 11  Male/female:  24%/76% | n=246  Mean age:  61·3 ± 12  Male/female:  24%/76% | Usual care (Joint protection, education and where indicated, functional splinting). | Usual care and exercise programme consisting of strengthening and ROM exercises.  Strength exercises used:   1. Eccentric wrist extension. 2. Gross grip. 3. Finger adduction. 4. Pinch grip.   Duration: 12 weeks.  Follow-up: 12 months. |
| **Seneca**  **et al**  **(2015)**  ^[55]^ | Denmark | To compare the effect of a partly supervised and a self-administered intensive exercise programme in patients with early RA. | n=26  Mean age:  61 (range 27-79)  Male/female:  31%/69% | n=25  Mean age:  61 (range 27-79)  Male/female:  32%/68% | Self-administered strength and aerobic exercise*.*  Strength exercises used:  Not described | Partly supervised strength and aerobic exercise*.*  Strength exercises used:   1. Shoulder. 2. Legs. 3. Trunk extensors/flexors.   Duration: 12 weeks.  Follow-up: 12 weeks. |
| **Dulgeroglu**  **et al**  **(2016)**  ^[56]^ | Turkey | To evaluate whether the galvanic electrotherapy can relieve rheumatic hand pain and whether conservative hand exercises increase the hand strength. | n=14  Median age: 51.5  (range 51 to 68 years)  Male/female:  0%/100% | n=16  Median age:  55 (range 50 to 75 years)  Male/female:  0%/100% | Home conservative exercise programme consisting of gentle exercises performed against resistance and ROM exercise.  Strength exercises used:   1. Ulnar deviation of the wrist (with fingers flexed). 2. Flexing the fingers into a fist. 3. Extending the fingers. 4. Touching the tip of each finger with the thumb. 5. Rolling a ‘ball’ with the palm on the table with fingers extended. 6. Radial finger walking with the four ulnar fingers moving towards the thumb. 7. Abduction of the thumb with the IP joint flexed.   Duration: 10 days.  Follow-up: 5 weeks. | Control and galvanic electrotherapy. |
| **Tonga**  **et al**  **(2016)**  ^[57]^ | Turkey | ^[58, 64, 69, 70]^This study aims to examine the effectiveness of client-centred occupational therapy in patients with rheumatoid arthritis (RA).  al therapy in patients with RA. | n=20  Age:  55.80±10.33  Male/female:  (2 males and 38 females took part in trial) | n=20  Age:  51.35±11.57  Male/female:  (2 males and 38 females took part in trial) | Ten sessions of physical therapy program were implemented on the control group consisting of pain management (hot-packs, cold-packs, and electrotherapy); exercises for stretching and strengthening; and educational therapy approaches (joint protection techniques, energy conservation techniques, splint and assistive devices use, etc.)  Strength exercises used:  Unclear.  Duration: Unclearly reported.  Follow-up: 4 weeks. | Control and 4 extra sessions delivering the Canadian Occupational Performance Measure. |
| **Lourenzi**  **et al**  **(2017)**  ^[68]^ | Brazil | To evaluate the effectiveness of an overall progressive resistance strength program involving muscles of upper/lower limbs and trunk, regarding physical functional, pain, health-related quality of life and muscle strength. | n=27  Mean age:  50.88 ± 8.57  Male/female:  9%/91% | n=33  Mean age:  52.63 ± 7.10  Male/female:  7%/93% | The control group were contacted by the principal investigator by telephone or email at least once a week in order to identify complications and to improve patient compliance. After finishing all evaluations, the patient had access to the progressive resistance strength program if desired. | Progressive resistance strength programme.  Strength exercises used:   1. Shoulder abductors. 2. Wrist extensors/flexors. 3. Elbow extensors/flexors. 4. Knee extensors/flexors. 5. Hip adductors/abductors. 6. Trunk extensors/flexors.   Duration: 12 weeks.  Follow-up: 24 weeks. |
| **Shinde**  **and Varadharajula**  **(2017)**  ^[69]^ | India | To study the effect of therapeutic exercise programme on adults with early RA” was conducted to determine effect of Therapeutic exercise programme on functional status, maximum grip strength & perceived health in adults with early RA. | n=15  Mean age:  Not described  Male/female:  Not described | n=15  Mean age:  Not described  Male/female:  Not described | Conventional therapy including general patient information, pain modulating modalities and prescriptions of hospital based training or home exercise programmes. | Group therapeutic exercise programme consisting of strength, aerobic and ROM exercises.  Strength exercises used:   1. Isometric exercises. 2. Dynamic resisted exercises for the major muscle groups.   Duration: 12 weeks.  Follow-up: 6 months. |
| **Anvar**  **et al**  **(2018)**  ^[64]^ | Iran | To determine the effectiveness of a self-management program amongst older women with RA. | n=40  Mean age:  69.03 ± 1.43  Male/female:  0%/100% | n=40  Mean age:  Unclear  Male/female:  0%/100% | Usual care | Arthritis self-management programme consisting of consisted of a stretching, endurance and light resistance exercises.  Strength exercises used:   1. Light resistance exercises.   Duration: 6 weeks.  Follow-up: 4 months. |
| **Lange**  **et al**  **(2018)**  ^[58]^ | Sweden | To evaluate the effect of a person-centred, moderate -to -high intensity, aerobic and resistance exercise protocol on older adults with RA. | n=38  Mean age:  70.11 ± 2.30  Male/female:  24%/76% | n=36  Mean age:  69.14 ± 2.61  Male/female:  25%/75% | One individual meeting with the physiotherapist where they were encouraged to perform home-based exercise according to the same protocol as the intervention group, but no gym-based exercise.  Strength exercises used:   1. Strength in the lower extremity. | Person-centred, supervised, exercise intervention consisting of aerobic and resistance exercise.  Strength exercises used:   1. Seated row. 2. Leg press. 3. Knee extension. 4. Bicep curl. 5. Core stability.   Duration: 20 weeks.  Follow-up: 12 months. |
| **Mohanty**  **et al**  **(2018)**  ^[70]^ | India | To compare the effect of Proprioceptive retraining technique over home exercise program on hand functions in RA. | Proprioceptive group  n=20  Mean age:  44.85 ± 7.35  Male/female:  20%/80% | Home exercise group  n=20  Mean age:  47.10 ± 6.98  Male/female:  15%/85% | Grip exercise activity, weighted pulley activity for fingers, lifting dumbbells with hand, wrist roller activity and stretch and hold of bilateral counterpart fingers.  Strength exercises used:   1. Grip exercise activity. 2. Weighted pulley activity for fingers 3. Lifting dumbbells with hand. | Simple movements of wrist, and finger joints, thumb movement performed against resistance, touching the base of each finger, volar and dorsal flexion of wrist, pronation and supination of forearm, and tendon gliding exercises.  Strength exercises used:   1. Simple movements of wrist, and finger joints, thumb movement performed against resistance. |
| **Piva**  **et al**  **(2018)**  ^[61]^ | United States of America | To compare the feasibility and effectiveness of neuromuscular electrical stimulation with high-intensity volitional resistance training in improving muscle structure and function , and physical function in patients with RA. | n=31  Mean age:  61.0 ± 11.0  Male/female:  18%/82% | n=28  Mean age:  57.2 ± 8.6  Male/female:  19%/81% | Volitional exercise consisting of leg extension and leg press using machines.  Strength exercises used:   1. Leg extension. 2. Leg press. | Neuromuscular electrical stimulation. |
