# Supplementary material for: A systematic review exploring the evidence reported to underpin exercise dose in clinical trials of rheumatoid arthritis
Source: Rheumatology (Oxford). 2020 Aug 11;59(11):3147–57. doi: 10.1093/rheumatology/keaa150 (PMC7590408; doi:10.1093/rheumatology/keaa150)
Supplement: keaa150_supplementary_data [file keaa150_supplementary_data.zip › Supplementary table S2_GB05082020docx.docx]

Summary of RoB for included primary evidence sources

|  | Sequence generation | Allocation concealment | Blinding of participants and personnel | Blinding of outcome assessors for all outcomes | Incomplete outcome data for all outcomes | Selective outcome reported | Other sources of bias |
| --- | --- | --- | --- | --- | --- | --- | --- |
| van den Ende et al 2000 ^[40]^ |  |  |  |  |  |  |  |
| Buljina et al 2001 ^[41]^ |  |  |  |  |  |  |  |
| Häkkinen et al 2001 ^[42]^ |  |  |  |  |  |  |  |
| Bearne et al 2002 ^[43]^ |  |  |  |  |  |  |  |
| de Jong et al 2003 ^[44]^ |  |  |  |  |  |  |  |
| Veitiene & Tamulaitiene 2004 ^[45]^ |  |  |  |  |  |  |  |
| O'Brien et al 2006 ^[39]^ |  |  |  |  |  |  |  |
| van den Berg et al 2006 ^[46]^ |  |  |  |  |  |  |  |
| Eversden et al 2007 ^[47]^ |  |  |  |  |  |  |  |
| Neuberger et al 2007 ^[59]^ |  |  |  |  |  |  |  |
| Flint-Wagner et al 2009 ^[60]^ |  |  |  |  |  |  |  |
| Lemmey et al 2009 ^[48]^ |  |  |  |  |  |  |  |
| van Rensburg et al 2010 ^[66]^ |  |  |  |  |  |  |  |
| Breedland et al 2011 ^[49]^ |  |  |  |  |  |  |  |
| Strasser et al 2011 ^[50]^ |  |  |  |  |  |  |  |
| Rahnama & Mazioum 2012 ^[62]^ |  |  |  |  |  |  |  |
| van Rensburg et al 2012 ^[65]^ |  |  |  |  |  |  |  |
| Cima et al 2013 ^[67]^ |  |  |  |  |  |  |  |
| Dogu et al 2013 ^[51]^ |  |  |  |  |  |  |  |
| Durcan et al 2014 ^[52]^ |  |  |  |  |  |  |  |
| Jahanbin et al 2014 ^[63]^ |  |  |  |  |  |  |  |
| Manning et al 2014 ^[53]^ |  |  |  |  |  |  |  |
| Lamb et al 2015 ^[54]^ |  |  |  |  |  |  |  |
| Seneca et al 2015 ^[55]^ |  |  |  |  |  |  |  |
| Dulgeroglu et al 2016 ^[56]^ |  |  |  |  |  |  |  |
| Tonga et al 2016 ^[57]^ |  |  |  |  |  |  |  |
| Lourenzi et al 2017 ^[68]^ |  |  |  |  |  |  |  |
| Shinde & Varadharajula 2017 ^[69]^ |  |  |  |  |  |  |  |
| Anvar et al 2018 ^[64]^ |  |  |  |  |  |  |  |
| Lange et al 2018 ^[58]^ |  |  |  |  |  |  |  |
| Mohanty et al 2018 ^[70]^ |  |  |  |  |  |  |  |
| Piva et al 2018 ^[61]^ |  |  |  |  |  |  |  |
