# Supplementary material for: A systematic review exploring the evidence reported to underpin exercise dose in clinical trials of rheumatoid arthritis
Source: Rheumatology (Oxford). 2020 Aug 11;59(11):3147–57. doi: 10.1093/rheumatology/keaa150 (PMC7590408; doi:10.1093/rheumatology/keaa150)
Supplement: keaa150_supplementary_data [file keaa150_supplementary_data.zip › Supplementary table S3_GB05082020.docx]

Adapted TIDieR checklist for reporting of interventions in included primary evidence sources.

| TIDieR Item | 1  Brief  Name | 2  Why | 3  What  Materials | 4  What  Procedures | 5  Who  Provided | 6  How  Delivered | 7  Where  Delivered | 8  When and How Much  (Strengthening Exercise) | | 9  Tailoring | 10  Modifications | 11  How well (planned) | 12  How well (actual) |
| --- | --- | --- | --- | --- | --- | --- | --- | --- | --- | --- | --- | --- | --- |
| van den Ende  et al (2000)^[40]^ | ✓ | ✓ | 🗶 | ✓ | ✓ | ✓ | ✓ | 1: 🗶  2: ✓  3: ✓  4: ✓  5: 🗶 | 6: ✓  7: ✓  8: ✓  9: ✓  10: ✓ | ✓ | 🗶 | 🗶 | ✓ |
| Buljina  et al (2001)^[41]^ | ✓ | ✓ | ✓ | ✓ | 🗶 | ✓ | ✓ | 1: ✓  2: ✓  3: 🗶  4: ✓  5: ✓ | 6: 🗶  7: ✓  8: 🗶  9: ✓  10: ✓ | 🗶 | 🗶 | 🗶 | 🗶 |
| Hakkinen  et al (2001)^[42]^ | ✓ | ✓ | 🗶 | ✓ | ✓ | ✓ | ✓ | 1: 🗶  2: ✓  3: ✓  4: ✓  5: 🗶 | 6: ✓  7: 🗶  8: ✓  9: ✓  10: ✓ | ✓ | 🗶 | 🗶 | ✓ |
| Bearne  et al (2002)^[43]^ | ✓ | ✓ | ✓ | ✓ | ✓ | ✓ | ✓ | 1: 🗶  2: ✓  3: 🗶  4: 🗶  5: 🗶 | 6: ✓  7: 🗶  8: ✓  9: ✓  10: ✓ | ✓ | ✓ | 🗶 | ✓ |
| De Jong  et al (2003)^[44]^ | ✓ | ✓ | 🗶 | ✓ | 🗶 | ✓ | 🗶 | 1: 🗶  2: 🗶  3: 🗶  4: ✓  5: 🗶 | 6: 🗶  7: ✓  8: ✓  9: ✓  10: ✓ | 🗶 | 🗶 | 🗶 | ✓ |
| Veitiene & Tamulaitiene  (2004)^[45]^ | ✓ | ✓ | 🗶 | 🗶 | 🗶 | ✓ | ✓ | 1: 🗶  2: 🗶  3: 🗶  4: 🗶  5: 🗶 | 6: 🗶  7: 🗶  8: 🗶  9: ✓  10: ✓ | 🗶 | 🗶 | 🗶 | 🗶 |
| O’Brien  et al (2006)^[39]^ | ✓ | ✓ | ✓ | ✓ | ✓ | ✓ | ✓ | 1: ✓  2: ✓  3: 🗶  4: ✓  5: 🗶 | 6: 🗶  7: 🗶  8: ✓  9: ✓  10: ✓ | ✓ | 🗶 | 🗶 | 🗶 |
| van den Berg  et al (2006)^[46]^ | ✓ | ✓ | ✓ | ✓ | ✓ | ✓ | ✓ | 1: 🗶  2: 🗶  3: ✓  4: ✓  5: 🗶 | 6: 🗶  7: ✓  8: 🗶  9: ✓  10: ✓ | ✓ | 🗶 | ✓ | ✓ |
| Eversden  et al (2007)^[47]^ | ✓ | ✓ | 🗶 | ✓ | ✓ | ✓ | ✓ | 1: 🗶  2: 🗶  3: 🗶  4: 🗶  5: 🗶 | 6: 🗶  7: 🗶  8: ✓  9: ✓  10: ✓ | ✓ | ✓ | 🗶 | ✓ |
| Neuberger  et al (2007)^[59]^ | ✓ | ✓ | 🗶 | ✓ | ✓ | ✓ | ✓ | 1: 🗶  2: 🗶  3: 🗶  4: 🗶  5: 🗶 | 6: ✓  7: 🗶  8: ✓  9: ✓  10: ✓ | ✓ | 🗶 | 🗶 | 🗶 |
| Flint-Wagner  et al (2009)^[60]^ | ✓ | ✓ | ✓ | ✓ | ✓ | ✓ | ✓ | 1: ✓  2: ✓  3: ✓  4: ✓  5: 🗶 | 6: ✓  7: 🗶  8: ✓  9: ✓  10: ✓ | ✓ | 🗶 | 🗶 | ✓ |
| Lemmey  et al (2009)^[48]^ | ✓ | ✓ | ✓ | ✓ | ✓ | ✓ | ✓ | 1: ✓  2: ✓  3: ✓  4: ✓  5: 🗶 | 6: ✓  7: ✓  8: ✓  9: ✓  10: ✓ | ✓ | 🗶 | 🗶 | ✓ |
| van Rensberg  et al (2010)^[66]^ | ✓ | ✓ | 🗶 | ✓ | ✓ | ✓ | 🗶 | 1: 🗶  2: 🗶  3: 🗶  4: 🗶  5: 🗶 | 6: ✓  7: 🗶  8: ✓  9: ✓  10: ✓ | ✓ | 🗶 | 🗶 | ✓ |
| Breedland  et al (2011)^[49]^ | ✓ | ✓ | ✓ | ✓ | ✓ | ✓ | ✓ | 1: ✓  2: ✓  3: ✓  4: ✓  5: 🗶 | 6: ✓  7: 🗶  8: ✓  9: ✓  10: ✓ | ✓ | ✓ | 🗶 | 🗶 |
| Strasser  et al (2011)^[50]^ | ✓ | ✓ | 🗶 | ✓ | ✓ | ✓ | 🗶 | 1: ✓  2: 🗶  3: ✓  4: ✓  5: 🗶 | 6: ✓  7: 🗶  8: ✓  9: ✓  10: ✓ | ✓ | 🗶 | 🗶 | ✓ |
| Rahnama  et al (2012)^[62]^ | ✓ | ✓ | 🗶 | ✓ | ✓ | ✓ | 🗶 | 1: 🗶  2: 🗶  3: 🗶  4: 🗶  5: 🗶 | 6 🗶  7: 🗶  8: 🗶  9: ✓  10: ✓ | 🗶 | 🗶 | 🗶 | 🗶 |
| van Rensberg  et al (2012)^[65]^ | ✓ | ✓ | 🗶 | ✓ | ✓ | ✓ | 🗶 | 1: ✓  2: 🗶  3: 🗶  4: 🗶  5: 🗶 | 6: 🗶  7: 🗶  8: ✓  9: ✓  10: ✓ | ✓ | 🗶 | 🗶 | ✓ |
| Cima  et al (2013)^[67]^ | ✓ | ✓ | ✓ | ✓ | ✓ | ✓ | ✓ | 1: ✓  2: ✓  3: 🗶  4: 🗶  5: 🗶 | 6: 🗶  7: 🗶  8: ✓  9: ✓  10: ✓ | ✓ | 🗶 | 🗶 | 🗶 |
| Dogu  et al (2013)^[51]^ | ✓ | ✓ | ✓ | ✓ | ✓ | ✓ | ✓ | 1: ✓  2: ✓  3: ✓  4: ✓  5: 🗶 | 6: 🗶  7: ✓  8: 🗶  9: ✓  10: ✓ | 🗶 | 🗶 | 🗶 | 🗶 |
| Durcan  et al (2014)^[52]^ | ✓ | ✓ | ✓ | ✓ | ✓ | ✓ | ✓ | 1: 🗶  2: ✓  3: ✓  4: ✓  5: 🗶 | 6: ✓  7: ✓  8: 🗶  9: ✓  10: ✓ | ✓ | 🗶 | 🗶 | 🗶 |
| Jahanbin  et al (2014)^[63]^ | ✓ | ✓ | 🗶 | ✓ | ✓ | ✓ | 🗶 | 1: 🗶  2: 🗶  3: 🗶  4: 🗶  5: 🗶 | 6: 🗶  7: 🗶  8: 🗶  9: ✓  10: ✓ | 🗶 | 🗶 | 🗶 | 🗶 |
| Manning  et al (2014)^[53]^ | ✓ | ✓ | ✓ | ✓ | ✓ | ✓ | ✓ | 1: ✓  2: ✓  3: ✓  4: ✓  5: 🗶 | 6: ✓  7: ✓  8: ✓  9: ✓  10: ✓ | ✓ | ✓ | ✓ | ✓ |
| Lamb  et al (2015)^[54]^ | ✓ | ✓ | ✓ | ✓ | ✓ | ✓ | ✓ | 1: ✓  2: ✓  3: ✓  4: ✓  5: 🗶 | 6: ✓  7: 🗶  8: ✓  9: ✓  10: ✓ | ✓ | ✓ | ✓ | ✓ |
| Seneca  et al (2015)^[55]^ | ✓ | ✓ | 🗶 | ✓ | ✓ | ✓ | ✓ | 1: 🗶  2: 🗶  3: ✓  4: ✓  5: 🗶 | 6: ✓  7: ✓  8: ✓  9: ✓  10: ✓ | ✓ | ✓ | 🗶 | ✓ |
| Dulgeroglu  et al (2016)^[56]^ | ✓ | ✓ | ✓ | ✓ | 🗶 | ✓ | ✓ | 1: ✓  2: ✓  3: 🗶  4: ✓  5: ✓ | 6: 🗶  7: 🗶  8: 🗶  9: ✓  10: ✓ | 🗶 | 🗶 | 🗶 | 🗶 |
| Tonga  et al (2016)^[57]^ | ✓ | ✓ | 🗶 | ✓ | 🗶 | ✓ | 🗶 | 1: 🗶  2: 🗶  3: 🗶  4: 🗶  5: 🗶 | 6: 🗶  7: 🗶  8: 🗶  9: 🗶  10: 🗶 | 🗶 | 🗶 | 🗶 | 🗶 |
| Lourenzi  et al (2017)^[68]^ | ✓ | ✓ | ✓ | ✓ | ✓ | ✓ | ✓ | 1: ✓  2: ✓  3: ✓  4: ✓  5: 🗶 | 6: ✓  7: ✓  8: ✓  9: ✓  10: ✓ | ✓ | ✓ | 🗶 | ✓ |
| Shinde &  Varadharajulu  (2017)^[69]^ | ✓ | ✓ | 🗶 | ✓ | 🗶 | 🗶 | 🗶 | 1: 🗶  2: 🗶  3: 🗶  4: ✓  5: 🗶 | 6: 🗶  7: 🗶  8: 🗶  9: ✓  10: ✓ | 🗶 | 🗶 | 🗶 | 🗶 |
| Anvar  et al (2018)^[64]^ | ✓ | ✓ | 🗶 | ✓ | ✓ | ✓ | ✓ | 1: 🗶  2: 🗶  3: 🗶  4: 🗶  5: 🗶 | 6: 🗶  7: 🗶  8: 🗶  9: ✓  10: ✓ | 🗶 | 🗶 | 🗶 | 🗶 |
| Lange  et al (2018)^[58]^ | ✓ | ✓ | ✓ | ✓ | ✓ | ✓ | ✓ | 1: 🗶  2: ✓  3: ✓  4: ✓  5: 🗶 | 6: ✓  7: 🗶  8: ✓  9: ✓  10: ✓ | ✓ | ✓ | 🗶 | ✓ |
| Mohanty  et al (2018)^[70]^ | ✓ | ✓ | 🗶 | ✓ | 🗶 | ✓ | ✓ | 1: 🗶  2: 🗶  3: 🗶  4: ✓  5: 🗶 | 6: 🗶  7: ✓  8: 🗶  9: ✓  10: ✓ | 🗶 | 🗶 | 🗶 | 🗶 |
| Piva  et al (2018)^[61]^ | ✓ | ✓ | ✓ | ✓ | ✓ | ✓ | ✓ | 1: ✓  2: ✓  3: ✓  4: ✓  5: 🗶 | 6: ✓  7: ✓  8: ✓  9: ✓  10: ✓ | ✓ | ✓ | 🗶 | ✓ |
| % Completion | **100%** | **100%** | **50%** | **97%** | **78%** | **97%** | **75%** | **1: 43%**  **2: 54%**  **3: 46%**  **4: 66%**  **5: 6%** | **6: 50%**  **7: 37%**  **8: 63%**  **9: 97%**  **10: 97%** | **66%** | **28%** | **9%** | **53%** |

✓ = Item sufficiently described in the trial. 🗶 = Inadequately or not described.

Item 8

| 1 = Exercise type  2 = Strength equipment used  3 = Sets  4 = Repetitions  5 = Load (kg/lbs) | 6 = Intensity (%1RM/Borg)  7 = Recovery  8 = Progression  9 = Frequency  10 = Programme duration |
| --- | --- |
