# Supplementary material for: A systematic review exploring the evidence reported to underpin exercise dose in clinical trials of rheumatoid arthritis
Source: Rheumatology (Oxford). 2020 Aug 11;59(11):3147–57. doi: 10.1093/rheumatology/keaa150 (PMC7590408; doi:10.1093/rheumatology/keaa150)
Supplement: keaa150_supplementary_data [file keaa150_supplementary_data.zip › Supplementary table S4_GB05082020docx.docx]

Consistency in dose parameters comparing RCT against its respective pilot study.

| **Dose parameter >** | **Type of strength exercise** | **Sets** | **Repetitions** | **Load** | **Intensity** | **Method of recovery** | **Method of progression** | **Frequency of sessions** | **Programme**  **duration** | **Consistency rating** |
| --- | --- | --- | --- | --- | --- | --- | --- | --- | --- | --- |
| **RCT/Pilot**  **v** |  |  |  |  |  |  |  |  |  |  |
| **RCT:**  Neuberger  et al  (2007)  ^[59]^ | Insufficiently  described | Insufficiently  described | Insufficiently  described | Insufficiently  described | 60-80%1RM | Insufficiently  described | Each participant was given their target HR for 60% & 80% MHR and instructed to start exercising at 60% and progress to 80% as tolerated given their subjective exertion using the Talk Test (being able to talk while exercising without being short of breath) and the Borg scale. | 3 x week | 12 weeks | Exercise type:  Unclear  Sets:  Unclear  Repetitions:  Unclear  Load:  Unclear  Intensity:  Unclear  Recovery:  Unclear  Progression:  Inconsistent  Frequency:  Consistent  Duration:  Consistent |
| **Pilot study:**  Neuberger  et al  (1997)  ^[75]^ | Insufficiently  described | Insufficiently  described | Insufficiently  described | Insufficiently  described | Insufficiently  described | Insufficiently  described | Initially warm-up and strengthening phases were longer in order to build muscle strength. As aerobic minutes were increased, minutes of warm-up and cool-down were reduced. | 3 x week | 12 weeks |  |
| **RCT:**  Lemmey  et al  (2009)  ^[48]^ | 1. Chest press 2. Seated leg extension 3. Rowing 4. Bicep curl 5. Triceps extension 6. Leg press 7. Leg curl 8. Standing calf raises | Week 1:  1 set  Week 2:  2 sets  Weeks 3-24  3 sets | Weeks 1-4:  15 repetitions  Weeks 5-6:  12 repetitions  Weeks 7-24:  8 repetitions | Insufficiently  described | Weeks 1-4: 60%1RM  Weeks 5-6: 70%1RM  Weeks 7-24: 80%1RM | 1-2 minutes between sets | 1RM reassessed  every 4 weeks | 2 x week | 24 weeks | Exercise type:  Consistent  Sets:  Inconsistent  Repetitions:  Inconsistent  Load:  Unclear  Intensity:  Inconsistent  Recovery:  Consistent  Progression:  Inconsistent  Frequency:  Inconsistent  Duration:  Inconsistent |
| **Pilot study:**  Marcora  et al  (2005)  ^[76]^ | 1. Chest press 2. Seated leg extension 3. Seated row 4. Bicep curl 5. Triceps extension 6. Leg press 7. Leg curl 8. Standing calf raises | 4 sets | Set 1:  15 repetitions  Sets 2-4:  8 repetitions  (Repetition velocity:  1-2 seconds concentric/  Eccentric) | Insufficiently  described | Set 1:  40%1RM  Sets 2-4:  80%1RM | 1-2 minutes between sets and exercises  +  48 hours between training sessions | 1RM reassessed at end of week 0 then every 2 weeks | 3 x week | 12 weeks |  |
| **RCT:**  Manning  et al  (2014)  ^[53]^ | 6 out of 16 exercises were used:   1. Putty ball squeeze 2. Putty fingertip pinch 3. Putty finger hook and squeeze 4. Knife and fork putty cutting 5. Paper clips and envelope challenge 6. Wrist alphabet with band 7. Back scrub 8. Up and out of chair 9. Arm curl with band 10. Lift to chin with band 11. Reach back with band 12. Side Lift with band 13. Wall wash squares with band 14. Door push with band 15. Shoulder rotation with band 16. Reach to shelf with band | Between  1-3 sets | 8-12 repetitions | Insufficiently  described | Participants were encouraged to maintain an RPE of 13–17 (equivalent to 50–80% of maximal exertion). | 30 seconds between sets | Maintain Borg rating of perceived value at 13-17 (50-80% of max exertion) | Weeks 1-2  2 x week with the physio  Weeks 3-12  Self-supervised  1 x daily  at home | 12 weeks | Exercise type:  Unclear  Sets:  Unclear  Repetitions:  Unclear  Load:  Unclear  Intensity:  Unclear  Recovery:  Unclear  Progression:  Unclear  Frequency:  Unclear  Duration:  Unclear |
| **Pilot study:**  Unpublished | Insufficiently  described | Insufficiently  described | Insufficiently  described | Insufficiently  described | Insufficiently  described | Insufficiently  described | Insufficiently  described | Insufficiently  described | Insufficiently  described |  |
| **RCT:**  Lamb  et al  (2015)  ^[54]^ | 1. Eccentric wrist extension 2. Gross grip 3. Finger adduction 4. Pinch grip | Between  1-3 sets | 8-30 repetitions | Insufficiently  described | 3-4 to 5-6  on  modified Borg scale | Insufficiently  described | Permitted to progress up to 30 repetitions + increase load using RPE scale | 1 x daily | 12 weeks | Exercise type:  Consistent  Sets:  Consistent  Repetitions:  Consistent  Load:  Unclear  Intensity:  Consistent  Recovery:  Unclear  Progression:  Consistent  Frequency:  Consistent  Duration:  Consistent |
| **Pilot study:**  **(reported in)**  Heine et al (2012)  **^[73]^**  +  Williams  et al  (2015)  ^[74]^ | 1. Eccentric wrist extension 2. Gross grip 3. Finger adduction 4. Pinch grip | Between  1-3 sets | 8-30 repetitions | Insufficiently  described | 3-4 to 5-6  on  modified Borg scale | Insufficiently  described | Permitted to progress up to 30 repetitions + increase load using RPE scale | 1 x daily | 12 weeks |  |
