# Supplementary material for: A systematic review exploring the evidence reported to underpin exercise dose in clinical trials of rheumatoid arthritis
Source: Rheumatology (Oxford). 2020 Aug 11;59(11):3147–57. doi: 10.1093/rheumatology/keaa150 (PMC7590408; doi:10.1093/rheumatology/keaa150)
Supplement: keaa150_supplementary_data [file keaa150_supplementary_data.zip › Supplementary table S5_GB05082020.docx]

Quality, consistency and applicability of the underpinning evidence.

| RCT | Underpinning evidence | Quality | Consistency | | | | | | | | | Applicability | | |
| --- | --- | --- | --- | --- | --- | --- | --- | --- | --- | --- | --- | --- | --- | --- |
|  |  | ***OCEBM level*** | ***Exercise*** | ***Sets*** | ***Reps*** | ***Load*** | ***Intensity*** | ***Recovery*** | ***Progress*** | ***Frequency*** | ***Duration*** | ***RA*** | ***Gender*** | ***Age*** |
| Neuberger (2007)^[59]^ | ACSM (1991)^[86]^ | ‘Unclear’ |  |  |  |  |  |  |  |  |  |  |  |  |
|  | Neuberger (1997)^[75]^ | ‘3’ |  |  |  |  |  |  |  |  |  |  |  |  |
| Flint-Wagner (2009)^[60]^ | Baechle (2000)^[85]^ | ‘3-5’ |  |  |  |  |  |  |  |  |  |  |  |  |
|  | Borg (1998)^[87]^ | ‘Unclear’ |  |  |  |  |  |  |  |  |  |  |  |  |
| Lemmey (2009)^[48]^ | ACSM (2002)^[36]^ | ‘2-5’ |  |  |  |  |  |  |  |  |  |  |  |  |
|  | Marcora (2005)^[76]^ | ‘2-5’ |  |  |  |  |  |  |  |  |  |  |  |  |
| Strasser (2011)^[50]^ | Williams (2007)^[88]^ | ‘Unclear’ |  |  |  |  |  |  |  |  |  |  |  |  |
| van Rensburg (2012)^[65]^ | Iversen (2002)^[89]^ | ‘Unclear’ |  |  |  |  |  |  |  |  |  |  |  |  |
|  | ACSM (2006) | ‘Incorrect citation’ |  |  |  |  |  |  |  |  |  |  |  |  |
| Durcan (2014)^[52]^ | ACSM (2009)^[90]^ | ‘Unclear’ |  |  |  |  |  |  |  |  |  |  |  |  |
|  | ACSM (2011)^[82]^ | ‘2-5’ |  |  |  |  |  |  |  |  |  |  |  |  |
| Manning (2014)^[53]^ | Hurley et al (2007)^[72]^ | ‘2’ |  |  |  |  |  |  |  |  |  |  |  |  |
| Lamb (2015)^[54]^ | Borg (1982)^[81]^ | ‘5’ |  |  |  |  |  |  |  |  |  |  |  |  |
|  | Hoenig (1993)^[77]^ | ‘2’ |  |  |  |  |  |  |  |  |  |  |  |  |
|  | ACSM (2002)^[36]^ | ‘2-5’ |  |  |  |  |  |  |  |  |  |  |  |  |
|  | McGuigan (2004)^[78]^ | ‘2’ |  |  |  |  |  |  |  |  |  |  |  |  |
|  | Marcora (2005)^[76]^ | ‘3’ |  |  |  |  |  |  |  |  |  |  |  |  |
|  | O’Brien (2006)^[39]^ | ‘2’ |  |  |  |  |  |  |  |  |  |  |  |  |
| Seneca (2015)^[55]^ | Dept PT/OT (2015)^[91]^ | ‘Unclear’ |  |  |  |  |  |  |  |  |  |  |  |  |
| Dulgeroglu (2016)^[56]^ | Pelland (2002)^[92]^ | ‘Incorrect citation’ |  |  |  |  |  |  |  |  |  |  |  |  |
|  | Ronnigen (2008)^[80]^ | ‘3’ |  |  |  |  |  |  |  |  |  |  |  |  |
| Lourenzi (2017)^[68]^ | Hicks (1994)^[83]^ | ‘2-5’ |  |  |  |  |  |  |  |  |  |  |  |  |
|  | Bearne (2002)^[43]^ | ‘2’ |  |  |  |  |  |  |  |  |  |  |  |  |
|  | ACSM (2009)^[84]^ | ‘2-4’ |  |  |  |  |  |  |  |  |  |  |  |  |
| Piva (2018)^[61]^ | Rall (1996)^[79]^ | ‘2’ |  |  |  |  |  |  |  |  |  |  |  |  |
|  | van den Ende (2000)^[40]^ | ‘2’ |  |  |  |  |  |  |  |  |  |  |  |  |
|  | Hakkinen (2001)^[42]^ | ‘2’ |  |  |  |  |  |  |  |  |  |  |  |  |

Key:

Inconsistent/not applicable

Unclear as insufficiently described/not described

Consistent/applicable

Source not used to underpin dose parameter
