# Supplementary material for: A systematic review exploring the evidence reported to underpin exercise dose in clinical trials of rheumatoid arthritis
Source: Rheumatology (Oxford). 2020 Aug 11;59(11):3147–57. doi: 10.1093/rheumatology/keaa150 (PMC7590408; doi:10.1093/rheumatology/keaa150)
Supplement: keaa150_supplementary_data [file keaa150_supplementary_data.zip › Supplementary table S6_GB05082020.docx]

Risk of bias and level of underpinning secondary evidence for primary evidence sources.

| **RCT** | Sequence generation | Allocation concealment | Blinding of participants and personnel | Blinding of outcome assessors for all outcomes | Incomplete outcome data for all outcomes | Selective outcome reported | Other sources of bias | Overall risk of bias rating | No of underpinning evidence sources | Source 1 - OCEBM level of evidence | Source 2 - OCEBM level of evidence | Source 3 - OCEBM level of evidence | Source 4 - OCEBM level of evidence | Source 5 - OCEBM level of evidence | Source 6 - OCEBM level of evidence |
| --- | --- | --- | --- | --- | --- | --- | --- | --- | --- | --- | --- | --- | --- | --- | --- |
| Neuberger et al (2007)^[59]^ |  |  |  |  |  |  |  |  | 2 | Unclear^[86]^ | 3^[75]^ |  |  |  |  |
| Flint-Wagner et al (2009)^[60]^ |  |  |  |  |  |  |  |  | 2 | 3-5^[85]^ | Unclear^[87]^ |  |  |  |  |
| Lemmey et al (2009)^[48]^ |  |  |  |  |  |  |  |  | 2 | 2-5^[36]^ | 2-5^[76]^ |  |  |  |  |
| Strasser et al (2011)^[50]^ |  |  |  |  |  |  |  |  | 1 | Unclear^[88]^ |  |  |  |  |  |
| van Rensburg et al (2012)^[65]^ |  |  |  |  |  |  |  |  | 2 | Unclear^[89]^ | Incorrect |  |  |  |  |
| Durcan et al (2014)^[52]^ |  |  |  |  |  |  |  |  | 2 | Unclear^[90]^ | 2-5^[82]^ |  |  |  |  |
| Manning et al (2014)^[53]^ |  |  |  |  |  |  |  |  | 1 | 2^[72]^ |  |  |  |  |  |
| Lamb et al (2015)^[54]^ |  |  |  |  |  |  |  |  | 6 | 5^[81]^ | 2^[77]^ | 2-5^[36]^ | 2^[78]^ | 3^[76]^ | 2^[39]^ |
| Seneca et al (2015)^[55]^ |  |  |  |  |  |  |  |  | 1 | Unclear^[91]^ |  |  |  |  |  |
| Dulgeroglu et al (2016)^[56]^ |  |  |  |  |  |  |  |  | 2 | Incorrect^[92]^ | 3^[80]^ |  |  |  |  |
| Lourenzi et al (2017)^[68]^ |  |  |  |  |  |  |  |  | 3 | 2-5^[83]^ | 2^[43]^ | 2-4^[84]^ |  |  |  |
| Piva et al (2018)^[61]^ |  |  |  |  |  |  |  |  | 3 | 2^[79]^ | 2^[40]^ | 2^[42]^ |  |  |  |
